# Evaluating the population impact of hepatitis C direct acting antiviral treatment as prevention for people who inject drugs (EPIToPe) – a natural experiment (protocol)

**DOI:** 10.1136/bmjopen-2019-029538

**Published:** 2019-09-24

**Authors:** Matthew Hickman, John F Dillon, Lawrie Elliott, Daniela De Angelis, Peter Vickerman, Graham Foster, Peter Donnan, Ann Eriksen, Paul Flowers, David Goldberg, William Hollingworth, Samreen Ijaz, David Liddell, Sema Mandal, Natasha Martin, Lewis J Z Beer, Kate Drysdale, Hannah Fraser, Rachel Glass, Lesley Graham, Rory N Gunson, Emma Hamilton, Helen Harris, Magdalena Harris, Ross Harris, Ellen Heinsbroek, Vivian Hope, Jeremy Horwood, Sarah Karen Inglis, Hamish Innes, Athene Lane, Jade Meadows, Andrew McAuley, Chris Metcalfe, Stephanie Migchelsen, Alex Murray, Gareth Myring, Norah E Palmateer, Anne Presanis, Andrew Radley, Mary Ramsay, Pantelis Samartsidis, Ruth Simmons, Katy Sinka, Gabriele Vojt, Zoe Ward, David Whiteley, Alan Yeung, Sharon J Hutchinson

**Affiliations:** 1 Population Health Sciences, Bristol Medical School, Bristol, Bristol, UK; 2 Hepatology & Gastroenterology, Clinical & Molecular Medicine, School of Medicine, University of Dundee, Dundee, UK; 3 Glasgow Caledonian University, Glasgow, UK; 4 MRC Biostatistics Unit, School of Clinical Medicine, University of Cambridge, Cambridge, UK; 5 Blizard Institute, Queen Mary University of London, London, UK; 6 Barts Health NHS Trust, London, UK; 7 Dundee Epidemiology and Biostatistics Unit, University of Dundee, Dundee, UK; 8 Tayside Health Board, Dundee, UK; 9 Health Protection Scotland, Glasgow, UK; 10 National Infection Service, Public Health England, London, UK; 11 Scottish Drugs Forum, Edinburgh, UK; 12 Division of Infectious Diseases and Global Public Health, University of California San Diego, San Diego, UK; 13 Tayside Clinical Trials Unit, Tayside Medical Science Centre, University of Dundee, Dundee, UK; 14 ISD Scotland, Edinburgh, UK; 15 West Of Scotland Specialist Virology Centre, NHS Greater Glasgow & Clyde Board, Glasgow, UK; 16 Scottish Drug Forum, Edinburgh, UK; 17 London School of Hygiene and Tropical Medicine, London, UK; 18 Liverpool John Moores University, Liverpool, UK; 19 Directorate of Public Health, NHS Tayside, Dundee, UK; 20 Edinburgh Napier University, Edinburgh, UK

**Keywords:** Hepatology, INFECTIOUS DISEASES, Epidemiology, PUBLIC HEALTH, Public health, Infection control

## Abstract

**Introduction:**

Hepatitis C virus (HCV) is the second largest contributor to liver disease in the UK, with injecting drug use as the main risk factor among the estimated 200 000 people currently infected. Despite effective prevention interventions, chronic HCV prevalence remains around 40% among people who inject drugs (PWID). New direct-acting antiviral (DAA) HCV therapies combine high cure rates (>90%) and short treatment duration (8 to 12 weeks). Theoretical mathematical modelling evidence suggests HCV treatment scale-up can prevent transmission and substantially reduce HCV prevalence/incidence among PWID. Our primary aim is to generate empirical evidence on the effectiveness of HCV ‘Treatment as Prevention’ (TasP) in PWID.

**Methods and analysis:**

We plan to establish a natural experiment with Tayside, Scotland, as a single intervention site where HCV care pathways are being expanded (including specialist drug treatment clinics, needle and syringe programmes (NSPs), pharmacies and prison) and HCV treatment for PWID is being rapidly scaled-up. Other sites in Scotland and England will act as potential controls. Over 2 years from 2017/2018, at least 500 PWID will be treated in Tayside, which simulation studies project will reduce chronic HCV prevalence among PWID by 62% (from 26% to 10%) and HCV incidence will fall by approximately 2/3 (from 4.2 per 100 person-years (p100py) to 1.4 p100py). Treatment response and re-infection rates will be monitored. We will conduct focus groups and interviews with service providers and patients that accept and decline treatment to identify barriers and facilitators in implementing TasP. We will conduct longitudinal interviews with up to 40 PWID to assess whether successful HCV treatment alters their perspectives on and engagement with drug treatment and recovery. Trained peer researchers will be involved in data collection and dissemination. The primary outcome – chronic HCV prevalence in PWID – is measured using information from the Needle Exchange Surveillance Initiative survey in Scotland and the Unlinked Anonymous Monitoring Programme in England, conducted at least four times before and three times during and after the intervention. We will adapt Bayesian synthetic control methods (specifically the Causal Impact Method) to generate the cumulative impact of the intervention on chronic HCV prevalence and incidence. We will use a dynamic HCV transmission and economic model to evaluate the cost-effectiveness of the HCV TasP intervention, and to estimate the contribution of the scale-up in HCV treatment to observe changes in HCV prevalence. Through the qualitative data we will systematically explore key mechanisms of TasP real world implementation from provider and patient perspectives to develop a manual for scaling up HCV treatment in other settings. We will compare qualitative accounts of drug treatment and recovery with a ‘virtual cohort’ of PWID linking information on HCV treatment with Scottish Drug treatment databases to test whether DAA treatment improves drug treatment outcomes.

**Ethics and dissemination:**

Extending HCV community care pathways is covered by ethics (ERADICATE C, ISRCTN27564683, Super DOT C Trial clinicaltrials.gov: NCT02706223). Ethical approval for extra data collection from patients including health utilities and qualitative interviews has been granted (REC ref: 18/ES/0128) and ISCRCTN registration has been completed (ISRCTN72038467). Our findings will have direct National Health Service and patient relevance; informing prioritisation given to early HCV treatment for PWID. We will present findings to practitioners and policymakers, and support design of an evaluation of HCV TasP in England.

**Strengths and limitations of this study**

- Our control sites in the rest of Scotland and England were not randomised so there will be confounding and uncertainty in the intervention effect estimates.
- Hepatitis C virus treatment and prevention strategy in UK (and Europe) is evolving - motivated both by WHO ‘elimination targets’ and falling drug prices – which may contaminate our controls.
- However, our statistical models suggest that we should have sufficient power to detect an intervention effect and can model changes over time.
- We will develop dynamic transmission and economic models that can estimate cost-effectiveness including the prevention benefit of this intervention.
- We are conducting multiple nested qualitative studies and training and using peer researchers.

## Introduction and background

Infection with hepatitis C virus (HCV) is a progressive disease that over 20 to 40 years can lead to liver cancer and premature death. HCV is the second largest contributor to liver disease in the UK and one of the few causes that is curable.^1^ In the UK it is estimated that approximately 200 000 people are infected with HCV, over 85% of whom are people who inject or have injected drugs (PWID).^2–5^ Chronic HCV prevalence and incidence among PWID remains high in UK at 20% to 50% and 5 to 15 per 100 person-years, respectively.^4 6–18^ Prevention of HCV transmission among PWID is critical to long-term prevention of HCV related liver disease.^19^

We have reviewed the effectiveness of traditional primary prevention against HCV – opioid substitution treatment (OST) and needle and syringe programmes (NSPs).^12 20–22^ Ongoing exposure to OST and high-coverage NSPs can reduce the risk of HCV transmission by 50% to 80%.^12 22^ In Scotland HCV incidence among PWID decreased from approximately 14 to 6 per 100 person-years from 2008/2009 to 2011/2012 coinciding with the launch of the Scottish HCV strategy and action plan which incorporated scale-up of harm reduction interventions and HCV treatment.^10 23^ We estimated that 60% of this decline could be attributed to the scale-up of OST and NSP during the action plan and that 1400 HCV infections were averted by 2015.^24^ However, there was no appreciable reduction in overall anti-HCV prevalence over this short period, and there is some suggestion that incidence has increased recently to ~10 per 100 person-years (http://www.hps.scot.nhs.uk/resourcedocument.aspx?id=5863). HCV transmission models suggest that primary prevention through NSP and OST alone is insufficient to achieve substantial reductions (of the order of 40% or more within 10 years) in HCV prevalence among PWID in the UK.^25 26^

Prevention of hepatitis C disease and HCV transmission is now possible because highly effective, tolerable, short-course interferon-free direct-acting antiviral therapies (DAAs) are available for all HCV genotypes with cure rates – defined as sustained virological response (SVR) - exceeding 90%.^27–29^ We, and others, hypothesise that HCV treatment scale-up for PWID, and resulting HCV Treatment as Prevention (TasP) could enhance other primary interventions and reduce HCV incidence and chronic prevalence to negligible levels (ie, towards elimination as a major public health concern).^30–35^ TasP refers to the concept whereby future transmission is reduced by treating affected individuals^36 37^: in HIV TasP antiretroviral treatment reduces transmission because individuals have undetectable infection^38^ in HCV TasP people are cured so reducing opportunities for future transmission. WHO targets for HCV elimination, adopted by UK and other countries, aim to reduce HCV incidence by 80% and associated mortality by 65% by 2030.^39–44^

Clinical guidelines in Europe and USA changed from recommending prioritising HCV treatment to people with moderate-to-severe liver disease towards removing any restrictions and recommending that people at risk of transmission irrespective of fibrosis stage are offered treatment.^45–49^ Cost-effectiveness models that incorporate the population prevention benefit suggest early treatment should be prioritised to PWID over other patient groups (unless chronic HCV prevalence and transmission is very high).^50^ There is direct evidence that SVR following HCV treatment reduces liver disease progression and mortality risk,^51–53^ but in two recent reviews we found no empirical evidence that HCV treatment scale-up has reduced chronic HCV prevalence and incidence in PWID populations.^36 37^ In part this is because in most settings HCV treatment rates in PWID are too low and any changes generally too small to be detected, as we show in two studies of seven sites in UK^7^ and an extension to 11 sites in Europe.^54^ Until very recently in the UK, the annual number of HCV DAA treatments was restricted - as drug costs could be expensive (>£10 000 per patient). There is the opportunity now to test whether scaling up HCV treatment will reduce chronic HCV prevalence and transmission among PWID.^44^

In a pilot study (‘Eradicate C’) in Tayside we showed that we can increase HCV case-finding and engage and successfully treat PWID in the community.^55^ Combining further studies on extending community HCV treatment pathways in Tayside and additional treatments provided by National Health Service (NHS) Tayside and Scottish Government we can establish an immediate natural experiment (with Tayside as the intervention site and other sites in Scotland and England as controls) to test and generate UK empirical evidence on the and potential impact and cost-effectiveness of HCV TasP in PWID. The UK is one of few countries worldwide to have an established nationwide surveillance system monitoring HCV infection among PWID.^9 12 17 22 56–60^ This is undertaken through a series of cross-sectional voluntary anonymous surveys of PWID recruited at harm reduction services, referred to as the Unlinked Anonymous Monitoring Programme (UAM) in England and Wales and the Needle Exchange Surveillance Initiative (NESI) in Scotland.^61 62^ In addition, the UK has established sentinel laboratory surveillance of HCV testing and national monitoring of HCV treatment.^8 63–65^ The data collected in both UAM and NESI will be used to assess out outcome.

Alongside a natural experiment in Tayside, we will collect information to assess the treatment facilitators and barriers. Historically it has proven very hard to engage PWID in HCV treatment.^66–69^ Some barriers to engagement, such as poor efficacy or fear of interferon treatment side-effects, may be ameliorated by DAA therapy. However, other barriers such as mistrust of health services, stigma and competing priorities faced by PWID may persist. In addition, providers may be reticent to refer or provide HCV treatment to PWID due to concerns about adherence, reinfection and perceptions of treatment ‘worth’.^70 71^ It is expected that co-locating HCV treatment within existing services will reduce many system and provider level barriers to PWID accessing care.^66–68 72–77^ However, this has not been tested in the context of community wide scale-up of interventions across multiple potential pathways. It is critical, therefore, that we understand how HCV TasP is embedded within the existing service landscape and incorporated into providers’ professional roles.

Finally it has been hypothesised that successful HCV treatment in PWID may positively impact on understandings of self and identity and improve treatment of drug use disorders.^71 72 78–80^ Accounts of ‘transformative’ outcomes extending beyond viral clearance alone include reference to reductions in drug and alcohol use, uptake of safer injecting practices, improved social relationships, enhanced sense of responsibility and self-worth. Hints of such collateral or indirect benefits are also found in quantitative studies reporting low re-infection rates and reductions in risky injecting behaviours among treated PWID.^81 82^ We aim to test this hypothesis in our qualitative follow-up study and compare the findings to quantitative data generated from a virtual cohort.

## Methods and analysis

### Study design

Our intention is to create and conduct a mixed methods study, including qualitative studies and economic evaluation, of a natural experiment of HCV TasP among PWID. We also will develop methods for evaluating HCV TasP.

### Methods

#### Scaling-up HCV treatment

**Figure 1:**
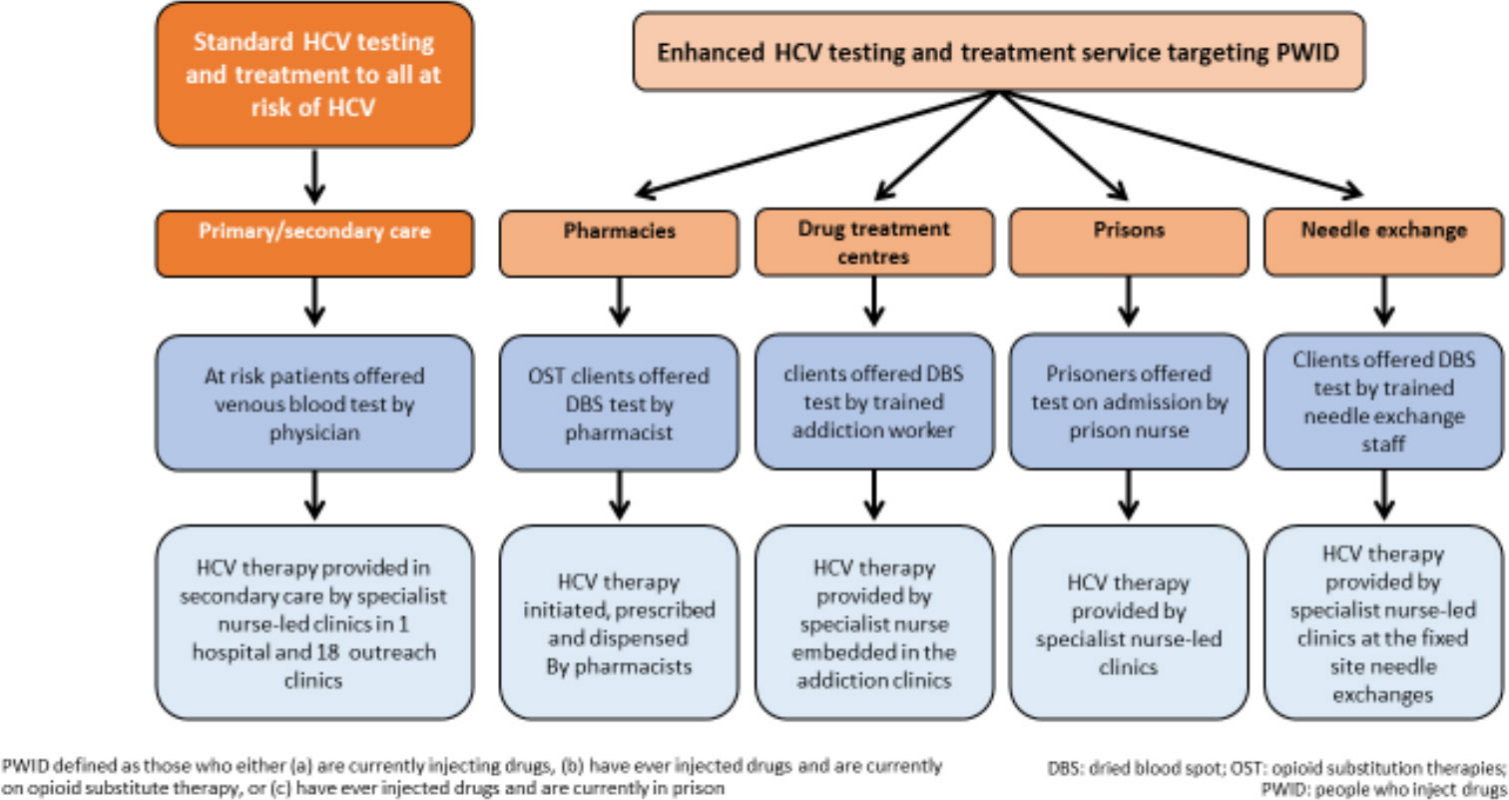
Overview of HCV testing and treatment pathways for the PWID population in NHS Tayside. DBS, dried blood spot; HCV, hepatitis C virus; NHS, National Health Service; OST, opioid substitution treatment; PWID, people who inject drugs.

The **intervention** comprises the scale-up of HCV treatment in PWID which has started early in Tayside. By combining support from Scottish Government, National Health Board Tayside (NHS Tayside) and industry (MSD, Gilead, BMS) we can deliver rapid intensive scale-up of HCV treatments for PWID (comprising an extra 400 HCV treatments, a 3.5-fold increase from treatments for PWID prior to April 2017, see sample size below). We have developed multiple integrated community HCV care pathways, including novel care pathways in pharmacies, a low threshold NSP, drug treatment services and prisons (see figure 1). Our diagnostic pathways make extensive use of dried blood spot (DBS) testing for diagnosis of HCV antibody and chronic HCV with subsequent conventional laboratory testing in preparation for treatment (viral load, liver function and FIB-4 fibrosis score).^83–85^

#### Study population

Our intervention is delivered and measured at the population level – which we have created by combining several individual studies and treatment pathways as shown in figure 1 (see ethics section below for the individual studies). We gained ethical approval from East of Scotland Research Ethics Service REC 1 (ref: 18/ES/0128) to ask patients for permission to be recruited into the qualitative study (below) and extended clinical and behavioural drug history and data on health utilities (EQ5D-5L) at onset of treatment, during treatment and after the end of treatment.

Community HCV specialist nurses (3.5 full-time equivalent (FTE)) coordinate and deliver case-finding and treatment across the pathways in Tayside (figure 1).

The region of Tayside co-localises to NHS Tayside which is the provider of healthcare to a geographical area of 2903 sq mi (7519 km^2^) including the cities of Dundee and Perth and the counties of Angus and Perth & Kinross, situated in the east of Scotland with a population of 416 000. It is a mixture of urban and rural environments with some of the most affluent and most deprived areas in Scotland. It is therefore a representative microcosm of many areas in the UK.

#### HCV treatment

Apart from expansion of community HCV care pathways, no new clinical procedures will be investigated and all PWID with chronic HCV will be offered oral DAA HCV treatment compliant with the Scottish clinical guidelines (https://www.hps.scot.nhs.uk/resourcedocument.aspx?id=6621).

As per local standard of care, participants will be offered appropriate harm reduction advice.

Standard care for patients is to test for SVR at 12 weeks after end of treatment with patients being recommended for annual follow-up if at risk of re-infection. Specialist nurses concentrate on building a good relationship with the participant to ensure that they do return for follow-up appointments. Health Protection Scotland collates national public health surveillance data on the number, characteristics and response of patients initiated onto HCV therapy, through Clinical Databases installed in 17 specialist HCV treatment centres, across Scotland.^41 86^ A similar system also is available in England.

#### HCV surveillance and intervention outcome (chronic HCV in PWID)

The **outcome** is chronic HCV prevalence (HCV viraemia as measured by HCV PCR) among PWID in the community (not just in the patients who undergo HCV treatment). Prevalence will be monitored using the NESI and UAM surveys, as detailed below. During 2017 to 2022, three waves of data collection for NESI (n=7500) and five to six for UAM (n=17 000 in England) will measure this outcome.

In our pre-intervention period from 2010/2011 to 2016 there have been four NESI surveys in Scotland (n=10 000 participants in total) and six UAM surveys in England (n=16 000 in total), which have involved the collection of DBS linked to questionnaire data. Participants are recruited at sentinel sites by a team of trained interviewers in Scotland (at over 100 NSP sites) and by agency staff in over 60 low-threshold drug agencies across England.^58 61^ Participants complete a short questionnaire, with common questions across UAM and NESI, on demographics, injecting behaviour and service utilisation, and importantly (in relation to quantifying the intervention effect) both survey approaches have remained consistent over time.

**Figure 2:**
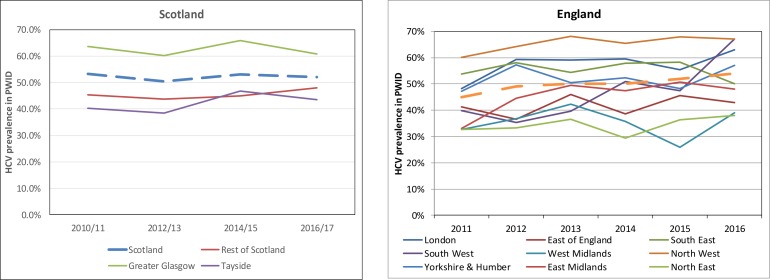
Trends in HCV antibody prevalence among PWID in Scotland and England 2010/2011 to 2016. HCV, hepatitisC virus; PWID, people who inject drugs.

The DBS samples collected in NESI and UAM have all been tested for HCV antibody, using the same methods (where sensitivity and specificity of the assay on DBS are close to 100%),^83 84^ and illustrate that antibody prevalence (ever infection) has remained relatively stable among PWID during this time (figure 2). PCR positivity among antibody positive samples is used to determine chronic infection.

All NESI and UAM samples will be tested for HCV antibody and RNA PCR to assess the impact of HCV therapy scale-up – which is critical as trends in chronic infection and antibody status will diverge as more people are cured. In addition, we will undertake RNA PCR testing of all historical samples that were HCV antibody positive shown in figure 2 so that we can measure chronic HCV prevalence among PWID pre-intervention, as well as post-intervention, for analysis (below)

Data on HCV PCR positivity among antibody negative samples identify recent infections and is used to estimate HCV incidence – which has fluctuated between 5 to 10 infections per 100 person-years across the UK during the last 5 years.^61^ We will also estimate HCV incidence from our transmission dynamic models.^24 54^

### Sample size, power and estimating intervention effect

**Figure 3:**
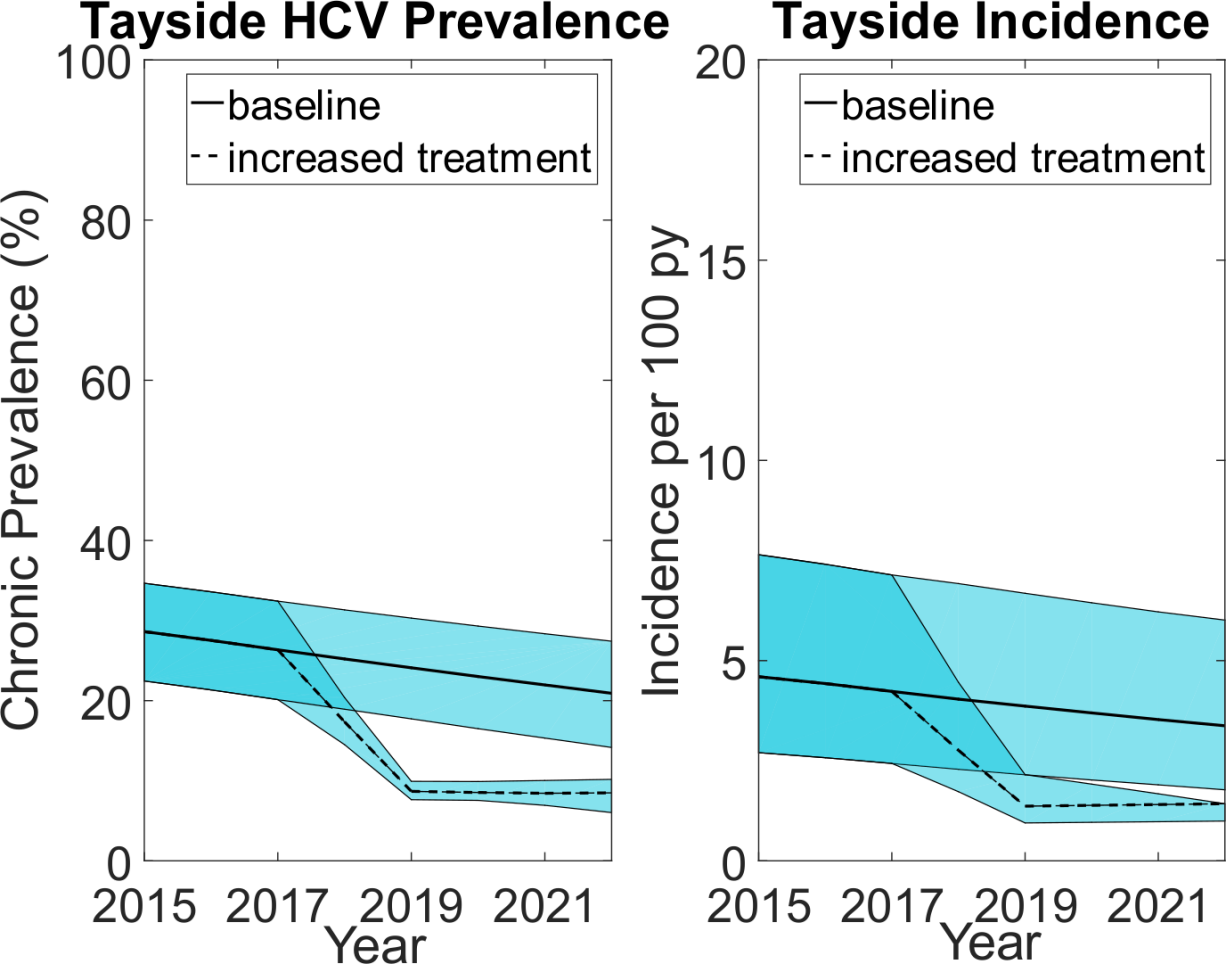
Projected chronic HCV prevalence and incidence among PWID in Tayside with and without the intervention. Blue shaded area denotes the 95% credibility intervals of the model projections with and without the intervention. HCV, hepatitisC virus; PWID, people who inject drugs; py, person-years.

We updated estimates of the prevalence of PWID in Tayside^5^ which suggest there are 2760 (95% Credible Interval, CrI 2360 to 3170) PWID either currently injecting and/or in OST. NESI data suggest that approximately 30% have chronic HCV and over 75% of PWID with chronic HCV have been diagnosed. Prior to 2017 approximately 66 PWID were treated annually. From April 2017 we plan to treat at least 500 PWID in Tayside over 2 years (as a result of expanded community care pathways shown in figure 1 and extra HCV treatments provided by NHS, Scottish Government and Industry funding). Adapting a transmission dynamic model that has been used in Tayside,^87^ we hypothesise that within 2 years chronic HCV prevalence among PWID will reduce by approximately 62% from 26% (95% CrI 20 to 32) to at least 10% and chronic HCV incidence will fall by approximately 2/3 s from 4.2 (95% CrI 2.4 to 7.1) per 100 person-years (p100py) to 1.4 (95%CrI 1.0 to 1.4) p100py (as shown in figure 3). Modelling also suggests that maintaining these reductions after 2019 will require less than 40 treatments per year.

We will adapt the Causal Impact synthetic control Model (CIM) as proposed by Brodersen and colleagues.^88 89^

**Figure 4:**
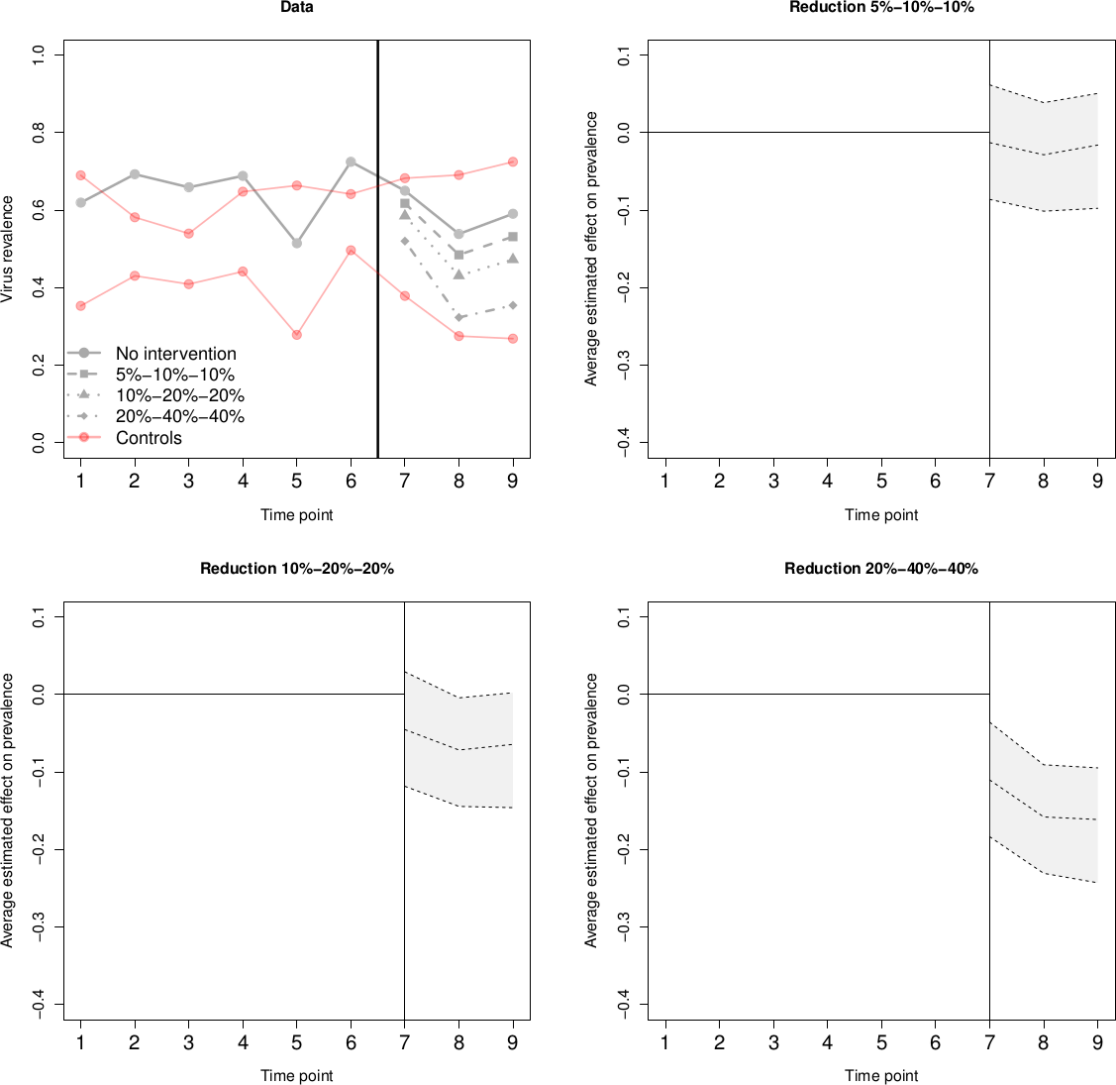
Causal impact synthetic control method (CIM) simulation and estimated intervention effects and 95% credible intervals for a range of assumed effects. Footnote: Illustration of CIM. First subplot shows a single data set, where solid lines represent the simulated prevalence in the absence of the intervention, and the dashed lines represent the outcome of treated site in the post-intervention period under different intervention magnitude scenarios. For each one of the three scenarios, we calculate the estimated average intervention effect along with credible intervals. These are shown in subplots 2 to 4. We see that as the effect increases, the intervals tend to move away for zero. However, the intervention effect only becomes significant in scenario 3, where zero is not included in any of the post-intervention time points.

We have performed simulation studies to test power and evaluate the utility of the CIM assuming information on chronic HCV prevalence among PWID (shown in figure 4). Provided trends in the chronic HCV prevalence in the pre-intervention period are relatively stable (which is the case) there will be sufficient power to detect the projected reduction in chronic prevalence. For example, in figure 4d we see that for a prevalence reduction of 40% by year 2 to 3 the credible intervals of the estimated cumulative effect (cumulative drop in prevalence) exclude zero, correctly identifying evidence of a successful intervention. Whereas a cumulative reduction of <20% is unlikely to be detected.

### Qualitative studies

#### Understanding the barriers and facilitators to scaling-up community-based HCV treatment

The qualitative study design has two distinct arms focusing on the intervention providers, and the intervention recipients.

#### Intervention providers

A purposive sample of 30 intervention providers, comprising nursing leads and key individuals from collaborating organisations will be approached directly by the lead hepatitis nurse. Seven focus groups will be convened according to professional role and locality:

- HCV healthcare specialists (nurses and physicians).
- Community pharmacists.
- Prison staff (both healthcare and security).
- ‘Drug workers’ (from OST and NSP services).

Each focus group will consist of a maximum of six individuals and ideally comprise multi-agency mixed groups. Individual interviews by telephone will be offered for those hesitant to join a group (estimate 10 interviews). Topic guides informed by previous work in this area^66 68 76 90^ will facilitate group discussion.

#### Intervention recipients – cross-sectional and longitudinal

The intervention recipient arm of the study will comprise both cross-sectional and longitudinal elements. A cross-sectional approach will be employed to recruit 6 to 10 participants who do not take up the offer of treatment. These individuals will be recruited through the treatment pathways or through our peer-researcher networks. The longitudinal element will follow a cohort of up to 40 individuals recruited following their course of HCV treatment. These individuals will be purposively sampled from the existing services in which HCV TasP has been embedded (ie, pharmacy, prison and drug service), and then followed-up at 1 year post-treatment (with 70% expected to be followed-up).^91^ We aim to recruit women as well as men, younger and older people; those treated previously and first time; those injecting and not injecting at treatment onset. Follow-up interviews will explore collateral effects of HCV TasP including outcomes pertaining to drug use and injecting practices (secondary outcome below).

Participants will be recruited by hepatitis nurses or other clinical staff in Tayside and the face-to-face semi-structured interview will be conducted by peer-researchers, trained and guided by experienced qualitative researchers. Dr Magdalena Harris explains the importance of the use of peer researchers within the context of EPIToPe: https://www.youtube.com/watch?v=9ZZo3fKOXlg.^92 93^ The Scottish Drugs Forum (SDF) works with a group of Tayside peer-researchers with lived experience of injecting. Peer-researchers will receive study-orientated training and be provided with ongoing support to co-produce data and contribute to study outputs. A £20 shopping voucher will be offered to all interviewees except those in prison (Scottish prison service ethics did not permit thank you vouchers to prison participants).

#### Qualitative data analysis

Interviews and focus-groups will be audio-recorded using encrypted digital voice recorders, transcribed verbatim and anonymised. *Nvivo* v.10 software will be used to code and manage qualitative data. First level analysis will be deductive, guided by the research questions, and peer researchers will be consulted for input and feedback during the analytical process.^94^ A constant comparison method will be used to develop the thematic analysis and will reflect diverging and converging narratives, for example, across groups of intervention recipients at different time points in the treatment pathway, or between groups of intervention providers.^94^ The findings will be contextualised in the relevant theoretical perspectives which may include the diffusion of preventive innovations (staff) or social norms and values that might underpin health behaviour (recipients).^95 96^ We will assess TasP both from the providers’ perspective and from patients’ perspective including those who refuse treatment.

**Figure 5:**
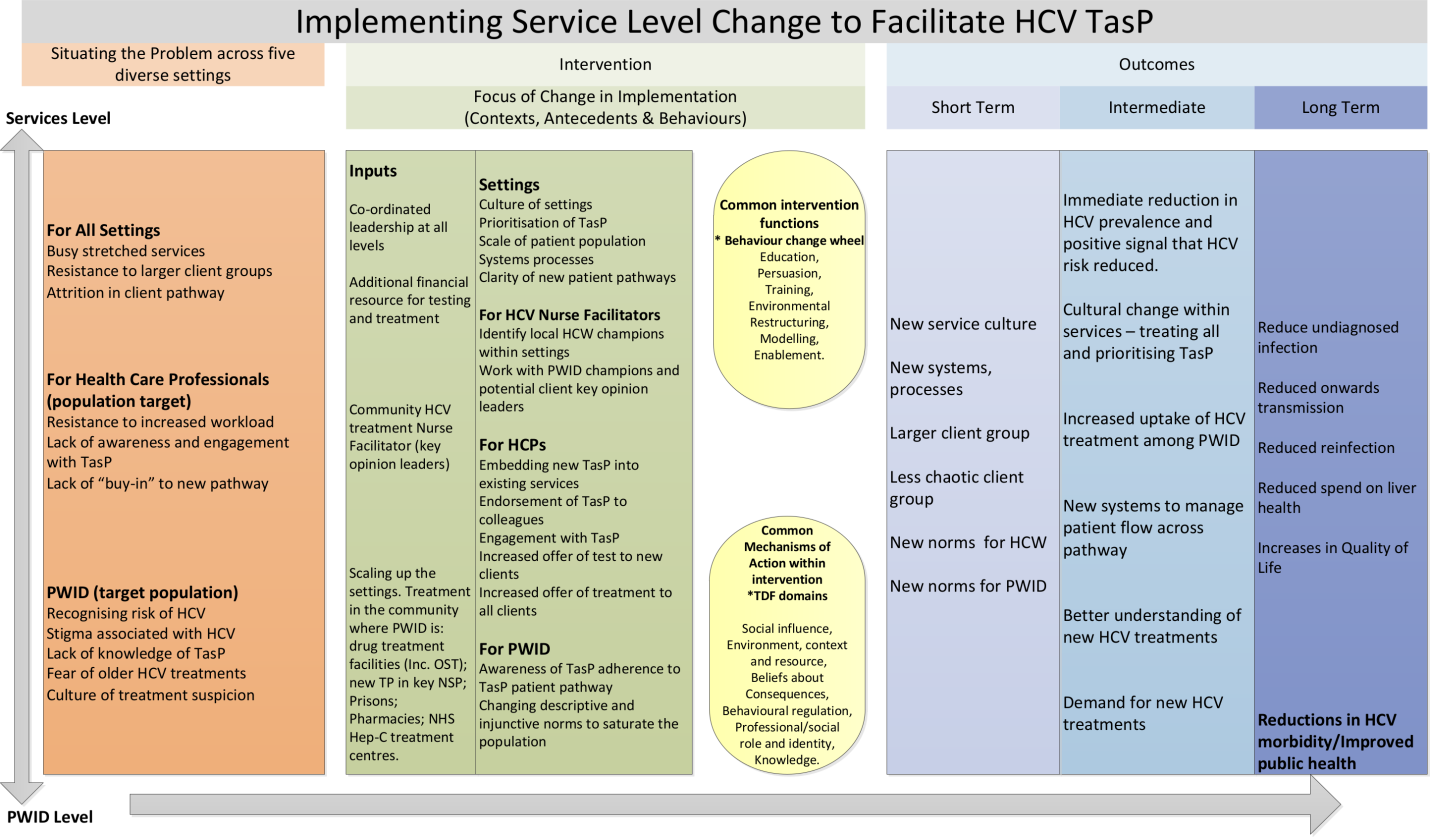
Preliminary logic model HCV treatment as prevention (EPIToPe). HCPs, healthcare providers; HCV, hepatitis C virus; HCWs, healthcare workers; Hep-C, hepatitis C; NHS, National Health Service; NSP, needle and syringe programmes; TasP, Treatment as Prevention; PWID, people who inject drugs.

We will use the findings iteratively to update the HCV TasP logic model shown in figure 5. Our qualitative data will be used to generate a manual of an optimal intervention for other sites in UK. In previous examples, such as (https://www.youtube.com/channel/UCBV8smLmkOQVT9D0OR-md1g/videos) we have used the Behaviour Change Wheel^96^ as the framework to retrospectively analyse the success and failure of implementation within Tayside and then prospectively to formulate the optimal implementation intervention.

### Mixed method study on drug use outcomes: OST retention, drug overdose, recovery and social transformation

Health Protection Scotland (HPS) link data on diagnostic HCV tests in the four largest Scottish NHS boards (including Tayside)^8^ and all persons undergoing HCV treatment in the Scottish HCV Clinical database^97^ which are also linked with other databases (including deaths, hospitalisations and drug treatment)^8 42 98–100^ and from 2018 Scotland's Prescribing Information System which holds data on OST and NHS prison health database (Prison Vision).^101–105^ PWID attending drug services who were HCV diagnosed, compared with those who were not, are at increased risk of drug-related and other cause-specific morbidity/mortality.^106 107^ Thus, we will create a virtual cohort of chronic HCV infected PWID (estimated to involve at least 600 individuals from Tayside and 3000 from elsewhere) and through linkage identify those who have been treated and attained SVR with those who have not. We will assess and compare the following outcomes: retention in drug treatment (determined through linkage to drug treatment and prescribing databases), drug-related and alcohol-related morbidity/mortality (through linkage to all hospital admission and mortality databases) and other markers of relapse (through linkage to prisons database).

#### Economic and impact evaluation

Infectious disease models can test the extent to which observed changes in disease transmission can be attributed to specific interventions,^108–112^ and assess cost-effectiveness of interventions that avert secondary infections, that is, have a population prevention benefit.^50 85 113–117^ We will update and adapt a transmission model of HCV among PWID in Scotland and Tayside to model the impact of the HCV treatment intervention based on historical trends and new observations collected as part of this programme.^39 87^ We will stratify the PWID population into current (injected in the previous year) and temporarily ceased (in OST and not injected in the previous year); as well as by duration of injecting (<3 years, 3 to 9 years, 10+years since onset), prevention intervention exposure (OST and/or high coverage NSP), and intervention settings for testing and treatment. We will use Approximate Bayesian Computation to calibrate the model to pre-intervention trends in chronic HCV prevalence and incidence among PWID in Tayside. The model will simulate the impact of observed rates of HCV treatment and cure rates for the intervention period, also incorporating any changes in the coverage of OST and NSP and injecting risk behaviours.

We will test consistency between the model impact projections and observed changes in HCV chronic prevalence and incidence from Tayside to disentangle the impact of HCV TasP from other interventions (OST/NSP) or epidemiological changes, and predict the impact of the TasP on number of HCV infections averted. If they are not consistent then alternative evidence-based hypotheses will be tested for why the model projects a different impact and the best fitting models will then be used to project the impact of the intervention. This will be assessed compared with two alternative counterfactuals where treatment rates are either at pre-scale-up levels in Tayside or at the average level achieved in other UK sites over the scale-up period. The impact of any changes in OST and NSP coverage will also be assessed to determine the contribution of those changes on observed effects. Impact will be assessed in terms of the relative decrease in prevalence and incidence, as well as the number and per cent of infections averted in the intervention model projections compared with each counterfactual over different time frames. These model projections can also be taken forward to evaluate the possible impact of the intervention over next 5 or 10 years.

We will evaluate the cost-effectiveness of the intervention (HCV treatment scale-up) compared with status quo (expected rate of HCV case-finding and treatment among PWID in the rest of the UK) from a healthcare provider (NHS) perspective, with the cost-effectiveness of the different settings where case-finding occurs also being assessed. The cost-effectiveness model will be based on the same dynamic impact model, adapted to include HCV disease progression stages and tracking of health outcomes among PWID after cessation of injecting.^50^ The economic evaluation will incorporate both individual benefits of HCV treatment (on disease progression) as well as population benefits (on HCV transmission). We will calculate the total number of infections and deaths over a 50 year time horizon for the intervention and counterfactual scenario and estimate the costs and quality-adjusted life years (QALYs) based on the number of individuals in each disease stage per year in the model. We will discount all future costs and QALYs at 3.5% (The National Institute for Health and Care Excellence (NICE) guidelines https://www.nice.org.uk/process/pmg9/resources/guide-to-the-methods-of-technology-appraisal-2013-pdf-2007975843781). Probabilistic sensitivity analyses will be used to estimate the parametric uncertainty in the impact and cost projections. Cost-effectiveness results will be expressed in terms of incremental cost-effectiveness ratios and net monetary benefits estimated using NICE thresholds (£20 000 and £30 000 per QALY). We will plot cost-effectiveness acceptability curves to determine the probability of the intervention being cost-effective compared with different willingness-to-pay thresholds. Analyses of covariance methods will be used to summarise the proportion of the variability in the incremental costs and QALYs explained by uncertainty in different input parameters. Univariate sensitivity analyses will consider the effect of changes in important parameters such as time horizon, treatment cost and discount rate.

We focus on the incremental or additional resource costs associated with the intervention in Tayside. These costs, in part based on our earlier work for other studies, will include such things as the nurse time spent on intervention related activities (training other staff to offer HCV testing and treatment referral) as well as additional HCV testing and treatment costs, any additional OST costs due to HCV testing or treatment, and other staff time at the NSP, drug treatment centres and prisons involved with the intervention. Most of the incremental costs can be defined as variable (driven by extra nurse time and HCV testing/treatment costs). NHS HCV care costs and health utilities will be attached to each disease stage, based primarily on previous syntheses and models, which assume that PWID have a lower quality of life (QoL) than non-PWID of a similar age, gender and liver disease stage.^118–120^ Additional data using the EQ-5D-5L tool during this study will generate new health utility data on the QoL among PWID before and after DAA treatment.

### Patient and public involvement

Patient and public involvement (PPI) was led by the Hepatitis C Trust and supported by qualitative research assessing barriers and facilitators to HCV treatment access (led by Dr Magdalena Harris). The SDF were also actively involved in the development of EPIToPe. The input from PPI groups has influenced the design of care pathways and has ensured that peer research is an essential element of the qualitative strand of EPIToPe.

A pilot National Institute for Health Research (NIHR) funded study in England (HEPCAT) responding to NICE Guidance on Hepatitis Case Finding was co-designed with Hepatitis C trust. It showed that Hepatitis C Facilitators and peer-support networks can increase the uptake of HCV case-finding and HCV treatment readiness in addiction services. This pilot study and our studies in Dundee/Tayside will influence how HCV treatment can be scaled up in England and our proposed evaluation HCV treatment as prevention.

Peer researchers will be trained to conduct the longitudinal study with PWID treated for HCV and will be involved and contribute to the analysis of the findings. Peer researchers and SDF will be members of the project management group and steering committee.

Dissemination events will be held in Dundee to discuss and present the findings from the qualitative studies with patient groups and services. These will be facilitated by SDF to support active contribution from our peer researchers. The study findings will be summarised and promoted through SDF website, social media platforms and through their sector-wide conferences in Scotland. Hepatitis Scotland, who are hosted within SDF, together with patient and public groups in England will take an active role in the wider national and international dissemination of the research, it’s translation into patient meaningful materials and its integration into a national policy context. The research will also be promoted via Hepatitis C Trust and Public Health England.

## Discussion

### Strengths and limitations of this study

Several limitations arise from the ‘natural experiment’ design as our intervention and controls were not randomised. In the UK and many other countries there is no longer sufficient equipoise in clinicians and policymakers – given WHO and national strategies on HCV ‘elimination’ – to mount an randomised controlled trial of HCV TasP. As a result, there will be confounding and additional uncertainty in the measurement of the intervention effect. However, we consider that a natural experiment and use of synthetic control methods to be a more robust design than simple before and after studies. Our preliminary simulation work also suggests that we should have sufficient power to detect the large intervention effect that is planned.

We know also that HCV treatment and prevention strategy in UK (and Europe) is evolving – motivated both by WHO ‘elimination targets’ and falling drug prices – and our control sites in Scotland and England may increase HCV treatment rates earlier than expected. This will complicate the analyses a little and potentially dilute the intervention effect. We are confident that we can adapt the synthetic control methods to take account of changes over time – and that because Tayside has started so early in scaling up HCV treatment that we will have time to detect a difference in the outcome.

The lack of randomised controls means that we have to generate the counterfactual of ‘no HCV treatment scale-up’ through our HCV transmission model so that we can subsequently estimate cost-effectiveness of the intervention in Tayside. This is not ideal but has become standard practice in economic models of novel HCV treatment interventions – and we are involved with the modelling of HCV treatment pathways through homeless centres, prison, Accident and Emergency (A&E), pharmacies, specialist drug clinics and NSPs (P Vickerman personal communication and for example^55 85 121^). We know also, however, that the benefit in terms of additional Quality of Life Years and averted HCV infections accrues and occurs over a prolonged period.^50^ It is more critical for any economic evaluation of HCV interventions in PWID that a dynamic model is used so that the prevention benefit (in terms of HCV infections averted) is correctly accounted for.

We are using peer researchers in the qualitative arm of patients’ perspectives on the intervention and on the impact of HCV treatment on addiction outcomes. This is novel but adds additional challenges to obtaining NHS passports and ensuring data quality across the interviews and interviewees. We are also intending to support peers in analysis and interpretation of the findings which we believe has not been done before. We have trained the interviewers and will be monitoring their performance of the interviewers to ensure consistent study quality – and will replace peers with our qualitative researcher if required.

### Future study: natural experiment of TasP in England

In England HCV treatment is delivered through 22 operational delivery networks (ODNs). NHS England’s HCV strategy (2016 to 2019) prioritised 10 000 patients per year in line with the declared priorities of the network which could (and in many cases did) include people who use drugs at risk of transmission.^44^ In October 2018 it is anticipated that a new procurement deal will substantially increase the number of patients who can access DAAs and this will enable ‘trace and treat’ options to be introduced. We will use the first part of EPIToPe including the manual generated by the qualitative study, enhancements to historical and ongoing surveillance of chronic HCV in PWID, infectious disease models and methodological developments of causal impact model, to co-design with ODN leads a natural experiment of HCV TasP in England.

## Supplementary Material

Reviewer comments

Author's manuscript

## References

[1] Williams R, Aspinall R, Bellis M, et al Addressing liver disease in the UK: a blueprint for attaining excellence in health care and reducing premature mortality from lifestyle issues of excess consumption of alcohol, obesity, and viral hepatitis. Lancet 2014;384:1953–97. 10.1016/S0140-6736(14)61838-9 25433429

[2] De Angelis D, Sweeting M, Ades AE, et al An evidence synthesis approach to estimating hepatitis C prevalence in England and Wales. Stat Methods Med Res 2009;18:361–79. 10.1177/0962280208094691 19036917

[3] Harris RJ, Ramsay M, Hope VD, et al Hepatitis C prevalence in England remains low and varies by ethnicity: an updated evidence synthesis. Eur J Public Health 2012;22:187–92. 10.1093/eurpub/ckr083 21708792

[4] Hutchinson SJ, Roy KM, Wadd S, et al Hepatitis C virus infection in Scotland: epidemiological review and public health challenges. Scott Med J 2006;51:8–15. 10.1258/RSMSMJ.51.2.8 16722130

[5] Prevost TC, Presanis AM, Taylor A, et al Estimating the number of people with hepatitis C virus who have ever injected drugs and have yet to be diagnosed: an evidence synthesis approach for Scotland. Addiction 2015;110:1287–300. 10.1111/add.12948 25876667 PMC4744705

[6] Hutchinson SJ, McIntyre PG, Molyneaux P, et al Prevalence of hepatitis C among injectors in Scotland 1989–2000: declining trends among young injectors halt in the late 1990s. Epidemiol Infect 2002;128:473–7. 10.1017/S0950268802006945 12113492 PMC2869844

[7] Martin NK, Foster GR, Vilar J, et al Hcv treatment rates and sustained viral response among people who inject drugs in seven UK sites: real world results and modelling of treatment impact. J Viral Hepat 2015;22:399–408. 10.1111/jvh.12338 25288193 PMC4409099

[8] McLeod A, Weir A, Aitken C, et al Rise in testing and diagnosis associated with Scotland's action plan on hepatitis C and introduction of dried blood spot testing. J Epidemiol Community Health 2014;68:1182–8. 10.1136/jech-2014-204451 25170094

[9] Palmateer NE, Hutchinson SJ, Innes H, et al Review and meta-analysis of the association between self-reported sharing of needles/syringes and hepatitis C virus prevalence and incidence among people who inject drugs in Europe. International Journal of Drug Policy 2013;24:85–100. 10.1016/j.drugpo.2012.08.006 23026541

[10] Palmateer NE, Taylor A, Goldberg DJ, et al Rapid decline in HCV incidence among people who inject drugs associated with national scale-up in coverage of a combination of harm reduction interventions. PLoS One 2014;9:e104515 10.1371/journal.pone.0104515 25110927 PMC4128763

[11] Roy KM, Hutchinson SJ, Wadd S, et al Hepatitis C virus infection among injecting drug users in Scotland: a review of prevalence and incidence data and the methods used to generate them. Epidemiol Infect 2007;135:433–42. 10.1017/S0950268806007035 16893486 PMC2870592

[12] Turner KME, Hutchinson S, Vickerman P, et al The impact of needle and syringe provision and opiate substitution therapy on the incidence of hepatitis C virus in injecting drug users: pooling of UK evidence. Addiction 2011;106:1978–88. 10.1111/j.1360-0443.2011.03515.x 21615585

[13] Harris RJ, Hope VD, Morongiu A, et al Spatial mapping of hepatitis C prevalence in recent injecting drug users in contact with services. Epidemiol Infect 2011:1–10. 10.1017/S0950268811001634 21875456

[14] Hickman M, Hope V, Brady T, et al Hepatitis C virus (HCV) prevalence, and injecting risk behaviour in multiple sites in England in 2004. J Viral Hepat 2007;14:645–52. 10.1111/j.1365-2893.2007.00855.x 17697017

[15] Hope VD, Hickman M, Ngui SL, et al Measuring the incidence, prevalence and genetic relatedness of hepatitis C infections among a community recruited sample of injecting drug users, using dried blood spots. J Viral Hepat 2011;18:262–70. 10.1111/j.1365-2893.2010.01297.x 20456636

[16] Sutton AJ, Gay NJ, Edmunds WJ, et al Modelling the force of infection for hepatitis B and hepatitis C in injecting drug users in England and Wales. BMC Infect Dis 2006;6 10.1186/1471-2334-6-93 PMC1524784 16762050

[17] Sweeting MJ, Hope VD, Hickman M, et al Hepatitis C infection among injecting drug users in England and Wales (1992-2006): there and back again? Am J Epidemiol 2009;170:352–60. 10.1093/aje/kwp141 19546152 PMC2714950

[18] Wiessing L, Ferri M, Grady B, et al Hepatitis C virus infection epidemiology among people who inject drugs in Europe: a systematic review of data for scaling up treatment and prevention. PLoS One 2014;9:e103345 10.1371/journal.pone.0103345 25068274 PMC4113410

[19] Hickman MM, editor N Hepatitis C among drug users in Europe: epidemiology, treatment and prevention. Lisbon: EMCDDA, 2016.

[20] Van Den Berg C, Smit C, Van Brussel G, et al Full participation in harm reduction programmes is associated with decreased risk for human immunodeficiency virus and hepatitis C virus: evidence from the Amsterdam cohort studies among drug users. Addiction 2007;102:1454–62. 10.1111/j.1360-0443.2007.01912.x 17697278 PMC2040242

[21] Hagan H, Pouget ER, Des Jarlais DC A systematic review and meta-analysis of interventions to prevent hepatitis C virus infection in people who inject drugs. Journal of Infectious Diseases 2011;204:74–83. 10.1093/infdis/jir196 21628661 PMC3105033

[22] Platt L, Reed J, Minozzi S, et al Effectiveness of needle/syringe programmes and opiate substitution therapy in preventing HCV transmission among people who inject drugs. Cochrane Database Syst Rev 2016 2016;(1). 10.1002/14651858.CD012021 PMC4843520 27127417

[23] Hutchinson SJ, Dillon JF, Fox R, et al Expansion of HCV treatment access to people who have injected drugs through effective translation of research into public health policy: Scotland's experience. Int J Drug Policy 2015. 10.1016/j.drugpo.2015.05.019 26123893

[24] Fraser H, Mukandavire C, Martin NK, et al Modelling the impact of a national scale-up of interventions on hepatitis C virus transmission among people who inject drugs in Scotland. Addiction 2018;113:2118–31. 10.1111/add.14267 29781207 PMC6250951

[25] de Vos AS, van der Helm JJ, Matser A, et al Decline in incidence of HIV and hepatitis C virus infection among injecting drug users in Amsterdam; evidence for harm reduction? Addiction 2013;108:1070–81. 10.1111/add.12125 23347124

[26] Vickerman P, Martin N, Turner K, et al Can needle and syringe programmes and opiate substitution therapy achieve substantial reductions in hepatitis C virus prevalence? model projections for different epidemic settings. Addiction 2012;107:1984–95. 10.1111/j.1360-0443.2012.03932.x 22564041

[27] Dore GJ, Feld JJ Hepatitis C virus therapeutic development: in pursuit of "perfectovir". Clinical Infectious Diseases 2015;60:1829–36. 10.1093/cid/civ197 25761867

[28] Gogela NA, Lin MV, Wisocky JL, et al Enhancing our understanding of current therapies for hepatitis C virus (HCV). Curr HIV/AIDS Rep 2015;12:68–78. 10.1007/s11904-014-0243-7 25761432 PMC4373591

[29] Walker DR, Pedrosa MC, Manthena SR, et al Early view of the effectiveness of new direct-acting antiviral (DAA) regimens in patients with hepatitis C virus (HCV). Adv Ther 2015;32:1117–27. 10.1007/s12325-015-0258-5 26538232 PMC4662718

[30] Hellard M, Doyle JS, Sacks-Davis R, et al Eradication of hepatitis C infection: the importance of targeting people who inject drugs. Hepatology 2014;59:366–9. 10.1002/hep.26623 23873507 PMC4298812

[31] Hagan LM, Wolpe PR, Schinazi RF Treatment as prevention and cure towards global eradication of hepatitis C virus. Trends Microbiol 2013;21:625–33. 10.1016/j.tim.2013.09.008 24238778 PMC7700888

[32] Grebely J, Matthews GV, Lloyd AR, et al Elimination of hepatitis C virus infection among people who inject drugs through treatment as prevention: feasibility and future requirements. Clinical Infectious Diseases 2013;57:1014–20. 10.1093/cid/cit377 23728143

[33] Bruggmann P Treatment as prevention: the breaking of taboos is required in the fight against hepatitis C among people who inject drugs. Hepatology 2013;58:1523–5. 10.1002/hep.26539 23728921

[34] Martin NK, Vickerman P, Grebely J, et al Hepatitis C virus treatment for prevention among people who inject drugs: modeling treatment scale-up in the age of direct-acting antivirals. Hepatology 2013;58:1598–609. 10.1002/hep.26431 23553643 PMC3933734

[35] Wedemeyer H, Duberg AS, Buti M, et al Strategies to manage hepatitis C virus (HCV) disease burden. J Viral Hepat 2014;21(Suppl. 1):60–89. 10.1111/jvh.12249 24713006

[36] Martin NK, Vickerman P, Dore GJ, et al The hepatitis C virus epidemics in key populations (including people who inject drugs, prisoners and MSM): the use of direct-acting antivirals as treatment for prevention. Current opinion in HIV and AIDS 2015;10:374–80. 26248124 10.1097/COH.0000000000000179 PMC4659815

[37] Hickman M, De Angelis D, Vickerman P, et al Hcv treatment as prevention in people who inject Drugs–testing the evidence. Current opinion in infectious diseases 2015;28:576. 26524330 10.1097/QCO.0000000000000216 PMC4659818

[38] Hayes R, Ayles H, Beyers N, et al HPTN 071 (PopART): rationale and design of a cluster-randomised trial of the population impact of an HIV combination prevention intervention including universal testing and treatment – a study protocol for a cluster randomised trial. Trials 2014;15:57 10.1186/1745-6215-15-57 24524229 PMC3929317

[39] Fraser H, Zibbell J, Hoerger T, et al Scaling-up HCV prevention and treatment interventions in rural United States-model projections for tackling an increasing epidemic. Addiction 2018;113:173–82. 10.1111/add.13948 28734093 PMC6211174

[40] Organization WH Global health sector strategy on viral hepatitis 2016-2021: towards ending viral hepatitis. Geneva: WHO, 2016.

[41] Innes HA, McDonald SA, Dillon JF, et al Toward a more complete understanding of the association between a hepatitis C sustained viral response and cause-specific outcomes. Hepatology 2015;62:355–64. 10.1002/hep.27766 25716707

[42] Innes HA, Hutchinson SJ, Allen S, et al Excess liver-related morbidity of chronic hepatitis C patients, who achieve a sustained viral response, and are discharged from care. Hepatology 2011;54:1547–58. 10.1002/hep.24561 22045672

[43] Innes H, Goldberg D, Dillon J, et al Strategies for the treatment of hepatitis C in an era of interferon-free therapies: what public health outcomes do we value most? Gut 2015;64:1800–9. 10.1136/gutjnl-2014-308166 25378522

[44] Harris RJ, Martin NK, Rand E, et al New treatments for hepatitis C virus (HCV): scope for preventing liver disease and HCV transmission in England. J Viral Hepat 2016. 10.1111/jvh.12529 PMC4982023 27025238

[45] European Association for the Study of the Liver EASL recommendations on treatment of hepatitis C 2014. J Hepatol 2014;61:373–95. 10.1016/j.jhep.2014.05.001 24818984

[46] European Association for Study of Liver EASL recommendations on treatment of hepatitis C 2015. J Hepatol 2015;63:199–236. 10.1016/j.jhep.2015.03.025 25911336

[47] Panel A Hepatitis C guidance: AASLD-IDSA recommendations for testing, managing, and treating adults infected with hepatitis C virus. Hepatology 2015. 10.1002/hep.27950 26111063

[48] Grebely J, Robaeys G, Bruggmann P, et al Recommendations for the management of hepatitis C virus infection among people who inject drugs. International Journal of Drug Policy 2015;26:1028–38. 10.1016/j.drugpo.2015.07.005 26282715 PMC6130980

[49] European Association for the Study of the Liver Electronic address eee. EASL recommendations on treatment of hepatitis C 2016. J Hepatol 2016. 10.1016/j.jhep.2022.10.006 36464532

[50] Martin NK, Vickerman P, Dore GJ, et al Prioritization of HCV treatment in the direct-acting antiviral era: an economic evaluation. J Hepatol 2016;65:17–25. 10.1016/j.jhep.2016.02.007 26867489 PMC4914770

[51] Aleman S, Rahbin N, Weiland O, et al A risk for hepatocellular carcinoma persists long-term after sustained virologic response in patients with hepatitis C-associated liver cirrhosis. Clin Infect Dis 2013;57:230–6. 10.1093/cid/cit234 23616492

[52] van der Meer AJ, Veldt BJ, Feld JJ, et al Association between sustained virological response and all-cause mortality among patients with chronic hepatitis C and advanced hepatic fibrosis. JAMA 2012;308:2584–93. 10.1001/jama.2012.144878 23268517

[53] Innes H, Hutchinson SJ, Obel N, et al Liver mortality attributable to chronic hepatitis C virus infection in Denmark and Scotland-Using spontaneous resolvers as the benchmark comparator. Hepatology 2016;63:1506–16. 10.1002/hep.28458 26773546

[54] Fraser H, Martin NK, Brummer-Korvenkontio H, et al Model projections on the impact of HCV treatment in the prevention of HCV transmission among people who inject drugs in Europe. J Hepatol 2018;68:402–11. 10.1016/j.jhep.2017.10.010 29080808 PMC5841161

[55] Schulkind J, Stephens B, Ahmad F, et al High response and re-infection rates among people who inject drugs treated for hepatitis C in a community needle and syringe programme. J Viral Hepat 2019;26:519–28. 10.1111/jvh.13035 30422370

[56] MacArthur GJ, Minozzi S, Martin N, et al Opiate substitution treatment and HIV transmission in people who inject drugs: systematic review and meta-analysis. BMJ 2012;345:e5945 10.1136/bmj.e5945 23038795 PMC3489107

[57] MacArthur GJ, van VE, Palmateer N, et al Interventions to prevent HIV and hepatitis C in people who inject drugs: a review of reviews to assess evidence of effectiveness. IntJ Drug Policy 2013. 10.1016/j.drugpo.2013.07.001 23973009

[58] Palmateer N, Hutchinson S, McAllister G, et al Risk of transmission associated with sharing drug injecting paraphernalia: analysis of recent hepatitis C virus (HCV) infection using cross-sectional survey data. J Viral Hepat 2014;21:25–32. 10.1111/jvh.12117 24329854

[59] Palmateer N, Kimber J, Hickman M, et al Evidence for the effectiveness of sterile injecting equipment provision in preventing hepatitis C and human immunodeficiency virus transmission among injecting drug users: a review of reviews. Addiction 2010;105:844–59. 10.1111/j.1360-0443.2009.02888.x 20219055

[60] Presanis AM, Gill ON, Chadborn TR, et al Insights into the rise in HIV infections, 2001 to 2008: a Bayesian synthesis of prevalence evidence. AIDS 2010;24:2849–58. 10.1097/QAD.0b013e32834021ed 20962617

[61] Harris HE Hepatitis C in the UK 2016 report: working towards its elimination as a major public health threat. London: Public Health England, 2016.

[62] Health Protection Scotland UotWoS, Glasgow Caledonian University and the West of Scotland Specialist Virology Centre The needle exchange surveillance initiative: prevalence of blood-borne viruses and injecting risk behaviours among people who inject drugs attending injecting equipment provision services in Scotland, 2008-09 to 2015-16. Scotland HP: Glasgow, 2017.

[63] Brant LJ, Hurrelle M, Balogun MA, et al Sentinel laboratory surveillance of hepatitis C antibody testing in England: understanding the epidemiology of HCV infection. Epidemiol Infect 2007;135:417–26. 10.1017/S0950268806006832 16836798 PMC2870581

[64] Brant LJ, Hurrelle M, Balogun MA, et al Where are people being tested for anti-HCV in England? results from sentinel laboratory surveillance. J Viral Hepat 2008;15:729–39. 10.1111/j.1365-2893.2008.01000.x 18637078

[65] Lattimore S, Irving W, Collins S, et al Using surveillance data to determine treatment rates and outcomes for patients with chronic hepatitis C virus infection. Hepatology 2014;59:1343–50. 10.1002/hep.26926 24214920 PMC4258076

[66] Treloar C, Newland J, Rance J, et al Uptake and delivery of hepatitis C treatment in opiate substitution treatment: perceptions of clients and health professionals. J Viral Hepat 2010;17:839–44. 10.1111/j.1365-2893.2009.01250.x 20070504

[67] Treloar C, Rance J, Dore GJ, et al Barriers and facilitators for assessment and treatment of hepatitis C virus infection in the opioid substitution treatment setting: insights from the ethos study. J Viral Hepat 2014;21:560–7. 10.1111/jvh.12183 24299222

[68] Harris M, Rhodes T Hepatitis C treatment access and uptake for people who inject drugs: a review mapping the role of social factors. Harm Reduct J 2013;10:7 10.1186/1477-7517-10-7 23651646 PMC3686576

[69] Swan D, Long J, Carr O, et al Barriers to and facilitators of hepatitis C testing, management, and treatment among current and former injecting drug users: a qualitative exploration. AIDS Patient Care STDS 2010;24:753–62. 10.1089/apc.2010.0142 21138381

[70] Rhodes T, Harris M, Martin A Negotiating access to medical treatment and the making of patient citizenship: the case of hepatitis C treatment. Sociol Health Illn 2013;35:1023–44. 10.1111/1467-9566.12018 23701040

[71] Harris M, Albers E, Swan T The promise of treatment as prevention for hepatitis C: meeting the needs of people who inject drugs? International Journal of Drug Policy 2015;26:963–9. 10.1016/j.drugpo.2015.05.005 26143385

[72] Rance J, Treloar C, Group ES, ETHOS Study Group 'Not just methadone Tracy': transformations in service-user identity following the introduction of hepatitis C treatment into Australian opiate substitution settings. Addiction 2014;109:452–9. 10.1111/add.12392 24678545

[73] Treloar C, Rance J, Backmund M Understanding barriers to hepatitis C virus care and stigmatization from a social perspective. Clin Infect Dis 2013;57(suppl_2):S51–S55. 10.1093/cid/cit263 23884066

[74] Treloar C, Rance J, Bryant J, et al Harm reduction workers and the challenge of engaging couples who inject drugs in hepatitis C prevention. Drug Alcohol Depend 2016;168:170–5. 10.1016/j.drugalcdep.2016.09.010 27665209

[75] Treloar C, Rance J, Bryant J, et al Understanding decisions made about hepatitis C treatment by couples who inject drugs. J Viral Hepat 2016;23:89–95. 10.1111/jvh.12451 26305873

[76] Jack K, Islip N, Linsley P, et al Prison officers' views about hepatitis C testing and treatment: a qualitative enquiry. J Clin Nurs 2016. 10.1111/jocn.13489 27486922

[77] Rich ZC, Chu C, Mao J, et al Facilitators of HCV treatment adherence among people who inject drugs: a systematic qualitative review and implications for scale up of direct acting antivirals. BMC Public Health 2016;16:994 10.1186/s12889-016-3671-z 27645935 PMC5029046

[78] Clark JA, Gifford AL Resolute efforts to cure hepatitis C: Understanding patients’ reasons for completing antiviral treatment. Health 2015;19:473–89. 10.1177/1363459314555237 25377666

[79] Batchelder AW, Peyser D, Nahvi S, et al “Hepatitis C treatment turned me around:” Psychological and behavioral transformation related to hepatitis C treatment. Drug Alcohol Depend 2015;153:66–71. 10.1016/j.drugalcdep.2015.06.007 26096534 PMC4759650

[80] Harris M Managing expense and expectation in a treatment revolution: Problematizing prioritisation through an exploration of hepatitis C treatment ‘benefit’. International Journal of Drug Policy 2017;47:161–8. 10.1016/j.drugpo.2017.03.015 28455145

[81] Aspinall EJ, Hutchinson SJ, Janjua NZ, et al Trends in mortality after diagnosis of hepatitis C virus infection: an international comparison and implications for monitoring the population impact of treatment. J Hepatol 2015;62:269–77. 10.1016/j.jhep.2014.09.001 25200903

[82] Aspinall EJ, Corson S, Doyle JS, et al Treatment of hepatitis C virus infection among people who are actively injecting drugs: a systematic review and meta-analysis. Clin Infect Dis 2013;57(suppl_2):S80–S89. 10.1093/cid/cit306 23884071

[83] Judd A, Parry J, Hickman M, et al Evaluation of a modified commercial assay in detecting antibody to hepatitis C virus in oral fluids and dried blood spots. J Med Virol 2003;71:49–55. 10.1002/jmv.10463 12858408

[84] Bennett S, Gunson RN, McAllister GE, et al Detection of hepatitis C virus RNA in dried blood spots. Journal of Clinical Virology 2012;54:106–9. 10.1016/j.jcv.2012.02.004 22418454

[85] Martin NK, Vickerman P, Brew IF, et al Is increased hepatitis C virus case-finding combined with current or 8-week to 12-week direct-acting antiviral therapy cost-effective in UK prisons? A prevention benefit analysis. Hepatology 2016;63:1796–808. 10.1002/hep.28497 26864802 PMC4920048

[86] McDonald SA, Innes HA, Hayes PC, et al What is the impact of a country-wide scale-up in antiviral therapy on the characteristics and sustained viral response rates of patients treated for hepatitis C? J Hepatol 2015;62:262–8. 10.1016/j.jhep.2014.08.046 25195556

[87] Ward Z, Platt L, Sweeney S, et al Impact of current and scaled-up levels of hepatitis C prevention and treatment interventions for people who inject drugs in three UK settings-what is required to achieve the who's HCV elimination targets? Addiction 2018;113:1727–38. 10.1111/add.14217 PMC6175066 29774607

[88] Brodersen KH, Gallusser F, Koehler J, et al Inferring causal impact using Bayesian structural time-series models. Ann Appl Stat 2015;9:247–74. 10.1214/14-AOAS788

[89] Scott SL, Varian HR Predicting the present with Bayesian structural time series. IJMMNO 2014;5 10.1504/IJMMNO.2014.059942

[90] Rance J, Newland J, Hopwood M, et al The politics of place(ment): Problematising the provision of hepatitis C treatment within opiate substitution clinics. Soc Sci Med 2012;74:245–53. 10.1016/j.socscimed.2011.10.003 22133583

[91] Lankenau SE, Sanders B, Hathazi D, et al Recruiting and retaining mobile young injection drug users in a longitudinal study. Subst Use Misuse 2010;45:684–99. 10.3109/10826081003594914 20222779 PMC3139269

[92] Stewart KE, Wright PB, Sims D, et al The “Translators”: Engaging Former Drug Users as Key Research Staff to Design and Implement a Risk Reduction Program for Rural Cocaine Users. Subst Use Misuse 2012;47:547–54. 10.3109/10826084.2011.644379 22428822 PMC3571708

[93] Harris M "Three in the Room": Embodiment, Disclosure, and Vulnerability in Qualitative Research. Qual Health Res 2015;25:1689–99. 10.1177/1049732314566324 25576481

[94] Braun V, Clarke V Using thematic analysis in psychology. Qual Res Psychol 2006;3:77–101. 10.1191/1478088706qp063oa

[95] Rogers EM Diffusion of preventive innovations. Addict Behav 2002;27:989–93. 10.1016/S0306-4603(02)00300-3 12369480

[96] Cane J, O’Connor D, Michie S Validation of the theoretical domains framework for use in behaviour change and implementation research. Implementation Sci 2012;7 10.1186/1748-5908-7-37 PMC3483008 22530986

[97] Innes HA, Hutchinson SJ, Allen S, et al Ranking predictors of a sustained viral response for patients with chronic hepatitis C treated with pegylated interferon and ribavirin in Scotland. Eur J Gastroenterol Hepatol 2012;24:646–55. 10.1097/MEG.0b013e32835201a4 22433796

[98] McDonald SA, Hutchinson SJ, Bird SM, et al Excess morbidity in the hepatitis C-diagnosed population in Scotland, 1991-2006. Epidemiol Infect 2011;139:344–53. 10.1017/S0950268810001421 20587118

[99] McDonald SA, Hutchinson SJ, Bird SM, et al A record-linkage study of the development of hepatocellular carcinoma in persons with hepatitis C infection in Scotland. Br J Cancer 2008;99:805–10. 10.1038/sj.bjc.6604563 18728670 PMC2528155

[100] McDonald SA, Hutchinson SJ, Bird SM, et al A population-based record linkage study of mortality in hepatitis C-diagnosed persons with or without HIV coinfection in Scotland. Stat Methods Med Res 2009;18:271–83. 10.1177/0962280208094690 19036907

[101] Gao L, Dimitropoulou P, Robertson JR, et al Risk-factors for methadone-specific deaths in Scotland’s methadone-prescription clients between 2009 and 2013*. Drug Alcohol Depend 2016;167:214–23. 10.1016/j.drugalcdep.2016.08.627 27593969 PMC5047032

[102] Bird SM, Fischbacher CM, Graham L, et al Impact of opioid substitution therapy for Scotland's prisoners on drug-related deaths soon after prisoner release. Addiction 2015;110:1617–24. 10.1111/add.12969 25940815 PMC4744745

[103] Bird SM, Hutchinson SJ Male drugs-related deaths in the fortnight after release from prison: Scotland, 1996-99. Addiction 2003;98:185–90. 10.1046/j.1360-0443.2003.00264.x 12534423

[104] Alvarez-Madrazo S, McTaggart S, Nangle C, et al Data resource profile: the Scottish national prescribing information system (PIs). Int J Epidemiol 2016;45:714–5. 10.1093/ije/dyw060 27165758 PMC5005947

[105] Graham L, Fischbacher CM, Stockton D, et al Understanding extreme mortality among prisoners: a national cohort study in Scotland using data linkage. Eur J Public Health 2015;25:879–85. 10.1093/eurpub/cku252 25678604

[106] Merrall ELC, Bird SM, Hutchinson SJ Mortality of those who attended drug services in Scotland 1996–2006: record-linkage study. International Journal of Drug Policy 2012;23:24–32. 10.1016/j.drugpo.2011.05.010 21719267 PMC3271367

[107] McDonald SA, Hutchinson SJ, Bird SM, et al Hospitalisation for an alcohol-related cause among injecting drug users in Scotland: increased risk following diagnosis with hepatitis C infection. International Journal of Drug Policy 2011;22:63–9. 10.1016/j.drugpo.2010.04.003 20472416

[108] de Vos AS, Prins M, Kretzschmar MEE Hepatitis C virus treatment as prevention among injecting drug users: who should we cure first? Addiction 2015;110:975–83. 10.1111/add.12842 25586171

[109] Boily M-C, Lowndes CM, Vickerman P, et al Evaluating large-scale HIV prevention interventions: study design for an integrated mathematical modelling approach. Sex Transm Infect 2007;83:582–9. 10.1136/sti.2007.027516 17942574 PMC2598645

[110] Boily M-C, Pickles M, Lowndes CM, et al Positive impact of a large-scale HIV prevention programme among female sex workers and clients in South India. AIDS 2013;27:1449–60. 10.1097/QAD.0b013e32835fba81 23462221 PMC3678895

[111] Hallett TB, Gregson S, Mugurungi O, et al Assessing evidence for behaviour change affecting the course of HIV epidemics: a new mathematical modelling approach and application to data from Zimbabwe. Epidemics 2009;1:108–17. 10.1016/j.epidem.2009.03.001 21352758

[112] Pickles M, Boily M-C, Vickerman P, et al Assessment of the population-level effectiveness of the Avahan HIV-prevention programme in South India: a preplanned, causal-pathway-based modelling analysis. The Lancet Global Health 2013;1:e289–99. 10.1016/S2214-109X(13)70083-4 25104493

[113] Martin NK, Hickman M, Miners A, et al Cost-Effectiveness of HCV case-finding for people who inject drugs via dried blood spot testing in specialist addiction services and prisons. BMJ Open 2013;3:e003153 10.1136/bmjopen-2013-003153 PMC3752052 23943776

[114] Martin NK, Miners A, Vickerman P Assessing the cost-effectiveness of interventions aimed at promoting and offering hepatitis C testing to injecting drug users: an economic modelling report. London, 2012.

[115] Martin NK, Pitcher AB, Vickerman P, et al Optimal control of hepatitis C antiviral treatment programme delivery for prevention amongst a population of injecting drug users. PLoS One 2011;6:e22309 10.1371/journal.pone.0022309 21853030 PMC3154900

[116] Martin NK, Vickerman P, Miners A, et al Cost-Effectiveness of hepatitis C virus antiviral treatment for injection drug user populations. Hepatology 2012;55:49–57. 10.1002/hep.24656 21898506

[117] Martin NK, Vickerman P, Miners A, et al How cost-effective is hepatitis C virus treatment for people who inject drugs? J Gastroenterol Hepatol 2013;28:590–2. 10.1111/jgh.12113 23527756

[118] Kimber J, Copeland L, Hickman M, et al Survival and cessation in injecting drug users: prospective observational study of outcomes and effect of opiate substitution treatment. BMJ 2010;341:c3172 10.1136/bmj.c3172 20595255 PMC2895695

[119] McDonald SA, Hutchinson SJ, Palmateer NE, et al Decrease in health-related quality of life associated with awareness of hepatitis C virus infection among people who inject drugs in Scotland. J Hepatol 2013;58:460–6. 10.1016/j.jhep.2012.11.004 23149064

[120] Wright M, Grieve R, Roberts J, et al Health benefits of antiviral therapy for mild chronic hepatitis C: randomised controlled trial and economic evaluation. Health Technol Assess 2006;10:1–113. 10.3310/hta10210 16750059

[121] Harrison GI, Murray K, Gore R, et al The hepatitis C awareness through to treatment (HepCATT) study: improving the cascade of care for hepatitis C virus‐infected people who inject drugs in England. Addiction 2019;114:1113–22. 10.1111/add.14569 30694582

