# Supplementary material for: Evaluating the population impact of hepatitis C direct acting antiviral treatment as prevention for people who inject drugs (EPIToPe) – a natural experiment (protocol)
Source: BMJ Open. 2019 Sep 24;9(9):e029538. doi: 10.1136/bmjopen-2019-029538 (PMC6773339; doi:10.1136/bmjopen-2019-029538)
Supplement: Author's manuscript [file bmjopen-2019-029538.draft_revisions.pdf]

BMJ Open is committed to open peer review. As part of this commitment we make the peer review history of every article we publish publicly available.

When an article is published we post the peer reviewers' comments and the authors' responses online. We also post the versions of the paper that were used during peer review. These are the versions that the peer review comments apply to.

The versions of the paper that follow are the versions that were submitted during the peer review process. They are not the versions of record or the final published versions. They should not be cited or distributed as the published version of this manuscript.

BMJ Open is an open access journal and the full, final, typeset and author-corrected version of record of the manuscript is available on our site with no access controls, subscription charges or pay-per-view fees (http://bmjopen.bmj.com).

If you have any questions on BMJ Open's open peer review process please email info.bmjopen@bmj.com

BMJ Open

Evaluating the Population Impact of Hepatitis C Direct Acting Antiviral Treatment as Prevention for People Who Inject Drugs (EPIToPe) – a natural experiment

Journal:	*BMJ Open*
Manuscript ID	bmjopen-2019-029538
Article Type:	Protocol
Date Submitted by the Author:	14-Feb-2019
Complete List of Authors:	Hickman, M; Bristol<br>Dillon, John; University of Dundee Division of Cardiovascular and Diabetes Medicine, Liver Group<br>Elliott, Lawrie; Glasgow Caledonian University, Department of Nursing and Community Health<br>De Angelis, Daniela; MRC Biostatistics Unit<br>Vickerman, Peter; London School of Hygiene and Tropical Medicine; University of Bristol, School of Social and Community Medicine<br>Foster, Graham; Barts and The London School of Medicine<br>Donnan, Peter; University of Dundee, Dundee Epidemiology and Biostatistics Unit<br>Eriksen, Ann; Tayside Health Board<br>Flowers, Paul; Glasgow Caledonian University,<br>Goldberg, David; Health Protection Scotland<br>Hollingworth, William; University of Bristol, School of Social and Community Medicine<br>Ijaz, Samreen; Public Health England Centre for Infection<br>Liddell, David; Scottish Drugs Forum<br>Mandal, Sema; Public Health England, Immunisation, Hepatitis, Blood Safety and Countermeasures Response; National Institute for Health Research Health Protection Unit<br>Martin, Natasha; University of California, San Diego<br>Beer, Lewis; University of Dundee, Tayside Clinical Trials Unit<br>Drysdale, Kate; Barts Health NHS Trust<br>Fraser, Hannah; University of Bristol, School of Social and Community Medicine<br>Glass, Rachel; Public Health England, Immunisation, Hepatitis, Blood Safety and Countermeasures Response<br>Graham, Lesley; ISD Scotland<br>Gunson, Rory; Gartnavel General Hospital, West Of Scotland Specialist Virology Centre<br>Hamilton, Emma; Scottish Drug Forum<br>Harris, Helen; Public Health England, Immunisation, Hepatitis, Blood Safety and Countermeasures Response<br>Harris, Magdalena; London School of Hygiene and Tropical Medicine, Centre for Research on Drugs and Health Behaviours<br>Harris, Ross; Public Health England, Immunisation, Hepatitis, Blood Safety and Countermeasures Response<br>Heinsbroek, Ellen; Public Health England Colindale

Hope, Vivian; Liverpool John Moores University
Horwood, Jeremy; University of Bristol, UK, School of Social and Community Medicine
Inglis, Sarah; University of Dundee, Division of Neuoscience; University of Dundee, Tayside Clinical Trials Unit
Innes, Hamish; Glasgow Caledonian University, School of health and life sciences
Lane, Athene; University of Bristol, School of Social and Community Medicine
Meadows, Jade; University of Bristol School of Social and Community Medicine
McAuley, Andrew; NHS Health Scotland
Metcalfe, Chris; University of Bristol, Social Medicine
Migchelsen, Stephanie; Public Health England, Immunisation, Hepatitis, Blood Safety and Countermeasures Response
Murray, Alex; Scottish Drug Forum
Myring, Gareth; University of Bristol School of Social and Community Medicine
Palmateer, Norah E.; Hlth Protect Scotland, Blood-borne viruses and sexually transmitted infections; Glasgow Caledonian University,
Presanis, Anne; Institute of Public Health, MRC Biostatistics Unit
Ramsay, Mary; Public Health England, immunisation; London School of Hygiene and Tropical Medicine, Epidemiology
Samartsidis, Pantelis; MRC Biostatistics Unit
Simmons, Ruth; Public Health England,
Sinka, Katy; Public Health England, Immunisation, Hepatitis, Blood Safety and Countermeasures Response
Vojt, Gabriele; Glasgow Caledonian University
Ward, Zoe; Bristol, Population Health Sciences, Bristol Medical School
Whiteley, David; Edinburgh Napier University
Yeung, Alan; Glasgow Caledonian University
Hutchinson, Sharon; Health Protection Scotland, ; Glasgow Caledonian University, School of Health and Life Sciences

Keywords: Hepatology < INTERNAL MEDICINE, INFECTIOUS DISEASES, Epidemiology < INFECTIOUS DISEASES, PUBLIC HEALTH, Public health < INFECTIOUS DISEASES, Infection control < INFECTIOUS DISEASES

SCHOLARONE™
Manuscripts

ISRCTN72038467

Evaluating the **P**opulation **I**mpact of Hepatitis C Direct Acting Antiviral **T**reatment as Prevention f**o**r **Pe**ople Who Inject Drugs (EPIToPe) – a natural experiment

Authors:

Hickman M[1], Dillon J[2], Elliott L[3], De Angelis D[4], Vickerman P[1], Foster G[5], Donnan PT[2], Eriksen A[6], Flowers P[3], Goldberg D[7], Hollingworth W[1], Ijaz S[8], Liddell D[9], Mandal S[8], Martin NK[10,1], Beer L[2] , Drysdale K[5], Fraser H[1], Glass R[8], Graham L[11], Gunson R[12], Hamilton E[9], Harris H[8], Harris M[13], Harris R[8], Heinsbroek E[8], Hope V[14], Horwood J[1], Inglis S[2], Innes H[3], Lane A[1], Meadows, J[1], McAuley A[7], Metcalfe C[1], Migchelsen S[8], Murray, A[9], G Myring[1] , Palmateer N[3], Presanis A[4], Ramsay M[8], Samartsidis P[4], Simmons R[8], Sinka K[8], Vojt, G[3] , Ward Z[1], Whiteley D[15], Yeung, A[3] , Hutchinson S[3]

Correspondence to: Prof Matthew Hickman; Matthew.Hickman@bristol.ac.uk

Affiliations

1 Population Health Sciences, Bristol Medical School, University of Bristol

2 University of Dundee

3 Glasgow Caledonian University

4 MRC Biostatistics Unit, University of Cambridge

5 Barts Liver Centre, Queen Mary University of London

6 Tayside Health Board

7 Health Protection Scotland

8 Public Health England

9 Scottish Drug Forum

10 Division of Infectious Diseases and Global Public Health, University of California San Diego

11 Information Services Division, Scotland

12 West of Scotland Specialist Virology Centre, Glasgow Royal Infirmary

13 London School of Hygiene and Tropical Medicine

14 Liverpool John Moores University

15 Edinburgh Napier University

For peer review only

For peer review only - http://bmjopen.bmj.com/site/about/guidelines.xhtml

ISRCTN72038467

Shorter ABSTRACT

Introduction

Modelling evidence suggests HCV treatment scale-up can substantially reduce HCV prevalence/incidence among PWID. We aim to generate empirical evidence on the effectiveness of HCV "Treatment as Prevention" (TasP) in people who inject drugs (PWID).

Methods and Analysis

We plan a natural experiment with Tayside, Scotland, as a single intervention site where HCV care pathways are being expanded (including drug treatment clinics, needle & syringe programmes, pharmacies, and prison). Other sites in Scotland and England will act as potential controls. Over two years from 2017/18 500 PWID will be treated in Tayside, which we project will reduce chronic HCV prevalence by 62% (from 26% to 10%) and HCV incidence will fall from 4.2 to 1.4 per 100 person-years.

We will conduct focus groups and interviews with service providers and patients to identify barriers and facilitators in implementing TasP; and conduct longitudinal interviews with 40 PWID to assess whether successful HCV treatment alters perspectives on and engagement with drug treatment and recovery. These qualitative accounts will be compared to outcomes generated from a "virtual cohort" of PWID linking information on HCV treatment with Scottish Drug treatment databases. Trained peer researchers will be involved in data collection and dissemination.

The primary outcome – chronic HCV prevalence in PWID – is measured by the Needle Exchange Surveillance Initiative (NESI) survey in Scotland and the Unlinked Anonymous Monitoring Programme (UAM) in England, conducted at least four times before and three times during and after the intervention. We will adapt Bayesian synthetic control methods to generate the cumulative impact of the intervention on chronic HCV prevalence and incidence. We will use a dynamic HCV transmission and economic model to evaluate the cost-effectiveness of the HCV TasP intervention, and to estimate the contribution of the scale-up in HCV treatment to observed changes in HCV prevalence.

For peer review only

ISRCTN72038467

Longer ABSTRACT

Introduction:

Hepatitis C Virus (HCV) is the second most important cause of liver disease in the UK, with injecting drug use the main risk factor among the estimated 200,000 people currently infected. Despite effective prevention interventions, chronic HCV prevalence remains at 40% among people who inject drugs (PWID). New Direct Acting Antiviral (DAA) HCV therapies combine high cure rates (>90%) and short treatment duration (8-12 weeks). Theoretical mathematical modelling evidence suggests HCV treatment scale-up can prevent transmission and substantially reduce HCV prevalence/incidence among PWID. Our primary aim is to generate empirical evidence on the effectiveness of HCV "Treatment as Prevention" (TasP) in PWID.

Methods and Analysis

We plan to establish a natural experiment with Tayside, Scotland, as a single intervention site where HCV care pathways are being expanded (including specialist drug treatment clinics, needle & syringe programmes (NSPs), pharmacies, and prison) and HCV treatment for PWID is being rapidly scaled-up. Other sites in Scotland and England will act as potential controls. Over two years from 2017/18, 500 PWID will be treated in Tayside, which simulation studies project will reduce chronic HCV prevalence among PWID by 62% (from 26% to 10%) and HCV incidence will fall by approximately 2/3 (from 4.2 per 100 person-years (p100py) to 1.4 p100py). Treatment response and re-infection rates will be monitored. We will conduct focus groups and interviews with service providers and patients that accept and decline treatment to identify barriers and facilitators in implementing TasP. We will conduct longitudinal interviews with up to 40 PWID to assess whether successful HCV treatment alters their perspectives on and engagement with drug treatment and recovery. Trained peer researchers will be involved in data collection and dissemination.

The primary outcome – chronic HCV prevalence in PWID – is measured using information from the Needle Exchange Surveillance Initiative (NESI) survey in Scotland and the Unlinked Anonymous Monitoring Programme (UAM) in England, conducted at least four times before and three times during and after the intervention. We will adapt Bayesian synthetic control methods (also called Causal Inference Models) to generate the cumulative impact of the intervention on chronic HCV prevalence and incidence. We will use a dynamic HCV transmission and economic model to evaluate the cost-effectiveness of the HCV TasP intervention, and to estimate the contribution of the scale up in HCV treatment to observed changes in HCV prevalence. Through the qualitative data we will systematically explore key mechanisms of TasP real world implementation from provider and patient perspectives, to develop a manual for scaling up HCV treatment in other settings. We will compare qualitative accounts of drug treatment and recovery with a "virtual cohort" of PWID linking information on HCV treatment with Scottish Drug treatment databases to test whether DAA treatment improves drug treatment outcomes.

Ethics and Dissemination

Extending HCV community care pathways is covered by ethics (ERADICATE C, ISRCTN27564683, Super DOT C Trial clinicaltrials.gov:NCT02706223). Ethical approval for extra data collection from patients including health utilities and qualitative interviews has been granted and ISCRCTN registration has been completed. Our findings will have direct NHS and patient relevance; informing

For peer review only - http://bmjopen.bmj.com/site/about/guidelines.xhtml

ISRCTN72038467

prioritization given to early HCV treatment for PWID. We will present findings to practitioners and policy makers, and support design of an evaluation of HCV TasP in England.

Strengths and limitations of this study

1. Our control sites in the rest of Scotland and England were not randomised - but our natural experiment design adapted from synthetic control methods is more robust than simple before and after studies.
2. HCV treatment and prevention strategy in UK (and Europe) is evolving - motivated both by WHO “elimination targets” and falling drug prices – which may contaminate our controls.
3. Our statistical models suggest that we should have sufficient power to detect an intervention effect and can model changes over time.
4. We will develop dynamic transmission and economic models that can estimate cost-effectiveness including the prevention benefit of this intervention.
5. We are conducting multiple nested qualitative studies and training and using peer researchers.

For peer review only

For peer review only - http://bmjopen.bmj.com/site/about/guidelines.xhtml

ISRCTN72038467

Introduction:

Infection with Hepatitis C Virus (HCV) is a progressive disease that over 20-40 years can lead to liver cancer and premature death. HCV is the second most important cause of liver disease in the UK and one of the few causes that is curable[1]. In the UK it is estimated that approximately 200,000 people are infected with HCV, over 85% of whom are people who inject or have injected drugs (PWID)[2-5]. Chronic HCV prevalence and incidence among PWID remains high in UK at 20-50% and 5 to 15 per 100 person- years respectively[4 6-18]. Prevention of HCV transmission among PWID is critical to long-term prevention of HCV related liver disease[19].

We have reviewed the effectiveness of traditional primary prevention against HCV –opioid substitution treatment (OST) and needle and syringe programmes (NSPs)[12 20-22]. Ongoing exposure to OST and high-coverage NSPs can reduce the risk of HCV transmission by 50-80%[12 22]. In Scotland HCV incidence among PWID decreased from approximately 14 to 6 per 100 person- years from 2008/09 to 2011/12 coinciding with the launch of the Scottish HCV strategy and action plan which incorporated scale-up of harm reduction interventions and HCV treatment[10 23]. We estimated that 60% of this decline could be attributed to the scale-up of OST and NSP during the action plan and that 1,400 HCV infections were averted by 2015[24]. However, there was no appreciable reduction in overall anti-HCV prevalence over this short period, and there is some suggestion that incidence has increased recently to ~10 per 100 person years (http://www.hps.scot.nhs.uk/resourcedocument.aspx?id=5863). HCV transmission models suggest that primary prevention through NSP and OST alone is insufficient to achieve substantial reductions (of the order of 40% or more within ten years) in HCV prevalence among PWID in the UK[25 26].

Prevention of hepatitis C disease and HCV transmission is now possible because highly effective, tolerable, short-course interferon-free direct acting antiviral therapies (DAAs) are available for all HCV genotypes with cure rates – defined as sustained virological response (SVR)- exceeding 90%[27-29]. We, and others, hypothesise that HCV Treatment scale-up for PWID, and resulting HCV Treatment as Prevention (TasP) could enhance other primary interventions and reduce HCV incidence and chronic prevalence to negligible levels (i.e. towards elimination as a major public health concern)[30-35]. World Health Organization (WHO) targets for HCV elimination, adopted by UK and other countries, aim to reduce HCV incidence by 80% and associated mortality by 65% by 2030[36 37 38 39 40 41].

Clinical guidelines in Europe and US changed from recommending prioritising HCV treatment to people with moderate to severe liver disease towards removing any restrictions and recommending that people at risk of transmission irrespective of fibrosis stage are offered treatment[42-46]. Cost-effectiveness models that incorporate the population prevention benefit suggest early treatment should be prioritised to PWID over other patient groups (unless chronic HCV prevalence and transmission is very high)[47]. There is direct evidence that SVR following HCV treatment reduces liver disease progression and mortality risk[48-50], but in two recent reviews we found no empirical evidence that HCV treatment scale-up has reduced chronic HCV prevalence and incidence in PWID populations[51 52]. In part this is because in most settings HCV treatment rates in PWID are too low and any changes generally too small to be detected, as we show in two studies of seven sites in UK[7] and an extension to 11 sites in Europe[53]. Until very recently in the UK, the annual number of HCV DAA treatments was restricted -as drug costs could be expensive (>£10,000 per patient). There is

For peer review only - http://bmjopen.bmj.com/site/about/guidelines.xhtml

ISRCTN72038467

the opportunity now to test whether scaling up HCV treatment will reduce chronic HCV prevalence and transmission among PWID[41].

In a pilot study ("Eradicate C") in Tayside we showed that we can increase HCV case-finding and engage and successfully treat PWID in the community (Dillon personal communication, Schulkind under review). Combining further studies on extending community HCV treatment pathways in Tayside and additional treatments provided by NHS Tayside and Scottish Government we can establish an immediate natural experiment (with Tayside as the intervention site and other sites in Scotland and England as controls) to test and generate UK empirical evidence on the and potential impact and cost-effectiveness of HCV "Treatment as Prevention" (TasP) in people who inject drugs (PWID).

For peer review only

ISRCTN72038467

Methods and analysis:

Our intention is to create and conduct a mixed methods study, including qualitative studies and economic evaluation, of a natural experiment of HCV Treatment as Prevention (TasP) among People who Inject Drugs (PWID). We also will develop methods for evaluating HCV TasP.

Intervention: Scaling-up HCV treatment

The **intervention** comprises the removal of any restrictions on access to treatment by disease stage, expanding opportunities for HCV treatment through multiple community care-pathways, and scaling-up treatment in PWID. By combining support from Scottish Government, National Health Board Tayside (NHS Tayside) and industry (MSD, Gilead, BMS) we can deliver rapid intensive scale-up of HCV treatments for PWID (comprising an extra 400 HCV treatments, a 3.5-fold increase from treatments for PWID prior to April 2017, see sample size below). We have developed multiple integrated community HCV care pathways, including novel care pathways in pharmacies, a low threshold NSP, drug treatment services and prisons (see Figure 1). Our diagnostic pathways make extensive use of dried blood spot (DBS) testing for diagnosis of HCV antibody and chronic HCV with subsequent conventional laboratory testing in preparation for treatment (viral load, liver function and Fib4 fibrosis score)[54-56]. Community HCV specialist nurses (3.5 FTE will coordinate and deliver case-finding and treatment across the pathways in Tayside (Figure 1).

Figure 1: Overview of HCV testing and treatment pathways for the PWID population in NHS Tayside.

Apart from expansion of community HCV care pathways, no new clinical procedures will be investigated and all PWID with chronic HCV will be offered oral DAA HCV treatment compliant with the Scottish clinical guidelines (https://www.hps.scot.nhs.uk/resourcedocument.aspx?id=6621

). Standard care for patients is to test for SVR at 12 weeks after end of treatment with patients being recommended for annual follow-up if at risk of re-infection. Additionally, ethical approval has been granted to ask patients for permission to be recruited into the qualitative study (below) and extended clinical and behavioural drug history and data on health utilities (EQ5D-5L) at onset of treatment, during treatment and after the end of treatment.

Outcome – Chronic HCV prevalence in PWID

The **outcome** is chronic HCV prevalence among PWID in the community (not just in the patients who undergo HCV treatment).

The UK is one of few countries worldwide to have an established nationwide surveillance system monitoring HCV infection among PWID [9 12 17 22 57-61]. This is undertaken through a series of cross-sectional voluntary anonymous surveys of PWID recruited at harm reduction services, referred to as the Unlinked Anonymous Monitoring Programme (UAM) in England and Wales and the Needle Exchange Surveillance Initiative (NESI) in Scotland[62 63]. In addition, the UK has established sentinel laboratory surveillance of HCV testing and national monitoring of HCV treatment [8 64-66].

In our pre-intervention period from 2010/11 to 2016 there have been four NESI surveys in Scotland (n=10,000 participants in total) and six UAM surveys in England (n=16,000 in total), which have involved the collection of DBS linked to questionnaire data. Participants are recruited at sentinel sites by a team of trained interviewers in Scotland (at over 100 NSP sites) and by agency staff in over

For peer review only - http://bmjopen.bmj.com/site/about/guidelines.xhtml

ISRCTN72038467

60 low-threshold drug agencies across England[59 62]. Participants complete a short questionnaire, with common questions across UAM and NESI, on demographics, injecting behaviour and service utilisation, and importantly (in relation to quantifying the intervention effect) both survey approaches have remained consistent over time.

The DBS samples collected in NESI and UAM have all been tested for HCV antibody, using the same methods (where sensitivity and specificity of the assay on DBS are close to 100%)[54 55], and illustrate that antibody prevalence (ever infection) has remained relatively stable among PWID during this time (Figure 2). PCR positivity among antibody positive samples is used to determine chronic infection. All NESI and UAM samples will be tested for HCV antibody and PCR to assess the impact of HCV therapy scale-up – which is critical as trends in chronic infection and antibody status will diverge as more people are cured. In addition, we will undertake PCR testing of all historical samples that were HCV antibody positive shown in Figure 2 so that we can measure chronic HCV prevalence among PWID pre-, as well as post-, intervention for analysis (below)

Figure 2: Trends in HCV antibody prevalence among PWID in Scotland and England 2010/11-16

Data on HCV PCR positivity among antibody negative samples identify recent infections and is used to estimate HCV incidence – which has fluctuated between 5-10 infections per 100 person years across the UK during the last five years[62].

During 2017-22, three waves of data collection for NESI (n=7,500) and five to six for UAM (n=17,000 in England) will measure this outcome.

Sample size, Power, and Estimating Intervention Effect

We updated estimates of the prevalence of PWID in Tayside[5] which suggest there are 2,760 (95% Credible Interval, CrI 2,360-3,170) PWID either currently injecting and/or in OST. We estimate that approximately 30% have chronic HCV and over 75% of PWID with chronic HCV have been diagnosed. Prior to 2017 approximately 66 PWID were treated annually. From April 2017 we plan to treat at least 500 PWID in Tayside over 2 years (as a result of expanded community care pathways shown in Figure 1 and extra HCV treatments provided by NHS, Scottish Government and Industry funding). Adapting a transmission dynamic model that has been used in Tayside,[67] we hypothesize that within two years chronic HCV prevalence among PWID will reduce by approximately 62% from 26% (95% CrI 20-32) to at least 10% and chronic HCV incidence will fall by approximately 2/3s from 4.2 (95% CrI 2.4-7.1) per 100 person-years (p100py) to 1.4 (95%CrI 1.0 – 1.4) p100py (as shown in Figure 3). Modelling also suggests that maintaining these reductions after 2019 will require less than 40 treatments per year.

Figure 3: Projected chronic HCV prevalence and incidence among PWID in Tayside with and without the intervention. Blue shaded area denotes the 95% credibility intervals of the model projections with and without the intervention

We selected synthetic control type methods as the most appropriate approach for evaluating HCV TasP intervention effects in a natural experiment – given that the outcome – chronic HCV in PWID -

For peer review only - http://bmjopen.bmj.com/site/about/guidelines.xhtml

is measured over time in the pre-intervention period and data on multiple control sites (as shown in Figure 2) that have not been exposed to the intervention are available. The original synthetic control method [68 69 70] is limited and does not fully exploit the temporal characteristics of the outcome. We will, therefore, adapt an alternative approach, the "*causal impact* method" (CIM) proposed by Brodersen and colleagues[71 72]. In the CIM a time series model is formulated of the outcome in the treated site at each time point in terms of previous outcomes and a regression component with covariates related to the outcomes in the control sites. In this way both the temporal correlation of the outcome within the intervention site and with control units are accounted for. The model is estimated in a Bayesian framework using data from the pre-intervention period. The estimated parameters are then used, together with information on outcomes in the control units after the intervention, to forecast the outcome for the treated site post-intervention, i.e. chronic HCV prevalence in the absence of the intervention with the intervention effect as the cumulative difference between the observed outcome and the counterfactual. In our case, this would correspond to the difference in chronic HCV prevalence among PWID between the synthetic control estimated prevalence and the observed prevalence after the intervention.

We have performed simulation studies to test power and evaluate the utility of the CIM assuming information on chronic HCV prevalence among PWID (shown in Figure 4). Provided trends in the chronic HCV prevalence in the pre-intervention period are relatively stable (which is the case) there will be sufficient power to detect the projected reduction in chronic prevalence. For example, in Figure 4d we see that for a prevalence reduction of 40% by year 2-3 the credible intervals of the estimated cumulative effect (cumulative drop in prevalence) exclude zero, correctly identifying evidence of a successful intervention. Whereas a cumulative reduction of <20% is unlikely to be detected.

Figure 4: Causal Impact Synthetic Control Method (CIM) simulation and estimated intervention effects and 95% Credible Intervals for a range of assumed effects.

Footnote:- Illustration of CIM. First subplot shows a single dataset, where solid lines represent the simulated prevalence in the absence of the intervention, and the dashed lines represent the outcome of treated site in the post intervention period under different intervention magnitude scenarios. For each one of the three scenarios, we calculate the estimated average intervention effect along with credible intervals. These are shown in Subplots 2-4. We see that as the effect increases, the intervals tend to move away for zero. However, the intervention effect only becomes significant in scenario 3, where zero is not included in any of the post-intervention time points.

Qualitative Studies:
Historically it has proven very hard to engage PWID in HCV treatment[73-76]. Some barriers to engagement, such as poor efficacy or fear of interferon treatment side-effects, may be ameliorated by DAA therapy. However, other barriers such as mistrust of health services, stigma, and competing priorities faced by PWID may persist. In addition, providers may be reticent to refer or provide HCV treatment to PWID due to concerns about adherence, reinfection and perceptions of treatment 'worth'[77 78]. It is expected that co-locating HCV treatment within existing services will reduce many system and provider level barriers to PWID accessing care[73-75 79-84]. However, this has not been tested in the context of community wide scale-up of interventions across multiple potential pathways (Figure 1). It is critical, therefore, that we understand how HCV TasP is embedded within the existing service landscape and incorporated into providers' professional roles. In addition, we will use qualitative studies to assess whether successful HCV treatment impacts on reduction or

For peer review only - http://bmjopen.bmj.com/site/about/guidelines.xhtml

cessation of drug use, safer injecting practices and improvements in social relationships[85] (secondary outcome below).

Understanding the barriers and facilitators to scaling-up community-based HCV treatment
The qualitative study design has two distinct arms focusing on the intervention providers, and the intervention recipients.

Intervention providers
A purposive sample of 30 intervention providers, comprising nursing leads and key individuals from collaborating organisations will be approached directly by the lead hepatitis nurse. Seven focus groups will be convened according to professional role and locality:

- HCV healthcare specialists (nurses and physicians)
- Community pharmacists
- Prison staff (both healthcare and security)
- 'Drug workers' (from OST and NSP services)

Each focus group will consist of a maximum of six individuals and ideally comprise multi-agency mixed groups. Individual interviews by telephone will be offered for those hesitant to join a group (estimate 10 interviews). Topic guides informed by previous work in this area[73 75 83 86] will facilitate group discussion.

Intervention recipients – cross-sectional and longitudinal
The intervention recipient arm of the study will comprise both cross-sectional and longitudinal elements. A cross-sectional approach will be employed to recruit 6-10 participants who do not take up the offer of treatment. These individuals will be recruited through the treatment pathways or through our peer researcher networks. The longitudinal element will follow a cohort of up to 40 individuals recruited following their course of HCV treatment. These individuals will be purposively sampled from the existing services in which HCV TasP has been embedded (i.e. pharmacy, prison and drug service), and then followed-up at one year post-treatment (with 70% expected to be followed-up)[87]. We aim to recruit women as well as men, younger and older people; those treated previously and first time; those injecting and not injecting at treatment onset. Follow-up interviews will explore collateral effects of HCV TasP including outcomes pertaining to drug use and injecting practices (secondary outcome below).

Participants will be recruited by hepatitis nurses or other clinical staff in Tayside and the face-to-face semi-structured interview will be conducted by peer-researchers, trained and guided by experienced qualitative researchers (https://www.youtube.com/watch?v=9ZZo3fKOXlg)

[88 89]. The Scottish Drugs Forum (SDF) works with a group of Tayside peer-researchers with lived experience of injecting. Peer-researchers will receive study-orientated training and be provided with ongoing support to co-produce data and contribute to study outputs. A £20 shopping voucher will be offered to all interviewees except those in prison (Scottish prison service ethics did not permit thank you vouchers to prison participants).

Qualitative Data Analysis
Interviews and focus-groups will be audio-recorded using encrypted digital voice recorders, transcribed verbatim and anonymised. *Nvivo* v.10 software will be used to code and manage qualitative data. First level analysis will be deductive, guided by the research questions, and peer researchers will be consulted for input and feedback during the analytical process[90]. A constant comparison method will be used to develop the thematic analysis and will reflect diverging and converging narratives, for example, across groups of intervention recipients at different time points in the treatment pathway, or between groups of intervention providers[90]. The findings will be

For peer review only - http://bmjopen.bmj.com/site/about/guidelines.xhtml

contextualised in the relevant theoretical perspectives which may include the diffusion of preventive innovations (staff) or social norms and values that might underpin health behaviour (recipients)[91 92]. We will assess TasP both from the providers' perspective and from patients' perspective including those who refuse treatment.

We will use the findings iteratively to update the HCV TasP logic model shown in Figure 5. Our qualitative data will be used to generate a manual of an optimal intervention for other sites in UK**.** The Behaviour Change Wheel [92] will be used retrospectively to analyse the success and failure of implementation within Tayside and then prospectively to formulate the optimal implementation intervention.

Figure 5: Preliminary Logic Model HCV Treatment as Prevention (EPIToPe)

Mixed Method Study on drug use outcomes: OST retention, drug overdose, recovery, and social transformation

It has been hypothesised that successful HCV treatment in PWID may positively impact on understandings of self and identity and improve treatment of drug use disorders[78 79 85 93 94]. Accounts of 'transformative' outcomes extending beyond viral clearance alone include reference to reductions in drug and alcohol use, uptake of safer injecting practices, improved social relationships, enhanced sense of responsibility and self-worth (Harris personal communication, under review). Hints of such collateral or indirect benefits are also found in quantitative studies reporting low re-infection rates and reductions in risky injecting behaviours among treated PWID[95 96]. In our qualitative follow-up study we will describe accounts of drug treatment experience, injecting risk and self-concept in PWID who have been successfully treated and compare the findings to quantitative data generated from a virtual cohort.

Health Protection Scotland (HPS) link data on diagnostic HCV tests in the four largest Scottish NHS boards (including Tayside)[8] and all persons undergoing HCV treatment in the Scottish HCV Clinical database[97] which are also linked with other databases (including deaths, hospitalisations and drug treatment)[8 39 98-100] and from 2018 Scotland's Prescribing Information System (PIS) which holds data on OST and NHS prison health database (Prison Vision)[101-105]. PWID attending drug services who were HCV diagnosed, compared to those who were not, are at increased risk of drug-related and other cause-specific morbidity/mortality[106 107]. Thus, we will create a virtual cohort of chronic HCV infected PWID (estimated to involve at least 600 individuals from Tayside and 3,000 from elsewhere) and through linkage identify those who have been treated and attained SVR with those who have not. We will assess and compare the following outcomes:- retention in drug treatment (determined through linkage to drug treatment and prescribing databases), drug- and alcohol- related morbidity/mortality (through linkage to all hospital admission and mortality databases), and other markers of relapse (through linkage to prisons database).

Economic and impact evaluation

Infectious disease models can test the extent to which observed changes in disease transmission can be attributed to specific interventions,[108-112] and assess cost-effectiveness of interventions that avert secondary infections, i.e. have a population prevention benefit [47 56 113-117]. We will update and adapt a transmission model of HCV among PWID in Scotland and Tayside to model the impact of the HCV treatment intervention based on historical trends and new observations collected as part of this programme[36 67]**.** We will stratify the PWID population into current (injected in the previous year) and temporarily ceased (in OST and not injected in the previous year); as well as by duration of

For peer review only - http://bmjopen.bmj.com/site/about/guidelines.xhtml

injecting (< 3 years, 3 to 9 years, 10+ years since onset), prevention intervention exposure (OST and/or high coverage NSP), and intervention settings for testing and treatment. We will use Approximate Bayesian Computation to calibrate the model to pre-intervention trends in chronic HCV prevalence and incidence among PWID in Tayside. The model will simulate the impact of observed rates of HCV treatment and cure rates for the intervention period, also incorporating any changes in the coverage of OST and NSP and injecting risk behaviours.

We will test consistency between the model impact projections and observed changes in HCV chronic prevalence and incidence from Tayside to disentangle the impact of HCV TasP from other interventions (OST/NSP) or epidemiological changes, and predict the impact of the TasP on number of HCV infections averted. If they are not consistent then alternative evidence-based hypotheses will be tested for why the model projects a different impact and the best fitting models will then be used to project the impact of the intervention. This will be assessed compared to two alternative counterfactuals where treatment rates are either at pre-scale-up levels in Tayside or at the average level achieved in other UK sites over the scale-up period. The impact of any changes in OST and NSP coverage will also be assessed to determine the contribution of those changes on observed effects. Impact will be assessed in terms of the relative decrease in prevalence and incidence, as well as the number and percent of infections averted in the intervention model projections compared to each counterfactual over different time frames. These model projections can also be taken forward to evaluate the possible impact of the intervention over next 5 or 10 years.

We will evaluate the cost-effectiveness of the intervention (HCV treatment scale-up) compared to status quo (expected rate of HCV case-finding and treatment among PWID in the rest of the UK) from a health care provider (NHS) perspective, with the cost-effectiveness of the different settings where case-finding occurs also being assessed. The cost-effectiveness (CE) model will be based on the same dynamic impact model, adapted to include HCV disease progression stages and tracking of health outcomes among PWID after cessation of injecting[47]. The economic evaluation will incorporate both individual benefits of HCV treatment (on disease progression) as well as population benefits (on HCV transmission). We will calculate the total number of infections and deaths over a 50-year time horizon for the intervention and counterfactual scenario and estimate the costs and quality-adjusted life years (QALYs) based on the number of individuals in each disease stage per year in the model. We will discount all future costs and QALYs at 3.5% (NICE guidelines https://www.nice.org.uk/process/pmg9/resources/guide-to-the-methods-of-technology-appraisal-2013-pdf-2007975843781). Probabilistic sensitivity analyses will be used to estimate the parametric uncertainty in the impact and cost projections. Cost-effectiveness results will be expressed in terms of incremental cost-effectiveness ratios (ICERs) and net monetary benefits (NMB) estimated using NICE thresholds (£20,000 and £30,000 per QALY). We will plot cost-effectiveness acceptability curves to determine the probability of the intervention being cost-effective compared to different willingness-to-pay thresholds. Analyses of covariance (ANCOVA) methods will be used to summarize the proportion of the variability in the incremental costs and QALYs explained by uncertainty in different input parameters. Univariate sensitivity analyses will consider the effect of changes in important parameters such as time horizon, treatment cost and discount rate.

We focus on the incremental or additional resource costs associated with the intervention in Tayside. These costs, in part based on our earlier work for other studies, will include such things as the nurse time spent on intervention related activities (training other staff to offer HCV testing and treatment referral) as well as additional HCV testing and treatment costs, any additional OST costs due to HCV testing or treatment, and other staff time at the NSP, drug treatment centres and prisons involved with the intervention. Most of the incremental costs can be defined as variable (driven by extra nurse time and HCV testing/treatment costs). NHS HCV care costs and health utilities will be attached to each disease stage, based primarily on previous syntheses and models,

For peer review only - http://bmjopen.bmj.com/site/about/guidelines.xhtml

which assume that PWID have a lower QoL than non-PWID of a similar age, gender and liver disease stage[118-120]. Additional data using the EQ-5D-5L tool during this study will generate new health utility data on the QoL amongst PWID before and after DAA treatment.

Patient and Public Involvement

Patient and Public Involvement (PPI) was led by the Hepatitis C Trust and supported by qualitative research assessing barriers and facilitators to HCV treatment access (led by Magdalena Harris). The Scottish Drug Forum (SDF) were also actively involved in the development of EPIToPe. The input from PPI groups has influenced the design of care pathways and has ensured that peer research is an essential element of the qualitative strand of EPIToPe.

A pilot NIHR funded study in England (HEPCAT) responding to NICE Guidance on Hepatitis Case Finding was co-designed with Hepatitis C trust. It showed that Hepatitis C Facilitators and peer support networks can increase the uptake of HCV case-finding and HCV treatment readiness in addiction services. This pilot study and our studies in Dundee/Tayside will influence how HCV treatment can be scaled up in England and our proposed evaluation HCV treatment as prevention.

Peer researchers will be trained to conduct the longitudinal study with PWID treated for HCV and will be involved and contribute to the analysis of the findings. Peer researchers and SDF will be members of the project management group and steering committee.

Dissemination events will be held in Dundee to discuss and present the findings from the qualitative studies with patient groups and services. These will be facilitated by SDF to support active contribution from our peer researchers. The study findings will be summarised and promoted through SDF website, social media platforms and through their sector-wide conferences in Scotland. Hepatitis Scotland, who are hosted within SDF, together with patient and public groups in England will take an active role in the wider national and international dissemination of the research, it's translation into patient meaningful materials and its integration into a national policy context. The research will also be promoted via Hepatitis C Trust and Public Health England.

Future Study: Natural experiment of TasP in England

In England HCV treatment is delivered through 22 operational delivery networks (ODNs). NHS England's HCV strategy (2016-2019) prioritised 10,000 patients per year in line with the declared priorities of the network which could (and in many cases did) include people who use drugs at risk of transmission [41]. In October 2018 it is anticipated that a new procurement deal will substantially increase the number of patients who can access DAAs and this will enable 'trace and treat' options to be introduced. We will use the first part of EPIToPe including the manual generated by the qualitative study, enhancements to historical and ongoing surveillance of chronic HCV in PWID, infectious disease models, and methodological developments of causal impact model, to co-design with ODN leads a natural experiment of HCV TasP in England.

For peer review only - http://bmjopen.bmj.com/site/about/guidelines.xhtml

ISRCTN72038467

Authors' contributions:

All authors contributed to editing of the manuscript.

M Hickman (MH) and S Hutchinson (SH) are co-PIs of EPIToPe and prepared first draft of the manuscript.

J Dillon (JD) leads intervention scale-up in Tayside in collaboration with Tayside CTU (PT Donnan (PTD), S Inglis (SI)) and A Eriksen (AE).

L Elliot (LE) leads qualitative component of EPIToPe in collaboration with Scottish Drug Forum (D Liddell (DL), R Moore (RM)) and support from M Harris (MHa) and D Whitely (DW) on qualitative research and training of peer support workers, and P Flowers (PF) on behavioural science.

D De Angelis (DA) leads synthetic control estimation and multiple parameter evidence synthesis in collaboration with P Samartsidis (PS), R Harris (RA), A Presanis (AP), and N Martin (NM).

P Vickerman (PV) leads dynamic impact and economic modelling in collaboration with NM, Z Ward (ZW), H Fraser (HF) with health economics led by W Hollingworth (WH) as part of Bristol Randomised Trial Collaboration (BRTC) with advice on trial design from J Horwood (JH), C Metcalfe (CM) and A Lane (AL).

G Foster (GF) is leading design of evaluation in England based on EPIToPe with support from BRTC and K Drysdale

SH is leading on outcome measurement in Scotland with Health Protection Scotland (D Goldburg (DG), A McAuley (AMc)), and in collaboration with L Graham (LG) from ISD, R Gunson (RG), H Innes (HI), N Palmateer (NP).

S Mandal (SM) and S Ijaz (SI) are leading on outcome measurement in England with H Harris (HH), V Hope (VH), S Migchelsen (SM), M Ramsay (MR), R Simmons (RS), K Sinka (SK).

Funding statement: 'This paper presents independent research funded by the National Institute for Health Research (NIHR) under its Programme Grants for Applied Research Programme (Reference Number RP-PG-0616-20008). The views expressed are those of the author(s) and not necessarily those of the NHS, the NIHR or the Department of Health.'

In addition, we acknowledge support from NIHR Health Protection Research Unit in Evaluation, and the Bristol Randomised Trials Collaboration (BRTC), a UKCRC Registered Clinical Trials Unit in receipt of NIHR Clinical Trials Units (CTU) support funding. The views expressed are those of the author and not necessarily those of the NHS, the NIHR or the Department of Health and Social Care. NM is supported by the National Institute for Drug Abuse [grant number R01 DA037773] and the University of California San Diego Center for AIDS Research (CFAR), a National Institute of Health (NIH) funded program [grant number P30 AI036214].

We would like to thank all those involved in PPI from both SDF and the Hepatitis C Trust.

Competing interests statement.
MH has received unrestricted honoraria for presenting at meetings from Abbvie, Gilead, MSD. NM has received unrestricted research grants and honoraria from Gilead and Merck. PV has received unrestricted honoraria for presenting at meetings from Abbvie and Gilead. PV has received unrestricted research grants from Gilead.

For peer review only - http://bmjopen.bmj.com/site/about/guidelines.xhtml

ISRCTN72038467

PTD has received unrestricted grants from Shire pharmaceuticals, Novo Nordisk and Gilead and is a member of the Scottish Medicines Consortium. HF has received an honorarium from MSD.

For peer review only

For peer review only - http://bmjopen.bmj.com/site/about/guidelines.xhtml

ISRCTN72038467

References

1. Williams R, Aspinall R, Bellis M, et al. Addressing liver disease in the UK: a blueprint for attaining excellence in health care and reducing premature mortality from lifestyle issues of excess consumption of alcohol, obesity, and viral hepatitis. *Lancet* 2014;384(9958):1953-97. doi: 10.1016/S0140-6736(14)61838-9
2. De Angelis D, Sweeting M, Ades A, et al. An evidence synthesis approach to estimating Hepatitis C prevalence in England and Wales. *Stat Methods Med Res* 2009;18(4):361-79. doi: 0962280208094691 [pii];10.1177/0962280208094691 [doi]
3. Harris RJ, Ramsay M, Hope VD, et al. Hepatitis C prevalence in England remains low and varies by ethnicity: an updated evidence synthesis. *Eur J Public Health* 2012;22(2):187-92. doi: ckr083 [pii];10.1093/eurpub/ckr083 [doi]
4. Hutchinson SJ, Roy KM, Wadd S, et al. Hepatitis C virus infection in Scotland: epidemiological review and public health challenges. *Scott Med J* 2006;51(2):8-15.
5. Prevost TC, Presanis AM, Taylor A, et al. Estimating the number of people with hepatitis C virus who have ever injected drugs and have yet to be diagnosed: an evidence synthesis approach for Scotland. *Addiction* 2015;110(8):1287-300. doi: 10.1111/add.12948
6. Hutchinson SJ, McIntyre PG, Molyneaux P, et al. Prevalence of hepatitis C among injectors in Scotland 1989-2000: declining trends among young injectors halt in the late 1990s. *Epidemiol Infect* 2002;128(3):473-77.
7. Martin NK, Foster GR, Vilar J, et al. HCV treatment rates and sustained viral response among people who inject drugs in seven UK sites: real world results and modelling of treatment impact. *J Viral Hepat* 2015;22(4):399. doi: 10.1111/jvh.12338
8. McLeod A, Weir A, Aitken C, et al. Rise in testing and diagnosis associated with Scotland's Action Plan on Hepatitis C and introduction of dried blood spot testing. *J Epidemiol Community Health* 2014;68(12):1182-8. doi: 10.1136/jech-2014-204451
9. Palmateer NE, Hutchinson SJ, Innes H, et al. Review and meta-analysis of the association between self-reported sharing of needles/syringes and hepatitis C virus prevalence and incidence among people who inject drugs in Europe. *IntJDrug Policy* 2013;24(2):85-100. doi: S0955-3959(12)00123-5 [pii];10.1016/j.drugpo.2012.08.006 [doi]
10. Palmateer NE, Taylor A, Goldberg DJ, et al. Rapid decline in HCV incidence among people who inject drugs associated with national scale-up in coverage of a combination of harm reduction interventions. *PLoS One* 2014;9(8):e104515. doi: 10.1371/journal.pone.0104515
11. Roy KM, Hutchinson SJ, Wadd S, et al. Hepatitis C virus infection among injecting drug users in Scotland: a review of prevalence and incidence data and the methods used to generate them. *Epidemiol Infect* 2007;135(3):433-42.
12. Turner KM, Hutchinson S, Vickerman P, et al. The impact of needle and syringe provision and opiate substitution therapy on the incidence of hepatitis C virus in injecting drug users: pooling of UK evidence. *Addiction* 2011;106(11):1978-88. doi: 10.1111/j.1360-0443.2011.03515.x [doi]
13. Harris RJ, Hope VD, Morongiu A, et al. Spatial mapping of hepatitis C prevalence in recent injecting drug users in contact with services. *Epidemiol Infect* 2011:1-10. doi: S0950268811001634 [pii];10.1017/S0950268811001634 [doi]
14. Hickman M, Hope V, Brady T, et al. Hepatitis C virus (HCV) prevalence, and injecting risk behaviour in multiple sites in England in 2004. *J Viral Hepat* 2007;14(9):645-52.
15. Hope VD, Hickman M, Ngui SL, et al. Measuring the incidence, prevalence and genetic relatedness of hepatitis C infections among a community recruited sample of injecting drug users, using dried blood spots. *J Viral Hepat* 2011;18:262-70. doi: JVH1297 [pii];10.1111/j.1365-2893.2010.01297.x [doi]

For peer review only - http://bmjopen.bmj.com/site/about/guidelines.xhtml

16. Sutton AJ, Gay NJ, Edmunds WJ, et al. Modelling the force of infection for hepatitis B and hepatitis C in injecting drug users in England and Wales. *BMC InfectDis* 2006;6:93. doi: 1471-2334-6-93 [pii];10.1186/1471-2334-6-93 [doi]
17. Sweeting MJ, Hope VD, Hickman M, et al. Hepatitis C infection among injecting drug users in England and Wales (1992-2006): there and back again? *Am J Epidemiol* 2009;170(3):352-60. doi: kwp141 [pii];10.1093/aje/kwp141 [doi]
18. Wiessing L, Ferri M, Grady B, et al. Hepatitis C virus infection epidemiology among people who inject drugs in Europe: a systematic review of data for scaling up treatment and prevention. *PLoS One* 2014;9(7):e103345. doi: 10.1371/journal.pone.0103345
19. Hickman MM, N., editor. *Hepatitis C among drug users in Europe: epidemiology, treatment and prevention*. Lisbon: EMCDDA, 2016.
20. Van Den Berg C, Smit C, Van BG, et al. Full participation in harm reduction programmes is associated with decreased risk for human immunodeficiency virus and hepatitis C virus: evidence from the Amsterdam Cohort Studies among drug users. *Addiction* 2007;102(9):1454-62. doi: ADD1912 [pii];10.1111/j.1360-0443.2007.01912.x [doi]
21. Hagan H, Pouget ER, Des Jarlais DC. A systematic review and meta-analysis of interventions to prevent hepatitis C virus infection in people who inject drugs. *J InfectDis* 2011;204(1):74-83. doi: jir196 [pii];10.1093/infdis/jir196 [doi]
22. Platt L, Reed J, Minozzi S, et al. Effectiveness of needle/syringe programmes and opiate substitution therapy in preventing HCV transmission among people who inject drugs. *Cochrane Database Syst Rev* 2016;2016(1) doi: 10.1002/14651858.CD012021
23. Hutchinson SJ, Dillon JF, Fox R, et al. Expansion of HCV treatment access to people who have injected drugs through effective translation of research into public health policy: Scotland's experience. *Int J Drug Policy* 2015 doi: 10.1016/j.drugpo.2015.05.019
24. Fraser H, Mukandavire C, Martin NK, et al. Modelling the impact of a national scale-up of interventions on hepatitis C virus transmission among people who inject drugs in Scotland. *Addiction* 2018 doi: 10.1111/add.14267 [published Online First: 2018/05/22]
25. de Vos AS, van der Helm JJ, Matser A, et al. Decline in incidence of HIV and hepatitis C virus infection among injecting drug users in Amsterdam; evidence for harm reduction? *Addiction* 2013;108(6):1070-81. doi: 10.1111/add.12125
26. Vickerman P, Martin N, Turner K, et al. Can needle and syringe programmes and opiate substitution therapy achieve substantial reductions in hepatitis C virus prevalence? Model projections for different epidemic settings. *Addiction* 2012;107(11):1984-95. doi: 10.1111/j.1360-0443.2012.03932.x [doi]
27. Dore GJ, Feld JJ. Hepatitis C virus therapeutic development: in pursuit of "perfectovir". *Clin Infect Dis* 2015;60(12):1829-36. doi: 10.1093/cid/civ197
28. Gogela NA, Lin MV, Wisocky JL, et al. Enhancing our understanding of current therapies for hepatitis C virus (HCV). *Curr HIV/AIDS Rep* 2015;12(1):68-78. doi: 10.1007/s11904-014-0243-7
29. Walker DR, Pedrosa MC, Manthena SR, et al. Early View of the Effectiveness of New Direct-Acting Antiviral (DAA) Regimens in Patients with Hepatitis C Virus (HCV). *Adv Ther* 2015;32(11):1117-27. doi: 10.1007/s12325-015-0258-5
30. Hellard M, Doyle JS, Sacks-Davis R, et al. Eradication of hepatitis C infection: The importance of targeting people who inject drugs. *Hepatology* 2014;59(2):366-69. doi: 10.1002/hep.26623
31. Hagan LM, Wolpe PR, Schinazi RF. Treatment as prevention and cure towards global eradication of hepatitis C virus. *Trends in Microbiology* 2013;21(12):625-33. doi: http://dx.doi.org/10.1016/j.tim.2013.09.008
32. Grebely J, Matthews GV, Lloyd AR, et al. Elimination of hepatitis C virus infection among people who inject drugs through treatment as prevention: feasibility and future requirements. *Clin Infect Dis* 2013;57(7):1014-20. doi: 10.1093/cid/cit377

For peer review only - http://bmjopen.bmj.com/site/about/guidelines.xhtml

ISRCTN72038467

33. Bruggmann P. Treatment as prevention: The breaking of taboos is required in the fight against hepatitis C among people who inject drugs. *Hepatology* 2013;58(5):1523-25. doi: 10.1002/hep.26539
34. Martin NK, Vickerman P, Grebely J, et al. HCV treatment for prevention among people who inject drugs: Modeling treatment scale-up in the age of direct-acting antivirals. *Hepatology* 2013 doi: 10.1002/hep.26431 [doi]
35. Wedemeyer H, Duberg AS, Buti M, et al. Strategies to manage hepatitis C virus (HCV) disease burden. *J Viral Hepat* 2014;21 Suppl 1:60-89. doi: 10.1111/jvh.12249
36. Fraser H, Zibbell J, Hoerger T, et al. Scaling-up HCV prevention and treatment interventions in rural United States-model projections for tackling an increasing epidemic. *Addiction* 2018;113(1):173-82. doi: 10.1111/add.13948 [published Online First: 2017/07/25]
37. Organization WH. Global Health Sector Strategy on Viral Hepatitis 2016-2021: Towards ending viral hepatitis. In: Organization WH, ed. Geneva: WHO, 2016.
38. Innes HA, McDonald SA, Dillon JF, et al. Toward a more complete understanding of the association between a hepatitis C sustained viral response and cause-specific outcomes. *Hepatology* 2015;62(2):355-64. doi: 10.1002/hep.27766
39. Innes HA, Hutchinson SJ, Allen S, et al. Excess liver-related morbidity of chronic hepatitis C patients, who achieve a sustained viral response, and are discharged from care. *Hepatology* 2011;54(5):1547-58. doi: 10.1002/hep.24561
40. Innes H, Goldberg D, Dillon J, et al. Strategies for the treatment of Hepatitis C in an era of interferon-free therapies: what public health outcomes do we value most? *Gut* 2014 doi: 10.1136/gutjnl-2014-308166
41. Harris RJ, Martin NK, Rand E, et al. New treatments for hepatitis C virus (HCV): scope for preventing liver disease and HCV transmission in England. *J Viral Hepat* 2016 doi: 10.1111/jvh.12529
42. European Association for the Study of the L. EASL recommendations on treatment of hepatitis C 2014. *J Hepatol* 2014;61(2):373-95. doi: 10.1016/j.jhep.2014.05.001
43. European Association for the Study of the Liver. Electronic address eee. EASL Recommendations on Treatment of Hepatitis C 2015. *J Hepatol* 2015;63(1):199-236. doi: 10.1016/j.jhep.2015.03.025
44. Panel AIHG. Hepatitis C guidance: AASLD-IDSA recommendations for testing, managing, and treating adults infected with hepatitis C virus. *Hepatology* 2015 doi: 10.1002/hep.27950
45. Grebely J, Robaeys G, Bruggmann P, et al. Recommendations for the management of hepatitis C virus infection among people who inject drugs. *Int J Drug Policy* 2015;26(10):1028-38. doi: 10.1016/j.drugpo.2015.07.005
46. European Association for the Study of the Liver. Electronic address eee. EASL Recommendations on Treatment of Hepatitis C 2016. *J Hepatol* 2016 doi: 10.1016/j.jhep.2016.09.001
47. Martin NK, Vickerman P, Dore GJ, et al. Prioritization of HCV treatment in the direct-acting antiviral era: An economic evaluation. *J Hepatol* 2016;65(1):17-25. doi: 10.1016/j.jhep.2016.02.007
48. Aleman S, Rahbin N, Weiland O, et al. A risk for hepatocellular carcinoma persists long-term after sustained virologic response in patients with hepatitis C-associated liver cirrhosis. *Clin Infect Dis* 2013;57(2):230-6. doi: 10.1093/cid/cit234
49. van der Meer AJ, Veldt BJ, Feld JJ, et al. Association between sustained virological response and all-cause mortality among patients with chronic hepatitis C and advanced hepatic fibrosis. *JAMA* 2012;308(24):2584-93. doi: 10.1001/jama.2012.144878
50. Innes H, Hutchinson SJ, Obel N, et al. Liver mortality attributable to chronic hepatitis C virus infection in Denmark and Scotland--using spontaneous resolvers as the benchmark comparator. *Hepatology* 2016;63(5):1506-16. doi: 10.1002/hep.28458
51. Hickman M, De Angelis D, Vickerman P, et al. HCV Treatment as Prevention in People Who Inject Drugs–testing the evidence. *Current opinion in infectious diseases* 2015;28(6):576.

For peer review only - http://bmjopen.bmj.com/site/about/guidelines.xhtml

ISRCTN72038467

52. Martin NK, Vickerman P, Dore GJ, et al. The hepatitis C virus epidemics in key populations (including people who inject drugs, prisoners and MSM): the use of direct-acting antivirals as treatment for prevention. *Current opinion in HIV and AIDS* 2015;10(5):374-80. doi: 10.1097/COH.0000000000000179
53. Fraser H, Martin NK, Brummer-Korvenkontio H, et al. Model projections on the impact of HCV treatment in the prevention of HCV transmission among people who inject drugs in Europe. *J Hepatol* 2018;68(3):402-11. doi: 10.1016/j.jhep.2017.10.010 [published Online First: 2017/10/31]
54. Judd A, Parry J, Hickman M, et al. Evaluation of a modified commercial assay in detecting antibody to hepatitis C virus in oral fluids and dried blood spots. *J Med Virol* 2003;71(1):49-55.
55. Bennett S, Gunson RN, McAllister GE, et al. Detection of hepatitis C virus RNA in dried blood spots. *J Clin Virol* 2012;54(2):106-09. doi: S1386-6532(12)00053-4 [pii];10.1016/j.jcv.2012.02.004 [doi]
56. Martin NK, Vickerman P, Brew IF, et al. Is increased hepatitis C virus case-finding combined with current or 8-week to 12-week direct-acting antiviral therapy cost-effective in UK prisons? A prevention benefit analysis. *Hepatology* 2016;63(6):1796-808. doi: 10.1002/hep.28497
57. MacArthur GJ, Minozzi S, Martin N, et al. Opiate substitution treatment and HIV transmission in people who inject drugs: systematic review and meta-analysis. *BMJ* 2012;345:e5945.
58. MacArthur GJ, van VE, Palmateer N, et al. Interventions to prevent HIV and Hepatitis C in people who inject drugs: A review of reviews to assess evidence of effectiveness. *IntJ Drug Policy* 2013 doi: S0955-3959(13)00116-3 [pii];10.1016/j.drugpo.2013.07.001 [doi]
59. Palmateer N, Hutchinson S, McAllister G, et al. Risk of transmission associated with sharing drug injecting paraphernalia: analysis of recent hepatitis C virus (HCV) infection using cross-sectional survey data. *J Viral Hepat* 2014;21(1):25-32. doi: 10.1111/jvh.12117
60. Palmateer N, Kimber J, Hickman M, et al. Evidence for the effectiveness of sterile injecting equipment provision in preventing hepatitis C and human immunodeficiency virus transmission among injecting drug users: a review of reviews. *Addiction* 2010;105(5):844-59. doi: ADD2888 [pii];10.1111/j.1360-0443.2009.02888.x [doi]
61. Presanis AM, Gill ON, Chadborn TR, et al. Insights into the rise in HIV infections, 2001 to 2008: a Bayesian synthesis of prevalence evidence. *AIDS* 2010;24(18):2849-58. doi: 10.1097/QAD.0b013e32834021ed [doi]
62. England PH. Hepatitis C in the UK 2016 report: Working towards its elimination as a major public health threat. In: Harris HE, ed. London: Public Health England, 2016.
63. Health Protection Scotland UotWoS, Glasgow Caledonian University and the West of Scotland Specialist Virology Centre. The Needle Exchange Surveillance Initiative: Prevalence of blood-borne viruses and injecting risk behaviours among people who inject drugs attending injecting equipment provision services in Scotland, 2008-09 to 2015-16. In: Scotland HP, ed. Glasgow, 2017.
64. Brant LJ, Hurrelle M, Balogun MA, et al. Sentinel laboratory surveillance of hepatitis C antibody testing in England: understanding the epidemiology of HCV infection. *Epidemiol Infect* 2007;135(3):417-26. doi: 10.1017/S0950268806006832
65. Brant LJ, Hurrelle M, Balogun MA, et al. Where are people being tested for anti-HCV in England? Results from sentinel laboratory surveillance. *J Viral Hepat* 2008;15(10):729-39. doi: 10.1111/j.1365-2893.2008.01000.x
66. Lattimore S, Irving W, Collins S, et al. Using surveillance data to determine treatment rates and outcomes for patients with chronic hepatitis C virus infection. *Hepatology* 2014;59(4):1343-50. doi: 10.1002/hep.26926
67. Ward Z, Platt L, Sweeney S, et al. Impact of current and scaled-up levels of hepatitis C prevention and treatment interventions for people who inject drugs in three UK settings-what is

For peer review only - http://bmjopen.bmj.com/site/about/guidelines.xhtml

ISRCTN72038467

required to achieve the WHO's HCV elimination targets? *Addiction* 2018 doi: 10.1111/add.14217 [published Online First: 2018/05/19]

68. Abadie A DA, Hainmuelle, J. Comparative Politics and the Synthetic Control Method. *American Journal of Political Science* 2015;59:15.
69. Abadie A DA, Hainmueller J. Synthetic Control Methods for Comparative Case Studies: Estimating the Effect of California's Tobacco Control

Program. *Journal of the American Statistical Association* 2010;105:12.

70. Bouttell J, Craig P, Lewsey J, et al. Synthetic control methodology as a tool for evaluating population-level health interventions. *J Epidemiol Community Health* 2018 doi: 10.1136/jech-2017-210106 [published Online First: 2018/04/15]
71. Brodersen KH, Gallusser F, Koehler J, et al. Inferring causal impact using Bayesian structural time-series models. 2015:247-74. doi: 10.1214/14-AOAS788
72. Scott S VH. Predicting the present with bayesian structural time series. *International Journal of Mathematical Modelling and Numerical Optimisation* 2014;2014(5):23.
73. Treloar C, Newland J, Rance J, et al. Uptake and delivery of hepatitis C treatment in opiate substitution treatment: perceptions of clients and health professionals. *J Viral Hepat* 2010;17(12):839-44. doi: JVH1250 [pii];10.1111/j.1365-2893.2009.01250.x [doi]
74. Treloar C, Rance J, Dore GJ, et al. Barriers and facilitators for assessment and treatment of hepatitis C virus infection in the opioid substitution treatment setting: insights from the ETHOS study. *J Viral Hepat* 2014;21(8):560-7. doi: 10.1111/jvh.12183
75. Harris M, Rhodes T. Hepatitis C treatment access and uptake for people who inject drugs: a review mapping the role of social factors. *Harm Reduct J* 2013;10:7. doi: 10.1186/1477-7517-10-7
76. Swan D, Long J, Carr O, et al. Barriers to and facilitators of hepatitis C testing, management, and treatment among current and former injecting drug users: a qualitative exploration. *AIDS Patient Care STDS* 2010;24(12):753-62. doi: 10.1089/apc.2010.0142
77. Rhodes T, Harris M, Martin A. Negotiating access to medical treatment and the making of patient citizenship: the case of hepatitis C treatment. *Sociol Health Illn* 2013;35(7):1023-44. doi: 10.1111/1467-9566.12018
78. Harris M, Albers E, Swan T. The promise of treatment as prevention for hepatitis C: Meeting the needs of people who inject drugs? *Int J Drug Policy* 2015;26(10):963-9. doi: 10.1016/j.drugpo.2015.05.005
79. Rance J, Treloar C, Group ES. 'Not just methadone Tracy': transformations in service-user identity following the introduction of hepatitis C treatment into Australian opiate substitution settings. *Addiction* 2014;109(3):452-9.
80. Treloar C, Rance J, Backmund M. Understanding barriers to hepatitis C virus care and stigmatization from a social perspective. *Clin Infect Dis* 2013;57 Suppl 2:S51-5. doi: 10.1093/cid/cit263
81. Treloar C, Rance J, Bryant J, et al. Harm reduction workers and the challenge of engaging couples who inject drugs in hepatitis C prevention. *Drug Alcohol Depend* 2016;168:170-75. doi: 10.1016/j.drugalcdep.2016.09.010
82. Treloar C, Rance J, Bryant J, et al. Understanding decisions made about hepatitis C treatment by couples who inject drugs. *J Viral Hepat* 2016;23(2):89-95. doi: 10.1111/jvh.12451
83. Jack K, Islip N, Linsley P, et al. Prison officers' views about hepatitis C testing and treatment: A qualitative enquiry. *J Clin Nurs* 2016 doi: 10.1111/jocn.13489
84. Rich ZC, Chu C, Mao J, et al. Facilitators of HCV treatment adherence among people who inject drugs: a systematic qualitative review and implications for scale up of direct acting antivirals. *BMC Public Health* 2016;16:994. doi: 10.1186/s12889-016-3671-z
85. Harris M. Managing expense and expectation in a treatment revolution: Problematizing prioritisation through an exploration of hepatitis C treatment 'benefit'. *Int J Drug Policy* 2017;47:161-68. doi: 10.1016/j.drugpo.2017.03.015 [published Online First: 2017/04/30]

For peer review only - http://bmjopen.bmj.com/site/about/guidelines.xhtml

ISRCTN72038467

86. Rance J, Newland J, Hopwood M, et al. The politics of place(ment): problematising the provision of hepatitis C treatment within opiate substitution clinics. *Soc Sci Med* 2012;74(2):245-53. doi: S0277-9536(11)00644-7 [pii];10.1016/j.socscimed.2011.10.003 [doi]
87. Lankenau SE, Sanders B, Hathazi D, et al. Recruiting and retaining mobile young injection drug users in a longitudinal study. *Subst Use Misuse* 2010;45(5):684-99. doi: 10.3109/10826081003594914
88. Stewart KE, Wright PB, Sims D, et al. The "translators": engaging former drug users as key research staff to design and implement a risk reduction program for rural cocaine users. *Subst Use Misuse* 2012;47(5):547-54. doi: 10.3109/10826084.2011.644379
89. Harris M. "Three in the Room": Embodiment, Disclosure, and Vulnerability in Qualitative Research. *Qual Health Res* 2015;25(12):1689-99. doi: 10.1177/1049732314566324
90. Braun V, Clarke V. Using thematic analysis in psychology. *Qualitative Research in Psychology* 2006;3(2):77-101. doi: 10.1191/1478088706qp063oa
91. Rogers EM. Diffusion of preventive innovations. *Addict Behav* 2002;27(6):989-93.
92. Cane J, O'Connor D, Michie S. Validation of the theoretical domains framework for use in behaviour change and implementation research. *Implement Sci* 2012;7:37. doi: 10.1186/1748-5908-7-37
93. Clark JA, Gifford AL. Resolute efforts to cure hepatitis C: Understanding patients' reasons for completing antiviral treatment. *Health (London)* 2015;19(5):473-89. doi: 10.1177/1363459314555237
94. Batchelder AW, Peyser D, Nahvi S, et al. "Hepatitis C treatment turned me around:" Psychological and behavioral transformation related to hepatitis C treatment. *Drug Alcohol Depend* 2015;153:66-71. doi: 10.1016/j.drugalcdep.2015.06.007
95. Aspinall EJ, Hutchinson SJ, Janjua NZ, et al. Trends in mortality after diagnosis of hepatitis C virus infection: an international comparison and implications for monitoring the population impact of treatment. *J Hepatol* 2015;62(2):269-77. doi: 10.1016/j.jhep.2014.09.001
96. Aspinall EJ, Corson S, Doyle JS, et al. Treatment of hepatitis C virus infection among people who are actively injecting drugs: a systematic review and meta-analysis. *Clin Infect Dis* 2013;57 Suppl 2:S80-9. doi: 10.1093/cid/cit306
97. Innes HA, Hutchinson SJ, Allen S, et al. Ranking predictors of a sustained viral response for patients with chronic hepatitis C treated with pegylated interferon and ribavirin in Scotland. *Eur J Gastroenterol Hepatol* 2012;24(6):646-55. doi: 10.1097/MEG.0b013e32835201a4
98. McDonald SA, Hutchinson SJ, Bird SM, et al. Excess morbidity in the hepatitis C-diagnosed population in Scotland, 1991-2006. *Epidemiol Infect* 2011;139(3):344-53. doi: 10.1017/S0950268810001421
99. McDonald SA, Hutchinson SJ, Bird SM, et al. A record-linkage study of the development of hepatocellular carcinoma in persons with hepatitis C infection in Scotland. *Br J Cancer* 2008;99(5):805-10.
100. McDonald SA, Hutchinson SJ, Bird SM, et al. A population-based record linkage study of mortality in hepatitis C-diagnosed persons with or without HIV coinfection in Scotland. *Stat Methods Med Res* 2009;18(3):271-83. doi: 10.1177/0962280208094690
101. Gao L, Dimitropoulou P, Robertson JR, et al. Risk-factors for methadone-specific deaths in Scotland's methadone-prescription clients between 2009 and 2013. *Drug Alcohol Depend* 2016;167:214-23. doi: 10.1016/j.drugalcdep.2016.08.627
102. Bird SM, Fischbacher CM, Graham L, et al. Impact of opioid substitution therapy for Scotland's prisoners on drug-related deaths soon after prisoner release. *Addiction* 2015;110(10):1617-24. doi: 10.1111/add.12969
103. Bird SM, Hutchinson SJ. Male drugs-related deaths in the fortnight after release from prison: Scotland, 1996-99. *Addiction* 2003;98(2):185-90.

For peer review only

For peer review only - http://bmjopen.bmj.com/site/about/guidelines.xhtml

ISRCTN72038467

104. Alvarez-Madrazo S, McTaggart S, Nangle C, et al. Data Resource Profile: The Scottish National Prescribing Information System (PIS). *Int J Epidemiol* 2016;45(3):714-15f. doi: 10.1093/ije/dyw060
105. Graham L, Fischbacher CM, Stockton D, et al. Understanding extreme mortality among prisoners: a national cohort study in Scotland using data linkage. *Eur J Public Health* 2015;25(5):879-85. doi: 10.1093/eurpub/cku252
106. Merrall EL, Bird SM, Hutchinson SJ. Mortality of those who attended drug services in Scotland 1996-2006: record-linkage study. *Int J Drug Policy* 2012;23(1):24-32. doi: 10.1016/j.drugpo.2011.05.010
107. McDonald SA, Hutchinson SJ, Bird SM, et al. Hospitalisation for an alcohol-related cause among injecting drug users in Scotland: increased risk following diagnosis with hepatitis C infection. *Int J Drug Policy* 2011;22(1):63-9. doi: 10.1016/j.drugpo.2010.04.003
108. de Vos AS, Prins M, Kretzschmar ME. Hepatitis C virus treatment as prevention among injecting drug users: who should we cure first? *Addiction* 2015;110(6):975-83. doi: 10.1111/add.12842
109. Boily MC, Lowndes CM, Vickerman P, et al. Evaluating large-scale HIV prevention interventions: study design for an integrated mathematical modelling approach. *Sex TransmInfect* 2007;83(7):582-89. doi: sti.2007.027516 [pii];10.1136/sti.2007.027516 [doi]
110. Boily MC, Pickles M, Lowndes CM, et al. Positive impact of a large-scale HIV prevention program among female sex workers and clients in Karnataka state, India. *AIDS* 2013 doi: 10.1097/QAD.0b013e32835fba81 [doi]
111. Hallett TB, Gregson S, Mugurungi O, et al. Assessing evidence for behaviour change affecting the course of HIV epidemics: a new mathematical modelling approach and application to data from Zimbabwe. *Epidemics* 2009;1(2):108-17. doi: 10.1016/j.epidem.2009.03.001
112. Pickles M, Boily MC, Vickerman P, et al. Assessment of the population-level effectiveness of the Avahan HIV-prevention programme in South India: a preplanned, causal-pathway-based modelling analysis. *Lancet Glob Health* 2013;1(5):e289-99. doi: 10.1016/S2214-109X(13)70083-4
113. Martin NK, Hickman M, Miners A, et al. Cost-effectiveness of HCV case-finding for people who inject drugs via dried blood spot testing in specialist addiction services and prisons. *BMJ open* 2013;3(8) doi: 10.1136/bmjopen-2013-003153
114. Martin NK, Miners A, Vickerman P. Assessing the cost-effectiveness of interventions aimed at promoting and offering hepatitis C testing to injecting drug users: An economic modelling report. London, 2012.
115. Martin NK, Pitcher AB, Vickerman P, et al. Optimal control of hepatitis C antiviral treatment programme delivery for prevention amongst a population of injecting drug users. *PLoS One* 2011;6(8):e22309. doi: 10.1371/journal.pone.0022309 [doi];PONE-D-10-01568 [pii]
116. Martin NK, Vickerman P, Miners A, et al. The cost-effectiveness of HCV antiviral treatment for injecting drug user populations. *Hepatology* 2012;55(1):49-57. doi: 10.1002/hep.24656 [doi]
117. Martin NK, Vickerman P, Miners A, et al. How cost-effective is hepatitis C virus treatment for people who inject drugs? *Journal of gastroenterology and hepatology* 2013;28(4):590-2. doi: 10.1111/jgh.12113
118. Kimber J, Copeland L, Hickman M, et al. Survival and cessation in injecting drug users: prospective observational study of outcomes and effect of opiate substitution treatment. *BMJ* 2010;341:c3172.
119. McDonald SA, Hutchinson SJ, Palmateer NE, et al. Decrease in health-related quality of life associated with awareness of hepatitis C virus infection among people who inject drugs in Scotland. *JHepatol* 2013;58(3):460-66. doi: S0168-8278(12)00836-7 [pii];10.1016/j.jhep.2012.11.004 [doi]
120. Wright M, Grieve R, Roberts J, et al. Health benefits of antiviral therapy for mild chronic hepatitis C: randomised controlled trial and economic evaluation. *Health Technol Assess* 2006;10(21):1-113, iii. doi: 95-24-03 [pii]

For peer review only - http://bmjopen.bmj.com/site/about/guidelines.xhtml

ISRCTN72038467

For peer review only
For peer review only - http://bmjopen.bmj.com/site/about/guidelines.xhtml

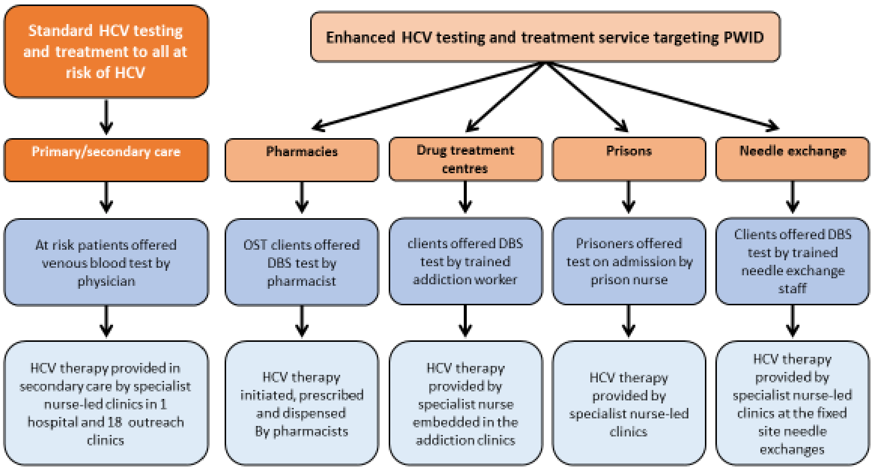

PWID defined as those who either (a) are currently injecting drugs, (b) have ever injected drugs and are currently on opioid substitute therapy, or (c) have ever injected drugs and are currently in prison

DBS: dried blood spot; OST: opioid substitution therapies; PWID: people who inject drugs

For peer review only

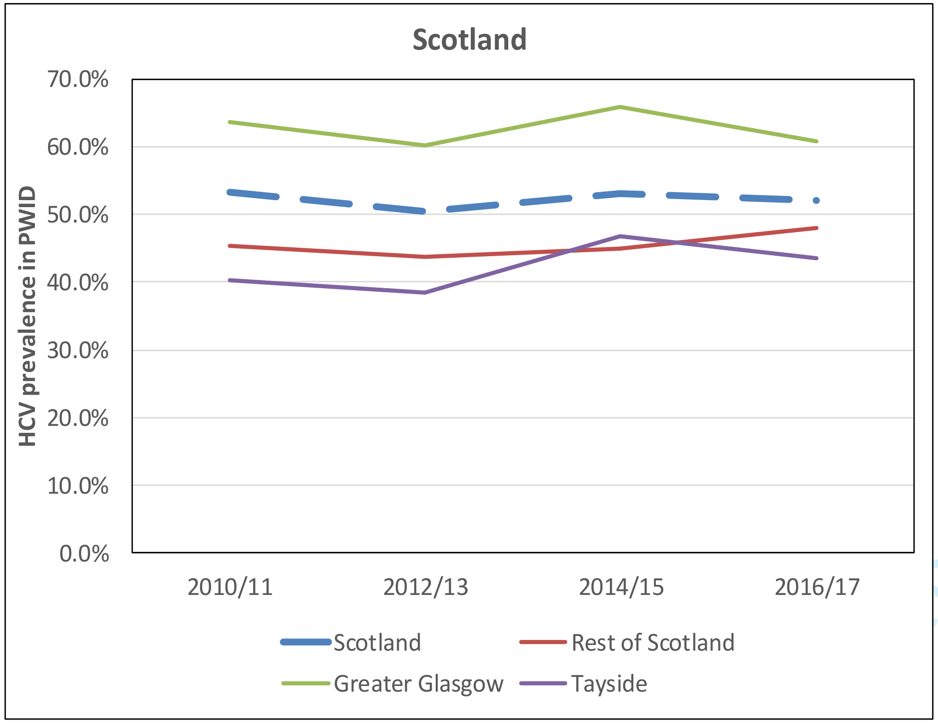
Scotland
70.0%
60.0%
50.0%
40.0%
30.0%
20.0%
10.0%
0.0%
HCV prevalence in PWID
2010/11
2012/13
2014/15
2016/17
Scotland
Rest of Scotland
Greater Glasgow
Tayside

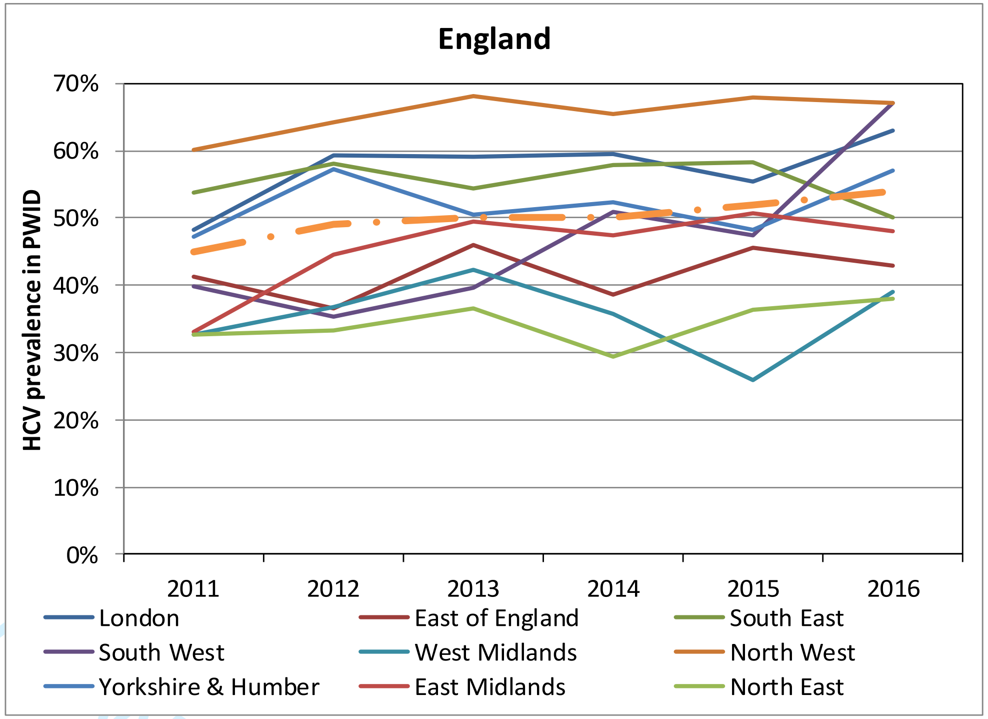
England
70%
60%
50%
40%
30%
20%
10%
0%
HCV prevalence in PWID
2011
2012
2013
2014
2015
2016
London
East of England
South East
South West
West Midlands
North West
Yorkshire & Humber
East Midlands
North East

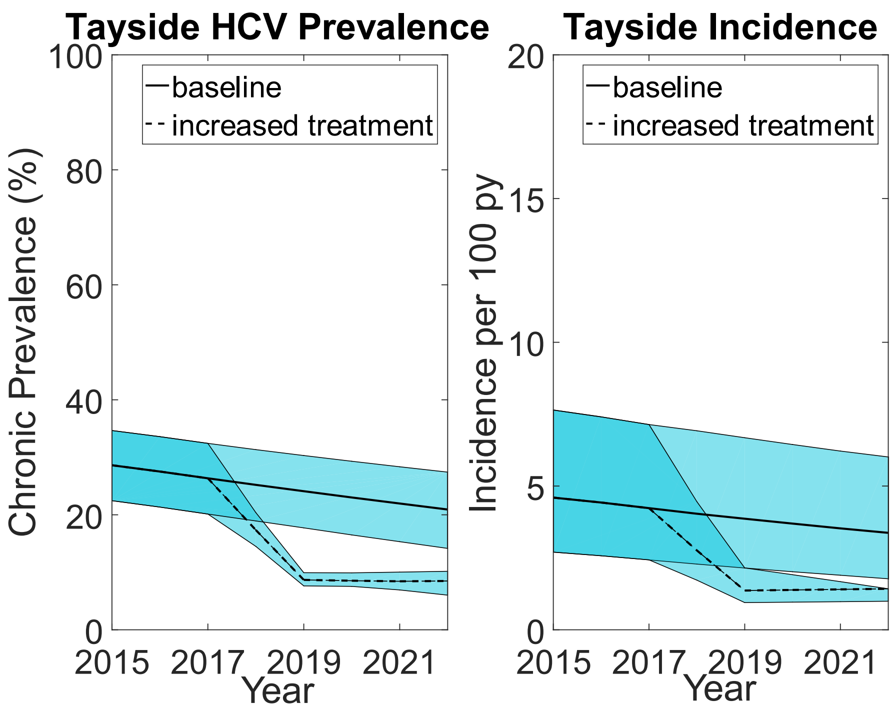
Tayside HCV Prevalence
Chronic Prevalence (%)
100
80
60
40
20
0
2015 2017 2019 2021
Year
baseline
increased treatment
Tayside Incidence
Incidence per 100 py
20
15
10
5
0
2015 2017 2019 2021
Year
baseline
increased treatment

iew only

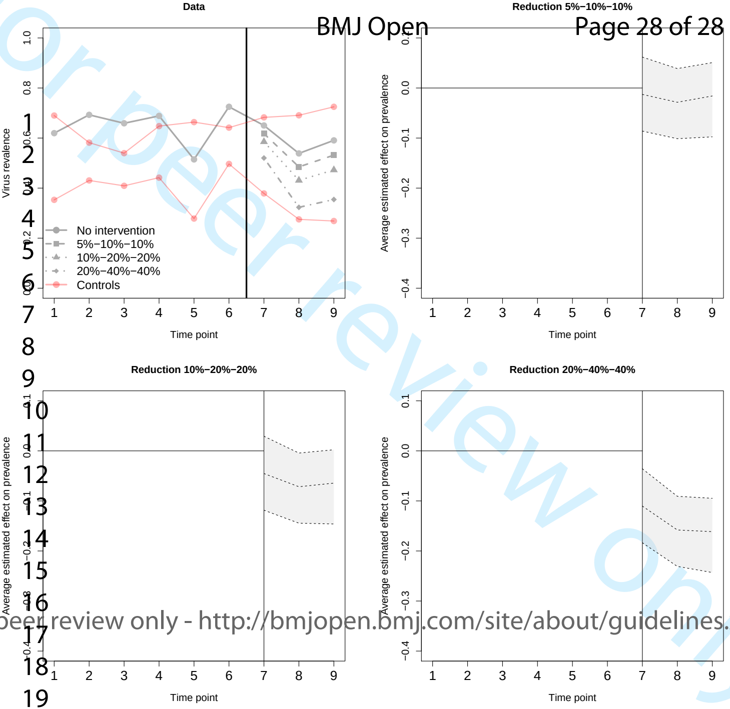

peer review only - http://bmjopen.bmj.com/site/about/guidelines.x

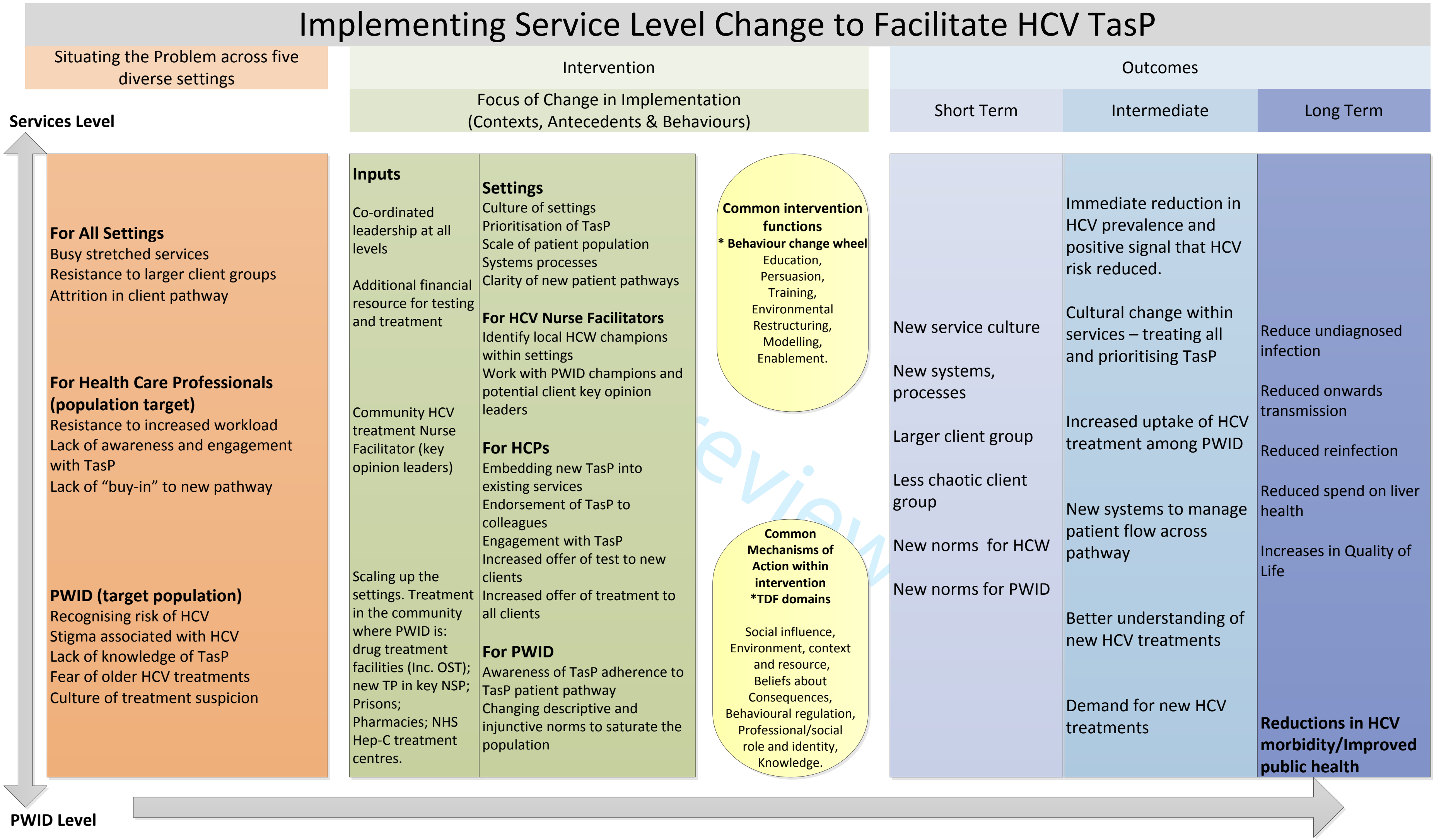
Implementing Service Level Change to Facilitate HCV TasP
Situating the Problem across five diverse settings
Intervention
Focus of Change in Implementation (Contexts, Antecedents & Behaviours)
Outcomes
Short Term
Intermediate
Long Term
Services Level
PWID Level
For All Settings
Busy stretched services
Resistance to larger client groups
Attrition in client pathway
For Health Care Professionals (population target)
Resistance to increased workload
Lack of awareness and engagement with TasP
Lack of "buy-in" to new pathway
PWID (target population)
Recognising risk of HCV
Stigma associated with HCV
Lack of knowledge of TasP
Fear of older HCV treatments
Culture of treatment suspicion
Inputs
Co-ordinated leadership at all levels
Additional financial resource for testing and treatment
Community HCV treatment Nurse Facilitator (key opinion leaders)
Scaling up the settings. Treatment in the community where PWID is: drug treatment facilities (Inc. OST); new TP in key NSP; Prisons; Pharmacies; NHS Hep-C treatment centres.
Settings
Culture of settings
Prioritisation of TasP
Scale of patient population
Systems processes
Clarity of new patient pathways
For HCV Nurse Facilitators
Identify local HCW champions within settings
Work with PWID champions and potential client key opinion leaders
For HCPs
Embedding new TasP into existing services
Endorsement of TasP to colleagues
Engagement with TasP
Increased offer of test to new clients
Increased offer of treatment to all clients
For PWID
Awareness of TasP adherence to TasP patient pathway
Changing descriptive and injunctive norms to saturate the population
Common intervention functions
* Behaviour change wheel
Education, Persuasion, Training, Environmental Restructuring, Modelling, Enablement.
Common Mechanisms of Action within intervention
*TDF domains
Social influence, Environment, context and resource, Beliefs about Consequences, Behavioural regulation, Professional/social role and identity, Knowledge.
New service culture
New systems, processes
Larger client group
Less chaotic client group
New norms for HCW
New norms for PWID
Immediate reduction in HCV prevalence and positive signal that HCV risk reduced.
Cultural change within services – treating all and prioritising TasP
Increased uptake of HCV treatment among PWID
New systems to manage patient flow across pathway
Better understanding of new HCV treatments
Demand for new HCV treatments
Reduce undiagnosed infection
Reduced onwards transmission
Reduced reinfection
Reduced spend on liver health
Increases in Quality of Life
Reductions in HCV morbidity/Improved public health

BMJ Open

Evaluating the Population Impact of Hepatitis C Direct Acting Antiviral Treatment as Prevention for People Who Inject Drugs (EPIToPe) – a natural experiment

Journal:	*BMJ Open*
Manuscript ID	bmjopen-2019-029538.R1
Article Type:	Protocol
Date Submitted by the Author:	26-Apr-2019

Complete List of Authors:	Hickman, M; Bristol<br>Dillon, John; University of Dundee Division of Cardiovascular and Diabetes Medicine, Liver Group<br>Elliott, Lawrie; Glasgow Caledonian University, Department of Nursing and Community Health<br>De Angelis, Daniela; MRC Biostatistics Unit<br>Vickerman, Peter; London School of Hygiene and Tropical Medicine; University of Bristol, School of Social and Community Medicine<br>Foster, Graham; Barts and The London School of Medicine<br>Donnan, Peter; University of Dundee, Dundee Epidemiology and Biostatistics Unit<br>Eriksen, Ann; Tayside Health Board<br>Flowers, Paul; Glasgow Caledonian University,<br>Goldberg, David; Health Protection Scotland<br>Hollingworth, William; University of Bristol, School of Social and Community Medicine<br>Ijaz, Samreen; Public Health England Centre for Infection<br>Liddell, David; Scottish Drugs Forum<br>Mandal, Sema; Public Health England, Immunisation, Hepatitis, Blood Safety and Countermeasures Response; National Institute for Health Research Health Protection Unit<br>Martin, Natasha; University of California, San Diego<br>Beer, Lewis; University of Dundee, Tayside Clinical Trials Unit<br>Drysdale, Kate; Barts Health NHS Trust<br>Fraser, Hannah; University of Bristol, School of Social and Community Medicine<br>Glass, Rachel; Public Health England, Immunisation, Hepatitis, Blood Safety and Countermeasures Response<br>Graham, Lesley; ISD Scotland<br>Gunson, Rory; Gartnavel General Hospital, West Of Scotland Specialist Virology Centre<br>Hamilton, Emma; Scottish Drug Forum<br>Harris, Helen; Public Health England, Immunisation, Hepatitis, Blood Safety and Countermeasures Response<br>Harris, Magdalena; London School of Hygiene and Tropical Medicine, Centre for Research on Drugs and Health Behaviours<br>Harris, Ross; Public Health England, Immunisation, Hepatitis, Blood Safety and Countermeasures Response<br>Heinsbroek, Ellen; Public Health England Colindale

	Hope, Vivian; Liverpool John Moores University<br>Horwood, Jeremy; University of Bristol, UK, School of Social and Community Medicine<br>Inglis, Sarah; University of Dundee, Division of Neuoscience; University of Dundee, Tayside Clinical Trials Unit<br>Innes, Hamish; Glasgow Caledonian University, School of health and life sciences<br>Lane, Athene; University of Bristol, School of Social and Community Medicine<br>Meadows, Jade; University of Bristol School of Social and Community Medicine<br>McAuley, Andrew; NHS Health Scotland<br>Metcalfe, Chris; University of Bristol, Social Medicine<br>Migchelsen, Stephanie; Public Health England, Immunisation, Hepatitis, Blood Safety and Countermeasures Response<br>Murray, Alex; Scottish Drug Forum<br>Myring, Gareth; University of Bristol School of Social and Community Medicine<br>Palmateer, Norah E.; Hlth Protect Scotland, Blood-borne viruses and sexually transmitted infections; Glasgow Caledonian University,<br>Presanis, Anne; Institute of Public Health, MRC Biostatistics Unit<br>Ramsay, Mary; Public Health England, immunisation; London School of Hygiene and Tropical Medicine, Epidemiology<br>Samartsidis, Pantelis; MRC Biostatistics Unit<br>Simmons, Ruth; Public Health England,<br>Sinka, Katy; Public Health England, Immunisation, Hepatitis, Blood Safety and Countermeasures Response<br>Vojt, Gabriele; Glasgow Caledonian University<br>Ward, Zoe; Bristol, Population Health Sciences, Bristol Medical School<br>Whiteley, David; Edinburgh Napier University<br>Yeung, Alan; Glasgow Caledonian University<br>Hutchinson, Sharon; Health Protection Scotland, ; Glasgow Caledonian University, School of Health and Life Sciences
**Primary Subject Heading**:	Public health
Secondary Subject Heading:	Addiction, Epidemiology, Gastroenterology and hepatology, Infectious diseases, Qualitative research
Keywords:	Hepatology < INTERNAL MEDICINE, INFECTIOUS DISEASES, Epidemiology < INFECTIOUS DISEASES, PUBLIC HEALTH, Public health < INFECTIOUS DISEASES, Infection control < INFECTIOUS DISEASES

SCHOLARONE™
Manuscripts

ISRCTN72038467

Evaluating the **P**opulation **I**mpact of Hepatitis C Direct Acting Antiviral **T**reatment as Prevention f**o**r **Pe**ople Who Inject Drugs (EPIToPe) – a natural experiment (Protocol)

Authors:

Hickman M[1], Dillon J[2], Elliott L[3], De Angelis D[4], Vickerman P[1], Foster G[5], Donnan PT[2], Eriksen A[6], Flowers P[3], Goldberg D[7], Hollingworth W[1], Ijaz S[8], Liddell D[9], Mandal S[8], Martin NK[10,1], Beer L[2] , Drysdale K[5], Fraser H[1], Glass R[8], Graham L[11], Gunson R[12], Hamilton E[9], Harris H[8], Harris M[13], Harris R[8], Heinsbroek E[8], Hope V[14], Horwood J[1], Inglis S[2], Innes H[3], Lane A[1], Meadows, J[1], McAuley A[7], Metcalfe C[1], Migchelsen S[8], Murray, A[9], Myring G [1], Palmateer N[3], Presanis A[4], Ramsay M[8], Samartsidis P[4], Simmons R[8], Sinka K[8], Vojt, G[3] , Ward Z[1], Whiteley D[15], Yeung, A[3] , Hutchinson S[3]

Correspondence to: Prof Matthew Hickman; Matthew.Hickman@bristol.ac.uk

Affiliations

1 Population Health Sciences, Bristol Medical School, University of Bristol

2 University of Dundee

3 Glasgow Caledonian University

4 MRC Biostatistics Unit, University of Cambridge

5 Barts Liver Centre, Queen Mary University of London

6 Tayside Health Board

7 Health Protection Scotland

8 Public Health England

9 Scottish Drug Forum

10 Division of Infectious Diseases and Global Public Health, University of California San Diego

11 Information Services Division, Scotland

12 West of Scotland Specialist Virology Centre, Glasgow Royal Infirmary

13 London School of Hygiene and Tropical Medicine

14 Liverpool John Moores University

15 Edinburgh Napier University

For peer review only

ISRCTN72038467

ABSTRACT

Introduction:

Hepatitis C Virus (HCV) is the second largest contributor to liver disease in the UK, with injecting drug use as the main risk factor among the estimated 200,000 people currently infected. Despite effective prevention interventions, chronic HCV prevalence remains at 40% among people who inject drugs (PWID). New Direct Acting Antiviral (DAA) HCV therapies combine high cure rates (>90%) and short treatment duration (8-12 weeks). Theoretical mathematical modelling evidence suggests HCV treatment scale-up can prevent transmission and substantially reduce HCV prevalence/incidence among PWID. Our primary aim is to generate empirical evidence on the effectiveness of HCV "Treatment as Prevention" (TasP) in PWID.

Methods and Analysis

We plan to establish a natural experiment with Tayside, Scotland, as a single intervention site where HCV care pathways are being expanded (including specialist drug treatment clinics, needle & syringe programmes (NSPs), pharmacies, and prison) and HCV treatment for PWID is being rapidly scaled-up. Other sites in Scotland and England will act as potential controls. Over two years from 2017/18, at least 500 PWID will be treated in Tayside, which simulation studies project will reduce chronic HCV prevalence among PWID by 62% (from 26% to 10%) and HCV incidence will fall by approximately 2/3 (from 4.2 per 100 person-years (p100py) to 1.4 p100py). Treatment response and re-infection rates will be monitored. We will conduct focus groups and interviews with service providers and patients that accept and decline treatment to identify barriers and facilitators in implementing TasP. We will conduct longitudinal interviews with up to 40 PWID to assess whether successful HCV treatment alters their perspectives on and engagement with drug treatment and recovery. Trained peer researchers will be involved in data collection and dissemination.

The primary outcome – chronic HCV prevalence in PWID – is measured using information from the Needle Exchange Surveillance Initiative (NESI) survey in Scotland and the Unlinked Anonymous Monitoring Programme (UAM) in England, conducted at least four times before and three times during and after the intervention. We will adapt Bayesian synthetic control methods (also called Causal Inference Models) to generate the cumulative impact of the intervention on chronic HCV prevalence and incidence. We will use a dynamic HCV transmission and economic model to evaluate the cost-effectiveness of the HCV TasP intervention, and to estimate the contribution of the scale up in HCV treatment to observed changes in HCV prevalence. Through the qualitative data we will systematically explore key mechanisms of TasP real world implementation from provider and patient perspectives to develop a manual for scaling up HCV treatment in other settings. We will compare qualitative accounts of drug treatment and recovery with a "virtual cohort" of PWID linking information on HCV treatment with Scottish Drug treatment databases to test whether DAA treatment improves drug treatment outcomes.

Ethics and Dissemination

Extending HCV community care pathways is covered by ethics (ERADICATE C, ISRCTN27564683, Super DOT C Trial clinicaltrials.gov:NCT02706223). Ethical approval for extra data collection from patients including health utilities and qualitative interviews has been granted (INSERT) and ISCRCTN registration has been completed (INSERT). Our findings will have direct NHS and patient relevance;

For peer review only - http://bmjopen.bmj.com/site/about/guidelines.xhtml

informing prioritization given to early HCV treatment for PWID. We will present findings to practitioners and policy makers, and support design of an evaluation of HCV TasP in England.

Strengths and limitations of this study

1. Our control sites in the rest of Scotland and England were not randomised so there will be confounding and uncertainty in the intervention effect estimates. We consider our natural experiment design to be more robust than simple before and after studies and our preliminary simulation work suggests that we should have sufficient power to detect the large intervention effect that is planned.

2. HCV treatment and prevention strategy in UK (and Europe) is evolving - motivated both by WHO "elimination targets" and falling drug prices – and our control sites may increase treatment rates earlier than expected which will complicate the analyses and potentially dilute the intervention effect.

3. The counterfactual of "no HCV treatment scale-up" has to be generated by our transmission model so that we can estimate cost-effectiveness of the intervention in Tayside. This is not ideal but has become standard practice in economic models of HCV treatment interventions – especially as the benefit in terms of additional Quality of Life Years accrues and occurs over a prolonged period. We are using a dynamic model which means that the prevention benefit (in terms of HCV infections averted) can be incorporated into the cost-effectiveness calculations – which is essential in evaluating HCV interventions in people who inject drugs.

4. We are using peer researchers in the qualitative arm of patients' perspectives on the intervention and on the impact of HCV treatment on addiction outcomes. This is novel but adds additional challenges. We have trained the interviewers and will be monitoring their performance of the interviewers to ensure consistent study quality – and will replace peers with our qualitative researcher if required.

For peer review only

For peer review only - http://bmjopen.bmj.com/site/about/guidelines.xhtml

ISRCTN72038467

Introduction and Background:

Infection with Hepatitis C Virus (HCV) is a progressive disease that over 20-40 years can lead to liver cancer and premature death. HCV is the second largest contributor to liver disease in the UK and one of the few causes that is curable[1]. In the UK it is estimated that approximately 200,000 people are infected with HCV, over 85% of whom are people who inject or have injected drugs (PWID)[2-5]. Chronic HCV prevalence and incidence among PWID remains high in UK at 20-50% and 5 to 15 per 100 person- years respectively[4 6-18]. Prevention of HCV transmission among PWID is critical to long-term prevention of HCV related liver disease[19].

We have reviewed the effectiveness of traditional primary prevention against HCV –opioid substitution treatment (OST) and needle and syringe programmes (NSPs)[12 20-22]. Ongoing exposure to OST and high-coverage NSPs can reduce the risk of HCV transmission by 50-80%[12 22]. In Scotland HCV incidence among PWID decreased from approximately 14 to 6 per 100 person- years from 2008/09 to 2011/12 coinciding with the launch of the Scottish HCV strategy and action plan which incorporated scale-up of harm reduction interventions and HCV treatment[10 23]. We estimated that 60% of this decline could be attributed to the scale-up of OST and NSP during the action plan and that 1,400 HCV infections were averted by 2015[24]. However, there was no appreciable reduction in overall anti-HCV prevalence over this short period, and there is some suggestion that incidence has increased recently to ~10 per 100 person years (http://www.hps.scot.nhs.uk/resourcedocument.aspx?id=5863). HCV transmission models suggest that primary prevention through NSP and OST alone is insufficient to achieve substantial reductions (of the order of 40% or more within ten years) in HCV prevalence among PWID in the UK[25 26].

Prevention of hepatitis C disease and HCV transmission is now possible because highly effective, tolerable, short-course interferon-free direct acting antiviral therapies (DAAs) are available for all HCV genotypes with cure rates – defined as sustained virological response (SVR)- exceeding 90%[27-29]. We, and others, hypothesise that HCV Treatment scale-up for PWID, and resulting HCV Treatment as Prevention (TasP) could enhance other primary interventions and reduce HCV incidence and chronic prevalence to negligible levels (i.e. towards elimination as a major public health concern)[30-35]. TasP refers to the concept whereby future transmission is reduced by treating affected individuals[36 37]: in HIV TasP Antiretroviral Treatment reduces transmission because individuals have undetectable infection[38]; in HCV TasP people are cured so reducing opportunities for future transmission. World Health Organization (WHO) targets for HCV elimination, adopted by UK and other countries, aim to reduce HCV incidence by 80% and associated mortality by 65% by 2030[39 40 41 42 43 44].

Clinical guidelines in Europe and US changed from recommending prioritising HCV treatment to people with moderate to severe liver disease towards removing any restrictions and recommending that people at risk of transmission irrespective of fibrosis stage are offered treatment[45-49]. Cost-effectiveness models that incorporate the population prevention benefit suggest early treatment should be prioritised to PWID over other patient groups (unless chronic HCV prevalence and transmission is very high)[50]. There is direct evidence that SVR following HCV treatment reduces liver disease progression and mortality risk[51-53], but in two recent reviews we found no empirical evidence that HCV treatment scale-up has reduced chronic HCV prevalence and incidence in PWID populations[36 37]. In part this is because in most settings HCV treatment rates in PWID are too low and any changes generally too small to be detected, as we show in two studies of seven sites in UK[7] and an extension to 11 sites in Europe[54]. Until very recently in the UK, the annual number of HCV

For peer review only - http://bmjopen.bmj.com/site/about/guidelines.xhtml

ISRCTN72038467

DAA treatments was restricted -as drug costs could be expensive (>£10,000 per patient). There is the opportunity now to test whether scaling up HCV treatment will reduce chronic HCV prevalence and transmission among PWID[44].

In a pilot study ("Eradicate C") in Tayside we showed that we can increase HCV case-finding and engage and successfully treat PWID in the community[55]. Combining further studies on extending community HCV treatment pathways in Tayside and additional treatments provided by NHS Tayside and Scottish Government we can establish an immediate natural experiment (with Tayside as the intervention site and other sites in Scotland and England as controls) to test and generate UK empirical evidence on the and potential impact and cost-effectiveness of HCV "Treatment as Prevention" (TasP) in people who inject drugs (PWID). The UK is one of few countries worldwide to have an established nationwide surveillance system monitoring HCV infection among PWID [9 12 17 22 56-60]. This is undertaken through a series of cross-sectional voluntary anonymous surveys of PWID recruited at harm reduction services, referred to as the Unlinked Anonymous Monitoring Programme (UAM) in England and Wales and the Needle Exchange Surveillance Initiative (NESI) in Scotland[61 62]. In addition, the UK has established sentinel laboratory surveillance of HCV testing and national monitoring of HCV treatment [8 63-65]. The data collected in both UAM and NESI will be used to assess out outcome.

Alongside a natural experiment in Tayside, we will collect information to assess the treatment facilitators and barriers. Historically it has proven very hard to engage PWID in HCV treatment[66-69]. Some barriers to engagement, such as poor efficacy or fear of interferon treatment side-effects, may be ameliorated by DAA therapy. However, other barriers such as mistrust of health services, stigma, and competing priorities faced by PWID may persist. In addition, providers may be reticent to refer or provide HCV treatment to PWID due to concerns about adherence, reinfection and perceptions of treatment 'worth'[70 71]. It is expected that co-locating HCV treatment within existing services will reduce many system and provider level barriers to PWID accessing care[66-68 72-77]. However, this has not been tested in the context of community wide scale-up of interventions across multiple potential pathways. It is critical, therefore, that we understand how HCV TasP is embedded within the existing service landscape and incorporated into providers' professional roles.

Finally it has been hypothesised that successful HCV treatment in PWID may positively impact on understandings of self and identity and improve treatment of drug use disorders[71 72 78-80]. Accounts of 'transformative' outcomes extending beyond viral clearance alone include reference to reductions in drug and alcohol use, uptake of safer injecting practices, improved social relationships, enhanced sense of responsibility and self-worth. Hints of such collateral or indirect benefits are also found in quantitative studies reporting low re-infection rates and reductions in risky injecting behaviours among treated PWID[81 82]. We aim to test this hypothesis in our qualitative follow-up study and compare the findings to quantitative data generated from a virtual cohort.

For peer review only - http://bmjopen.bmj.com/site/about/guidelines.xhtml

ISRCTN72038467

Methods and analysis:

Study design

Our intention is to create and conduct a mixed methods study, including qualitative studies and economic evaluation, of a natural experiment of HCV Treatment as Prevention (TasP) among People who Inject Drugs (PWID). We also will develop methods for evaluating HCV TasP.

Methods

Scaling-up HCV treatment

The **intervention** comprises the scale-up of HCV treatment in People Who Inject Drugs (PWID) which has started early in Tayside. . By combining support from Scottish Government, National Health Board Tayside (NHS Tayside) and industry (MSD, Gilead, BMS) we can deliver rapid intensive scale-up of HCV treatments for PWID (comprising an extra 400 HCV treatments, a 3.5-fold increase from treatments for PWID prior to April 2017, see sample size below). We have developed multiple integrated community HCV care pathways, including novel care pathways in pharmacies, a low threshold NSP, drug treatment services and prisons (see Figure 1). Our diagnostic pathways make extensive use of dried blood spot (DBS) testing for diagnosis of HCV antibody and chronic HCV with subsequent conventional laboratory testing in preparation for treatment (viral load, liver function and Fib4 fibrosis score)[83-85].

Study population

Our intervention is delivered and measured at the population level – which we have created by combining several individual studies and treatment pathways as shown in Figure 1 (see ethics section below for the individual studies). We gained ethical approval East of Scotland Research Ethics Service REC 1 (ref: 18/ES/0128) to ask patients for permission to be recruited into the qualitative study (below) and extended clinical and behavioural drug history and data on health utilities (EQ5D-5L) at onset of treatment, during treatment and after the end of treatment.

Community HCV specialist nurses (3.5 FTE) coordinate and deliver case-finding and treatment across the pathways in Tayside (Figure 1).

Figure 1: Overview of HCV testing and treatment pathways for the PWID population in NHS Tayside.

The region of Tayside co-localises to NHS Tayside which is the provider of health care to a geographical area of 2,903 sq mi (7519 km2) including the cities of Dundee and Perth and the counties of Angus and Perth & Kinross, situated in the east of Scotland with a population of 416,000. It is a mixture of urban and rural environments with some of the most affluent and most deprived areas in Scotland. It is therefore a representative microcosm of many areas in the UK.

HCV treatment

Apart from expansion of community HCV care pathways, no new clinical procedures will be investigated and all PWID with chronic HCV will be offered oral DAA HCV treatment compliant with the Scottish clinical guidelines (https://www.hps.scot.nhs.uk/resourcedocument.aspx?id=6621).

For peer review only - http://bmjopen.bmj.com/site/about/guidelines.xhtml

ISRCTN72038467

As per local standard of care, participants will be offered appropriate harm reduction advice.

Standard care for patients is to test for SVR at 12 weeks after end of treatment with patients being recommended for annual follow-up if at risk of re-infection. Specialist nurses concentrate on building a good relationship with the participant to ensure that they do return for follow-up appointments. Health Protection Scotland collates national public health surveillance data on the number, characteristics and response of patients initiated onto HCV therapy, through Clinical Databases installed in 17 specialist HCV treatment centres, across Scotland[41 86]. A similar system also is available in England.

HCV surveillance and Intervention Outcome (Chronic HCV in PWID)

The **outcome** is chronic HCV prevalence (HCV viraemia as measured by HCV PCR) among PWID in the community (not just in the patients who undergo HCV treatment). Prevalence will be monitored using the NESI and UAM surveys, as detailed below. During 2017-22, three waves of data collection for NESI (n=7,500) and five to six for UAM (n=17,000 in England) will measure this outcome.

In our pre-intervention period from 2010/11 to 2016 there have been four NESI surveys in Scotland (n=10,000 participants in total) and six UAM surveys in England (n=16,000 in total), which have involved the collection of DBS linked to questionnaire data. Participants are recruited at sentinel sites by a team of trained interviewers in Scotland (at over 100 NSP sites) and by agency staff in over 60 low-threshold drug agencies across England[58 61]. Participants complete a short questionnaire, with common questions across UAM and NESI, on demographics, injecting behaviour and service utilisation, and importantly (in relation to quantifying the intervention effect) both survey approaches have remained consistent over time.

The DBS samples collected in NESI and UAM have all been tested for HCV antibody, using the same methods (where sensitivity and specificity of the assay on DBS are close to 100%)[83 84], and illustrate that antibody prevalence (ever infection) has remained relatively stable among PWID during this time (Figure 2). PCR positivity among antibody positive samples is used to determine chronic infection.

All NESI and UAM samples will be tested for HCV antibody and RNA PCR to assess the impact of HCV therapy scale-up – which is critical as trends in chronic infection and antibody status will diverge as more people are cured. In addition, we will undertake RNA PCR testing of all historical samples that were HCV antibody positive shown in Figure 2 so that we can measure chronic HCV prevalence among PWID pre-, as well as post-, intervention for analysis (below)

Figure 2: Trends in HCV antibody prevalence among PWID in Scotland and England 2010/11-16

Data on HCV PCR positivity among antibody negative samples identify recent infections and is used to estimate HCV incidence – which has fluctuated between 5-10 infections per 100 person years across the UK during the last five years[61]. We will also estimate HCV incidence from our transmission dynamic models[24 54].

Sample size, Power, and Estimating Intervention Effect

We updated estimates of the prevalence of PWID in Tayside[5] which suggest there are 2,760 (95% Credible Interval, CrI 2,360-3,170) PWID either currently injecting and/or in OST. NESI data suggest

For peer review only - http://bmjopen.bmj.com/site/about/guidelines.xhtml

ISRCTN72038467

that approximately 30% have chronic HCV and over 75% of PWID with chronic HCV have been diagnosed. Prior to 2017 approximately 66 PWID were treated annually. From April 2017 we plan to treat at least 500 PWID in Tayside over 2 years (as a result of expanded community care pathways shown in Figure 1 and extra HCV treatments provided by NHS, Scottish Government and Industry funding). Adapting a transmission dynamic model that has been used in Tayside,[87] we hypothesize that within two years chronic HCV prevalence among PWID will reduce by approximately 62% from 26% (95% CrI 20-32) to at least 10% and chronic HCV incidence will fall by approximately 2/3s from 4.2 (95% CrI 2.4-7.1) per 100 person-years (p100py) to 1.4 (95%CrI 1.0 – 1.4) p100py (as shown in Figure 3). Modelling also suggests that maintaining these reductions after 2019 will require less than 40 treatments per year.

Figure 3: Projected chronic HCV prevalence and incidence among PWID in Tayside with and without the intervention. Blue shaded area denotes the 95% credibility intervals of the model projections with and without the intervention

We will adapt synthetic control methods or Causal Impact Model as proposed by Brodersen and colleagues[88 89].

We have performed simulation studies to test power and evaluate the utility of the CIM assuming information on chronic HCV prevalence among PWID (shown in Figure 4). Provided trends in the chronic HCV prevalence in the pre-intervention period are relatively stable (which is the case) there will be sufficient power to detect the projected reduction in chronic prevalence. For example, in Figure 4d we see that for a prevalence reduction of 40% by year 2-3 the credible intervals of the estimated cumulative effect (cumulative drop in prevalence) exclude zero, correctly identifying evidence of a successful intervention. Whereas a cumulative reduction of <20% is unlikely to be detected.

Figure 4: Causal Impact Synthetic Control Method (CIM) simulation and estimated intervention effects and 95% Credible Intervals for a range of assumed effects.

Footnote:- Illustration of CIM. First subplot shows a single dataset, where solid lines represent the simulated prevalence in the absence of the intervention, and the dashed lines represent the outcome of treated site in the post intervention period under different intervention magnitude scenarios. For each one of the three scenarios, we calculate the estimated average intervention effect along with credible intervals. These are shown in Subplots 2-4. We see that as the effect increases, the intervals tend to move away for zero. However, the intervention effect only becomes significant in scenario 3, where zero is not included in any of the post-intervention time points.

Qualitative Studies

Understanding the barriers and facilitators to scaling-up community-based HCV treatment

The qualitative study design has two distinct arms focusing on the intervention providers, and the intervention recipients.

For peer review only - http://bmjopen.bmj.com/site/about/guidelines.xhtml

ISRCTN72038467

Intervention providers

A purposive sample of 30 intervention providers, comprising nursing leads and key individuals from collaborating organisations will be approached directly by the lead hepatitis nurse. Seven focus groups will be convened according to professional role and locality:

- HCV healthcare specialists (nurses and physicians)
- Community pharmacists
- Prison staff (both healthcare and security)
- 'Drug workers' (from OST and NSP services)

Each focus group will consist of a maximum of six individuals and ideally comprise multi-agency mixed groups. Individual interviews by telephone will be offered for those hesitant to join a group (estimate 10 interviews). Topic guides informed by previous work in this area[66 68 76 90] will facilitate group discussion.

Intervention recipients – cross-sectional and longitudinal

The intervention recipient arm of the study will comprise both cross-sectional and longitudinal elements. A cross-sectional approach will be employed to recruit 6-10 participants who do not take up the offer of treatment. These individuals will be recruited through the treatment pathways or through our peer researcher networks. The longitudinal element will follow a cohort of up to 40 individuals recruited following their course of HCV treatment. These individuals will be purposively sampled from the existing services in which HCV TasP has been embedded (i.e. pharmacy, prison and drug service), and then followed-up at one year post-treatment (with 70% expected to be followed-up)[91]. We aim to recruit women as well as men, younger and older people; those treated previously and first time; those injecting and not injecting at treatment onset. Follow-up interviews will explore collateral effects of HCV TasP including outcomes pertaining to drug use and injecting practices (secondary outcome below).

Participants will be recruited by hepatitis nurses or other clinical staff in Tayside and the face-to-face semi-structured interview will be conducted by peer-researchers, trained and guided by experienced qualitative researchers. Dr Magdalena Harris explains the importance of the use of peer researchers within the context of EPIToPe: https://www.youtube.com/watch?v=9ZZo3fKOXlg

[92 93]. The Scottish Drugs Forum (SDF) works with a group of Tayside peer-researchers with lived experience of injecting. Peer-researchers will receive study-orientated training and be provided with ongoing support to co-produce data and contribute to study outputs. A £20 shopping voucher will be offered to all interviewees except those in prison (Scottish prison service ethics did not permit thank you vouchers to prison participants).

Qualitative Data Analysis

Interviews and focus-groups will be audio-recorded using encrypted digital voice recorders, transcribed verbatim and anonymised. *Nvivo* v.10 software will be used to code and manage qualitative data. First level analysis will be deductive, guided by the research questions, and peer researchers will be consulted for input and feedback during the analytical process[94]. A constant comparison method will be used to develop the thematic analysis and will reflect diverging and converging narratives, for example, across groups of intervention recipients at different time points in the treatment pathway, or between groups of intervention providers[94]. The findings will be

For peer review only - http://bmjopen.bmj.com/site/about/guidelines.xhtml

ISRCTN72038467

contextualised in the relevant theoretical perspectives which may include the diffusion of preventive innovations (staff) or social norms and values that might underpin health behaviour (recipients)[95 96]. We will assess TasP both from the providers' perspective and from patients' perspective including those who refuse treatment.

We will use the findings iteratively to update the HCV TasP logic model shown in Figure 5. Our qualitative data will be used to generate a manual of an optimal intervention for other sites in UK**.** In previous examples, such as [https://www.youtube.com/channel/UCBV8smLmkOQVT9D0OR-md1g/videos] we have used the Behaviour Change Wheel [96] as the framework to retrospectively analyse the success and failure of implementation within Tayside and then prospectively to formulate the optimal implementation intervention.

Figure 5: Preliminary Logic Model HCV Treatment as Prevention (EPIToPe)

Mixed Method Study on drug use outcomes: OST retention, drug overdose, recovery, and social transformation

Health Protection Scotland (HPS) link data on diagnostic HCV tests in the four largest Scottish NHS boards (including Tayside)[8] and all persons undergoing HCV treatment in the Scottish HCV Clinical database[97] which are also linked with other databases (including deaths, hospitalisations and drug treatment)[8 42 98-100] and from 2018 Scotland's Prescribing Information System (PIS) which holds data on OST and NHS prison health database (Prison Vision)[101-105]. PWID attending drug services who were HCV diagnosed, compared to those who were not, are at increased risk of drug-related and other cause-specific morbidity/mortality[106 107]. Thus, we will create a virtual cohort of chronic HCV infected PWID (estimated to involve at least 600 individuals from Tayside and 3,000 from elsewhere) and through linkage identify those who have been treated and attained SVR with those who have not. We will assess and compare the following outcomes:- retention in drug treatment (determined through linkage to drug treatment and prescribing databases), drug- and alcohol- related morbidity/mortality (through linkage to all hospital admission and mortality databases), and other markers of relapse (through linkage to prisons database).

Economic and impact evaluation

Infectious disease models can test the extent to which observed changes in disease transmission can be attributed to specific interventions,[108-112] and assess cost-effectiveness of interventions that avert secondary infections, i.e. have a population prevention benefit [50 85 113-117]. We will update and adapt a transmission model of HCV among PWID in Scotland and Tayside to model the impact of the HCV treatment intervention based on historical trends and new observations collected as part of this programme[39 87]**.** We will stratify the PWID population into current (injected in the previous year) and temporarily ceased (in OST and not injected in the previous year); as well as by duration of injecting (< 3 years, 3 to 9 years, 10+ years since onset), prevention intervention exposure (OST and/or high coverage NSP), and intervention settings for testing and treatment. We will use

For peer review only - http://bmjopen.bmj.com/site/about/guidelines.xhtml

ISRCTN72038467

Approximate Bayesian Computation to calibrate the model to pre-intervention trends in chronic HCV prevalence and incidence among PWID in Tayside. The model will simulate the impact of observed rates of HCV treatment and cure rates for the intervention period, also incorporating any changes in the coverage of OST and NSP and injecting risk behaviours.

We will test consistency between the model impact projections and observed changes in HCV chronic prevalence and incidence from Tayside to disentangle the impact of HCV TasP from other interventions (OST/NSP) or epidemiological changes, and predict the impact of the TasP on number of HCV infections averted. If they are not consistent then alternative evidence-based hypotheses will be tested for why the model projects a different impact and the best fitting models will then be used to project the impact of the intervention. This will be assessed compared to two alternative counterfactuals where treatment rates are either at pre-scale-up levels in Tayside or at the average level achieved in other UK sites over the scale-up period. The impact of any changes in OST and NSP coverage will also be assessed to determine the contribution of those changes on observed effects. Impact will be assessed in terms of the relative decrease in prevalence and incidence, as well as the number and percent of infections averted in the intervention model projections compared to each counterfactual over different time frames. These model projections can also be taken forward to evaluate the possible impact of the intervention over next 5 or 10 years.

We will evaluate the cost-effectiveness of the intervention (HCV treatment scale-up) compared to status quo (expected rate of HCV case-finding and treatment among PWID in the rest of the UK) from a health care provider (NHS) perspective, with the cost-effectiveness of the different settings where case-finding occurs also being assessed. The cost-effectiveness (CE) model will be based on the same dynamic impact model, adapted to include HCV disease progression stages and tracking of health outcomes among PWID after cessation of injecting[50]. The economic evaluation will incorporate both individual benefits of HCV treatment (on disease progression) as well as population benefits (on HCV transmission). We will calculate the total number of infections and deaths over a 50-year time horizon for the intervention and counterfactual scenario and estimate the costs and quality-adjusted life years (QALYs) based on the number of individuals in each disease stage per year in the model. We will discount all future costs and QALYs at 3.5% (NICE guidelines https://www.nice.org.uk/process/pmg9/resources/guide-to-the-methods-of-technology-appraisal-2013-pdf-2007975843781). Probabilistic sensitivity analyses will be used to estimate the parametric uncertainty in the impact and cost projections. Cost-effectiveness results will be expressed in terms of incremental cost-effectiveness ratios (ICERs) and net monetary benefits (NMB) estimated using NICE thresholds (£20,000 and £30,000 per QALY). We will plot cost-effectiveness acceptability curves to determine the probability of the intervention being cost-effective compared to different willingness-to-pay thresholds. Analyses of covariance (ANCOVA) methods will be used to summarize the proportion of the variability in the incremental costs and QALYs explained by uncertainty in different input parameters. Univariate sensitivity analyses will consider the effect of changes in important parameters such as time horizon, treatment cost and discount rate.

We focus on the incremental or additional resource costs associated with the intervention in Tayside. These costs, in part based on our earlier work for other studies, will include such things as the nurse time spent on intervention related activities (training other staff to offer HCV testing and

For peer review only - http://bmjopen.bmj.com/site/about/guidelines.xhtml

ISRCTN72038467

treatment referral) as well as additional HCV testing and treatment costs, any additional OST costs due to HCV testing or treatment, and other staff time at the NSP, drug treatment centres and prisons involved with the intervention. Most of the incremental costs can be defined as variable (driven by extra nurse time and HCV testing/treatment costs). NHS HCV care costs and health utilities will be attached to each disease stage, based primarily on previous syntheses and models, which assume that PWID have a lower QoL than non-PWID of a similar age, gender and liver disease stage[118-120]. Additional data using the EQ-5D-5L tool during this study will generate new health utility data on the QoL amongst PWID before and after DAA treatment.

Patient and Public Involvement

Patient and Public Involvement (PPI) was led by the Hepatitis C Trust and supported by qualitative research assessing barriers and facilitators to HCV treatment access (led by Magdalena Harris). The Scottish Drug Forum (SDF) were also actively involved in the development of EPIToPe. The input from PPI groups has influenced the design of care pathways and has ensured that peer research is an essential element of the qualitative strand of EPIToPe.

A pilot NIHR funded study in England (HEPCAT) responding to NICE Guidance on Hepatitis Case Finding was co-designed with Hepatitis C trust. It showed that Hepatitis C Facilitators and peer support networks can increase the uptake of HCV case-finding and HCV treatment readiness in addiction services. This pilot study and our studies in Dundee/Tayside will influence how HCV treatment can be scaled up in England and our proposed evaluation HCV treatment as prevention.

Peer researchers will be trained to conduct the longitudinal study with PWID treated for HCV and will be involved and contribute to the analysis of the findings. Peer researchers and SDF will be members of the project management group and steering committee.

Dissemination events will be held in Dundee to discuss and present the findings from the qualitative studies with patient groups and services. These will be facilitated by SDF to support active contribution from our peer researchers. The study findings will be summarised and promoted through SDF website, social media platforms and through their sector-wide conferences in Scotland. Hepatitis Scotland, who are hosted within SDF, together with patient and public groups in England will take an active role in the wider national and international dissemination of the research, it's translation into patient meaningful materials and its integration into a national policy context. The research will also be promoted via Hepatitis C Trust and Public Health England.

Discussion

Strengths and limitations of this study

Several limitations arise from the "natural experiment" design as our intervention and controls were not randomised. In the UK and many other countries there is no longer sufficient equipoise in

For peer review only - http://bmjopen.bmj.com/site/about/guidelines.xhtml

clinicians and policymakers – given WHO and national strategies on HCV "elimination" - to mount an RCT of HCV Treatment as Prevention. As a result, there will be confounding and additional uncertainty in the measurement of the intervention effect. However, we consider that a natural experiment and use of synthetic control methods to be a more robust design than simple before and after studies. Our preliminary simulation work also suggests that we should have sufficient power to detect the large intervention effect that is planned.

We know also that HCV treatment and prevention strategy in UK (and Europe) is evolving - motivated both by WHO "elimination targets" and falling drug prices – and our control sites in Scotland and England may increase HCV treatment rates earlier than expected. This will complicate the analyses a little and potentially dilute the intervention effect. We are confident that we can adapt the synthetic control methods to take account of changes over time – and that because Tayside has started so early in scaling up HCV treatment that we will have time to detect a difference in the outcome.

The lack of randomised controls means that we have to generate the counterfactual of "no HCV treatment scale-up" through our HCV transmission model so that we can subsequently estimate cost-effectiveness of the intervention in Tayside. This is not ideal but has become standard practice in economic models of novel HCV treatment interventions – and we are involved with the modelling of HCV treatment pathways through homeless centres, prison, A&E, pharmacies, specialist drug clinics, and NSPs (P Vickerman personal communication and e.g. [55 85 121]. We know also, however, that the benefit in terms of additional Quality of Life Years and averted HCV infections accrues and occurs over a prolonged period [50]. It is more critical for any economic evaluation of HCV interventions in PWID that a dynamic model is used so that the prevention benefit (in terms of HCV infections averted) is correctly accounted for.

We are using peer researchers in the qualitative arm of patients' perspectives on the intervention and on the impact of HCV treatment on addiction outcomes. This is novel but adds additional challenges to obtaining NHS passports and ensuring data quality across the interviews and interviewees. We are also intending to support peers in analysis and interpretation of the findings which we believe has not been done before. We have trained the interviewers and will be monitoring their performance of the interviewers to ensure consistent study quality – and will replace peers with our qualitative researcher if required.

Future Study: Natural experiment of TasP in England

In England HCV treatment is delivered through 22 operational delivery networks (ODNs). NHS England's HCV strategy (2016-2019) prioritised 10,000 patients per year in line with the declared priorities of the network which could (and in many cases did) include people who use drugs at risk of transmission [44]. In October 2018 it is anticipated that a new procurement deal will substantially increase the number of patients who can access DAAs and this will enable 'trace and treat' options to be introduced. We will use the first part of EPIToPe including the manual generated by the qualitative study, enhancements to historical and ongoing surveillance of chronic HCV in PWID, infectious disease models, and methodological developments of causal impact model, to co-design with ODN leads a natural experiment of HCV TasP in England.

Authors' contributions:

For peer review only - http://bmjopen.bmj.com/site/about/guidelines.xhtml

ISRCTN72038467

All authors contributed to editing of the manuscript.

M Hickman (MH) and S Hutchinson (SH) are co-PIs of EPIToPe and prepared first draft of the manuscript.

J Dillon (JD) leads intervention scale-up in Tayside in collaboration with Tayside CTU (L Beer (LB), PT Donnan (PTD), S Inglis (SI) and A Eriksen (AE)).

L Elliot (LE) leads qualitative component of EPIToPe in collaboration with Scottish Drug Forum (D Liddell (DL), E Hamilton (EH) and A Murray (AM)) and support from M Harris (MHa), G Vojt (GV) and D Whitely (DW) on qualitative research and training of peer support workers, and P Flowers (PF) on behavioural science.

D De Angelis (DA) leads synthetic control estimation and multiple parameter evidence synthesis in collaboration with P Samartsidis (PS), R Harris (RA), A Presanis (AP), and N Martin (NM).

P Vickerman (PV) leads dynamic impact and economic modelling in collaboration with NM, Z Ward (ZW), H Fraser (HF) with health economics led by W Hollingworth (WH), with G Myring (GM), as part of Bristol Randomised Trial Collaboration (BRTC) with advice on trial design from J Horwood (JH), C Metcalfe (CM) and A Lane (AL).

G Foster (GF) is leading design of evaluation in England based on EPIToPe with support from BRTC and K Drysdale

SH is leading on outcome measurement in Scotland with Health Protection Scotland (D Goldberg (DG), A McAuley (AMc)), and in collaboration with L Graham (LG) from ISD, R Gunson (RG), H Innes (HI), N Palmateer (NP) and A Yeung (AY).

S Mandal (SM) and S Ijaz (SI) are leading on outcome measurement in England with R Glass (RG), H Harris (HH), E Heinsbroek (EH), V Hope (VH), S Migchelsen (SM), M Ramsay (MR), R Simmons (RS), K Sinka (SK).

J Meadows (JM) is the Programme Manager.

Funding statement: 'This paper presents independent research funded by the National Institute for Health Research (NIHR) under its Programme Grants for Applied Research Programme (Reference Number RP-PG-0616-20008). The views expressed are those of the author(s) and not necessarily those of the NHS, the NIHR or the Department of Health.'

In addition, we acknowledge support from NIHR Health Protection Research Unit in Evaluation, and the Bristol Randomised Trials Collaboration (BRTC), a UKCRC Registered Clinical Trials Unit in receipt of NIHR Clinical Trials Units (CTU) support funding. The views expressed are those of the author and not necessarily those of the NHS, the NIHR or the Department of Health and Social Care. NM is supported by the National Institute for Drug Abuse [grant number R01 DA037773] and the University of California San Diego Center for AIDS Research (CFAR), a National Institute of Health (NIH) funded program [grant number P30 AI036214].

We would like to thank all those involved in PPI from both SDF and the Hepatitis C Trust.

Competing interests statement.
MH has received unrestricted honoraria for presenting at meetings from Abbvie, Gilead, MSD. NM has received unrestricted research grants and honoraria from Gilead and Merck. PV has received

For peer review only - http://bmjopen.bmj.com/site/about/guidelines.xhtml

ISRCTN72038467

unrestricted honoraria for presenting at meetings from Abbvie and Gilead. PV has received unrestricted research grants from Gilead.

PTD has received unrestricted grants from Shire pharmaceuticals, Novo Nordisk and Gilead and is a member of the Scottish Medicines Consortium. HF has received an honorarium from MSD.

For peer review only

For peer review only - http://bmjopen.bmj.com/site/about/guidelines.xhtml

ISRCTN72038467

References

1. Williams R, Aspinall R, Bellis M, et al. Addressing liver disease in the UK: a blueprint for attaining excellence in health care and reducing premature mortality from lifestyle issues of excess consumption of alcohol, obesity, and viral hepatitis. *Lancet* 2014;384(9958):1953-97. doi: 10.1016/S0140-6736(14)61838-9
2. De Angelis D, Sweeting M, Ades A, et al. An evidence synthesis approach to estimating Hepatitis C prevalence in England and Wales. *Stat Methods Med Res* 2009;18(4):361-79. doi: 0962280208094691 [pii];10.1177/0962280208094691 [doi]
3. Harris RJ, Ramsay M, Hope VD, et al. Hepatitis C prevalence in England remains low and varies by ethnicity: an updated evidence synthesis. *Eur J Public Health* 2012;22(2):187-92. doi: ckr083 [pii];10.1093/eurpub/ckr083 [doi]
4. Hutchinson SJ, Roy KM, Wadd S, et al. Hepatitis C virus infection in Scotland: epidemiological review and public health challenges. *Scott Med J* 2006;51(2):8-15.
5. Prevost TC, Presanis AM, Taylor A, et al. Estimating the number of people with hepatitis C virus who have ever injected drugs and have yet to be diagnosed: an evidence synthesis approach for Scotland. *Addiction* 2015;110(8):1287-300. doi: 10.1111/add.12948
6. Hutchinson SJ, McIntyre PG, Molyneaux P, et al. Prevalence of hepatitis C among injectors in Scotland 1989-2000: declining trends among young injectors halt in the late 1990s. *Epidemiol Infect* 2002;128(3):473-77.
7. Martin NK, Foster GR, Vilar J, et al. HCV treatment rates and sustained viral response among people who inject drugs in seven UK sites: real world results and modelling of treatment impact. *J Viral Hepat* 2015;22(4):399. doi: 10.1111/jvh.12338
8. McLeod A, Weir A, Aitken C, et al. Rise in testing and diagnosis associated with Scotland's Action Plan on Hepatitis C and introduction of dried blood spot testing. *J Epidemiol Community Health* 2014;68(12):1182-8. doi: 10.1136/jech-2014-204451
9. Palmateer NE, Hutchinson SJ, Innes H, et al. Review and meta-analysis of the association between self-reported sharing of needles/syringes and hepatitis C virus prevalence and incidence among people who inject drugs in Europe. *IntJDrug Policy* 2013;24(2):85-100. doi: S0955-3959(12)00123-5 [pii];10.1016/j.drugpo.2012.08.006 [doi]
10. Palmateer NE, Taylor A, Goldberg DJ, et al. Rapid decline in HCV incidence among people who inject drugs associated with national scale-up in coverage of a combination of harm reduction interventions. *PLoS One* 2014;9(8):e104515. doi: 10.1371/journal.pone.0104515
11. Roy KM, Hutchinson SJ, Wadd S, et al. Hepatitis C virus infection among injecting drug users in Scotland: a review of prevalence and incidence data and the methods used to generate them. *Epidemiol Infect* 2007;135(3):433-42.
12. Turner KM, Hutchinson S, Vickerman P, et al. The impact of needle and syringe provision and opiate substitution therapy on the incidence of hepatitis C virus in injecting drug users: pooling of UK evidence. *Addiction* 2011;106(11):1978-88. doi: 10.1111/j.1360-0443.2011.03515.x [doi]
13. Harris RJ, Hope VD, Morongiu A, et al. Spatial mapping of hepatitis C prevalence in recent injecting drug users in contact with services. *Epidemiol Infect* 2011:1-10. doi: S0950268811001634 [pii];10.1017/S0950268811001634 [doi]
14. Hickman M, Hope V, Brady T, et al. Hepatitis C virus (HCV) prevalence, and injecting risk behaviour in multiple sites in England in 2004. *J Viral Hepat* 2007;14(9):645-52.
15. Hope VD, Hickman M, Ngui SL, et al. Measuring the incidence, prevalence and genetic relatedness of hepatitis C infections among a community recruited sample of injecting drug users, using dried blood spots. *J Viral Hepat* 2011;18:262-70. doi: JVH1297 [pii];10.1111/j.1365-2893.2010.01297.x [doi]

For peer review only - http://bmjopen.bmj.com/site/about/guidelines.xhtml

ISRCTN72038467

16. Sutton AJ, Gay NJ, Edmunds WJ, et al. Modelling the force of infection for hepatitis B and hepatitis C in injecting drug users in England and Wales. *BMC InfectDis* 2006;6:93. doi: 1471-2334-6-93 [pii];10.1186/1471-2334-6-93 [doi]
17. Sweeting MJ, Hope VD, Hickman M, et al. Hepatitis C infection among injecting drug users in England and Wales (1992-2006): there and back again? *Am J Epidemiol* 2009;170(3):352-60. doi: kwp141 [pii];10.1093/aje/kwp141 [doi]
18. Wiessing L, Ferri M, Grady B, et al. Hepatitis C virus infection epidemiology among people who inject drugs in Europe: a systematic review of data for scaling up treatment and prevention. *PLoS One* 2014;9(7):e103345. doi: 10.1371/journal.pone.0103345
19. Hickman MM, N., editor. *Hepatitis C among drug users in Europe: epidemiology, treatment and prevention*. Lisbon: EMCDDA, 2016.
20. Van Den Berg C, Smit C, Van BG, et al. Full participation in harm reduction programmes is associated with decreased risk for human immunodeficiency virus and hepatitis C virus: evidence from the Amsterdam Cohort Studies among drug users. *Addiction* 2007;102(9):1454-62. doi: ADD1912 [pii];10.1111/j.1360-0443.2007.01912.x [doi]
21. Hagan H, Pouget ER, Des Jarlais DC. A systematic review and meta-analysis of interventions to prevent hepatitis C virus infection in people who inject drugs. *J InfectDis* 2011;204(1):74-83. doi: jir196 [pii];10.1093/infdis/jir196 [doi]
22. Platt L, Reed J, Minozzi S, et al. Effectiveness of needle/syringe programmes and opiate substitution therapy in preventing HCV transmission among people who inject drugs. *Cochrane Database Syst Rev* 2016;2016(1) doi: 10.1002/14651858.CD012021
23. Hutchinson SJ, Dillon JF, Fox R, et al. Expansion of HCV treatment access to people who have injected drugs through effective translation of research into public health policy: Scotland's experience. *Int J Drug Policy* 2015 doi: 10.1016/j.drugpo.2015.05.019
24. Fraser H, Mukandavire C, Martin NK, et al. Modelling the impact of a national scale-up of interventions on hepatitis C virus transmission among people who inject drugs in Scotland. *Addiction* 2018 doi: 10.1111/add.14267 [published Online First: 2018/05/22]
25. de Vos AS, van der Helm JJ, Matser A, et al. Decline in incidence of HIV and hepatitis C virus infection among injecting drug users in Amsterdam; evidence for harm reduction? *Addiction* 2013;108(6):1070-81. doi: 10.1111/add.12125
26. Vickerman P, Martin N, Turner K, et al. Can needle and syringe programmes and opiate substitution therapy achieve substantial reductions in hepatitis C virus prevalence? Model projections for different epidemic settings. *Addiction* 2012;107(11):1984-95. doi: 10.1111/j.1360-0443.2012.03932.x [doi]
27. Dore GJ, Feld JJ. Hepatitis C virus therapeutic development: in pursuit of "perfectovir". *Clin Infect Dis* 2015;60(12):1829-36. doi: 10.1093/cid/civ197
28. Gogela NA, Lin MV, Wisocky JL, et al. Enhancing our understanding of current therapies for hepatitis C virus (HCV). *Curr HIV/AIDS Rep* 2015;12(1):68-78. doi: 10.1007/s11904-014-0243-7
29. Walker DR, Pedrosa MC, Manthena SR, et al. Early View of the Effectiveness of New Direct-Acting Antiviral (DAA) Regimens in Patients with Hepatitis C Virus (HCV). *Adv Ther* 2015;32(11):1117-27. doi: 10.1007/s12325-015-0258-5
30. Hellard M, Doyle JS, Sacks-Davis R, et al. Eradication of hepatitis C infection: The importance of targeting people who inject drugs. *Hepatology* 2014;59(2):366-69. doi: 10.1002/hep.26623
31. Hagan LM, Wolpe PR, Schinazi RF. Treatment as prevention and cure towards global eradication of hepatitis C virus. *Trends in Microbiology* 2013;21(12):625-33. doi: http://dx.doi.org/10.1016/j.tim.2013.09.008
32. Grebely J, Matthews GV, Lloyd AR, et al. Elimination of hepatitis C virus infection among people who inject drugs through treatment as prevention: feasibility and future requirements. *Clin Infect Dis* 2013;57(7):1014-20. doi: 10.1093/cid/cit377

For peer review only - http://bmjopen.bmj.com/site/about/guidelines.xhtml

ISRCTN72038467

33. Bruggmann P. Treatment as prevention: The breaking of taboos is required in the fight against hepatitis C among people who inject drugs. *Hepatology* 2013;58(5):1523-25. doi: 10.1002/hep.26539
34. Martin NK, Vickerman P, Grebely J, et al. HCV treatment for prevention among people who inject drugs: Modeling treatment scale-up in the age of direct-acting antivirals. *Hepatology* 2013 doi: 10.1002/hep.26431 [doi]
35. Wedemeyer H, Duberg AS, Buti M, et al. Strategies to manage hepatitis C virus (HCV) disease burden. *J Viral Hepat* 2014;21 Suppl 1:60-89. doi: 10.1111/jvh.12249
36. Martin NK, Vickerman P, Dore GJ, et al. The hepatitis C virus epidemics in key populations (including people who inject drugs, prisoners and MSM): the use of direct-acting antivirals as treatment for prevention. *Current opinion in HIV and AIDS* 2015;10(5):374-80. doi: 10.1097/COH.0000000000000179
37. Hickman M, De Angelis D, Vickerman P, et al. HCV Treatment as Prevention in People Who Inject Drugs–testing the evidence. *Current opinion in infectious diseases* 2015;28(6):576.
38. Hayes R, Ayles H, Beyers N, et al. HPTN 071 (PopART): rationale and design of a cluster-randomised trial of the population impact of an HIV combination prevention intervention including universal testing and treatment - a study protocol for a cluster randomised trial. *Trials* 2014;15:57. doi: 10.1186/1745-6215-15-57
39. Fraser H, Zibbell J, Hoerger T, et al. Scaling-up HCV prevention and treatment interventions in rural United States-model projections for tackling an increasing epidemic. *Addiction* 2018;113(1):173-82. doi: 10.1111/add.13948 [published Online First: 2017/07/25]
40. Organization WH. Global Health Sector Strategy on Viral Hepatitis 2016-2021: Towards ending viral hepatitis. In: Organization WH, ed. Geneva: WHO, 2016.
41. Innes HA, McDonald SA, Dillon JF, et al. Toward a more complete understanding of the association between a hepatitis C sustained viral response and cause-specific outcomes. *Hepatology* 2015;62(2):355-64. doi: 10.1002/hep.27766
42. Innes HA, Hutchinson SJ, Allen S, et al. Excess liver-related morbidity of chronic hepatitis C patients, who achieve a sustained viral response, and are discharged from care. *Hepatology* 2011;54(5):1547-58. doi: 10.1002/hep.24561
43. Innes H, Goldberg D, Dillon J, et al. Strategies for the treatment of Hepatitis C in an era of interferon-free therapies: what public health outcomes do we value most? *Gut* 2014 doi: 10.1136/gutjnl-2014-308166
44. Harris RJ, Martin NK, Rand E, et al. New treatments for hepatitis C virus (HCV): scope for preventing liver disease and HCV transmission in England. *J Viral Hepat* 2016 doi: 10.1111/jvh.12529
45. European Association for the Study of the L. EASL recommendations on treatment of hepatitis C 2014. *J Hepatol* 2014;61(2):373-95. doi: 10.1016/j.jhep.2014.05.001
46. European Association for the Study of the Liver. Electronic address eee. EASL Recommendations on Treatment of Hepatitis C 2015. *J Hepatol* 2015;63(1):199-236. doi: 10.1016/j.jhep.2015.03.025
47. Panel AIHG. Hepatitis C guidance: AASLD-IDSA recommendations for testing, managing, and treating adults infected with hepatitis C virus. *Hepatology* 2015 doi: 10.1002/hep.27950
48. Grebely J, Robaeys G, Bruggmann P, et al. Recommendations for the management of hepatitis C virus infection among people who inject drugs. *Int J Drug Policy* 2015;26(10):1028-38. doi: 10.1016/j.drugpo.2015.07.005
49. European Association for the Study of the Liver. Electronic address eee. EASL Recommendations on Treatment of Hepatitis C 2016. *J Hepatol* 2016 doi: 10.1016/j.jhep.2016.09.001
50. Martin NK, Vickerman P, Dore GJ, et al. Prioritization of HCV treatment in the direct-acting antiviral era: An economic evaluation. *J Hepatol* 2016;65(1):17-25. doi: 10.1016/j.jhep.2016.02.007

For peer review only - http://bmjopen.bmj.com/site/about/guidelines.xhtml

ISRCTN72038467

51. Aleman S, Rahbin N, Weiland O, et al. A risk for hepatocellular carcinoma persists long-term after sustained virologic response in patients with hepatitis C-associated liver cirrhosis. *Clin Infect Dis* 2013;57(2):230-6. doi: 10.1093/cid/cit234
52. van der Meer AJ, Veldt BJ, Feld JJ, et al. Association between sustained virological response and all-cause mortality among patients with chronic hepatitis C and advanced hepatic fibrosis. *JAMA* 2012;308(24):2584-93. doi: 10.1001/jama.2012.144878
53. Innes H, Hutchinson SJ, Obel N, et al. Liver mortality attributable to chronic hepatitis C virus infection in Denmark and Scotland--using spontaneous resolvers as the benchmark comparator. *Hepatology* 2016;63(5):1506-16. doi: 10.1002/hep.28458
54. Fraser H, Martin NK, Brummer-Korvenkontio H, et al. Model projections on the impact of HCV treatment in the prevention of HCV transmission among people who inject drugs in Europe. *J Hepatol* 2018;68(3):402-11. doi: 10.1016/j.jhep.2017.10.010 [published Online First: 2017/10/31]
55. Schulkind J, Stephens B, Ahmad F, et al. High response and re-infection rates among people who inject drugs treated for hepatitis C in a community needle and syringe programme. *Journal of Viral Hepatitis* 2018 doi: 10.1111/jvh.13035
56. MacArthur GJ, Minozzi S, Martin N, et al. Opiate substitution treatment and HIV transmission in people who inject drugs: systematic review and meta-analysis. *BMJ* 2012;345:e5945.
57. MacArthur GJ, van VE, Palmateer N, et al. Interventions to prevent HIV and Hepatitis C in people who inject drugs: A review of reviews to assess evidence of effectiveness. *IntJ Drug Policy* 2013 doi: S0955-3959(13)00116-3 [pii];10.1016/j.drugpo.2013.07.001 [doi]
58. Palmateer N, Hutchinson S, McAllister G, et al. Risk of transmission associated with sharing drug injecting paraphernalia: analysis of recent hepatitis C virus (HCV) infection using cross-sectional survey data. *J Viral Hepat* 2014;21(1):25-32. doi: 10.1111/jvh.12117
59. Palmateer N, Kimber J, Hickman M, et al. Evidence for the effectiveness of sterile injecting equipment provision in preventing hepatitis C and human immunodeficiency virus transmission among injecting drug users: a review of reviews. *Addiction* 2010;105(5):844-59. doi: ADD2888 [pii];10.1111/j.1360-0443.2009.02888.x [doi]
60. Presanis AM, Gill ON, Chadborn TR, et al. Insights into the rise in HIV infections, 2001 to 2008: a Bayesian synthesis of prevalence evidence. *AIDS* 2010;24(18):2849-58. doi: 10.1097/QAD.0b013e32834021ed [doi]
61. England PH. Hepatitis C in the UK 2016 report: Working towards its elimination as a major public health threat. In: Harris HE, ed. London: Public Health England, 2016.
62. Health Protection Scotland UotWoS, Glasgow Caledonian University and the West of Scotland Specialist Virology Centre. The Needle Exchange Surveillance Initiative: Prevalence of blood-borne viruses and injecting risk behaviours among people who inject drugs attending injecting equipment provision services in Scotland, 2008-09 to 2015-16. In: Scotland HP, ed. Glasgow, 2017.
63. Brant LJ, Hurrelle M, Balogun MA, et al. Sentinel laboratory surveillance of hepatitis C antibody testing in England: understanding the epidemiology of HCV infection. *Epidemiol Infect* 2007;135(3):417-26. doi: 10.1017/S0950268806006832
64. Brant LJ, Hurrelle M, Balogun MA, et al. Where are people being tested for anti-HCV in England? Results from sentinel laboratory surveillance. *J Viral Hepat* 2008;15(10):729-39. doi: 10.1111/j.1365-2893.2008.01000.x
65. Lattimore S, Irving W, Collins S, et al. Using surveillance data to determine treatment rates and outcomes for patients with chronic hepatitis C virus infection. *Hepatology* 2014;59(4):1343-50. doi: 10.1002/hep.26926
66. Treloar C, Newland J, Rance J, et al. Uptake and delivery of hepatitis C treatment in opiate substitution treatment: perceptions of clients and health professionals. *J Viral Hepat* 2010;17(12):839-44. doi: JVH1250 [pii];10.1111/j.1365-2893.2009.01250.x [doi]

For peer review only - http://bmjopen.bmj.com/site/about/guidelines.xhtml

ISRCTN72038467

67. Treloar C, Rance J, Dore GJ, et al. Barriers and facilitators for assessment and treatment of hepatitis C virus infection in the opioid substitution treatment setting: insights from the ETHOS study. *J Viral Hepat* 2014;21(8):560-7. doi: 10.1111/jvh.12183
68. Harris M, Rhodes T. Hepatitis C treatment access and uptake for people who inject drugs: a review mapping the role of social factors. *Harm Reduct J* 2013;10:7. doi: 10.1186/1477-7517-10-7
69. Swan D, Long J, Carr O, et al. Barriers to and facilitators of hepatitis C testing, management, and treatment among current and former injecting drug users: a qualitative exploration. *AIDS Patient Care STDS* 2010;24(12):753-62. doi: 10.1089/apc.2010.0142
70. Rhodes T, Harris M, Martin A. Negotiating access to medical treatment and the making of patient citizenship: the case of hepatitis C treatment. *Sociol Health Illn* 2013;35(7):1023-44. doi: 10.1111/1467-9566.12018
71. Harris M, Albers E, Swan T. The promise of treatment as prevention for hepatitis C: Meeting the needs of people who inject drugs? *Int J Drug Policy* 2015;26(10):963-9. doi: 10.1016/j.drugpo.2015.05.005
72. Rance J, Treloar C, Group ES. 'Not just methadone Tracy': transformations in service-user identity following the introduction of hepatitis C treatment into Australian opiate substitution settings. *Addiction* 2014;109(3):452-9.
73. Treloar C, Rance J, Backmund M. Understanding barriers to hepatitis C virus care and stigmatization from a social perspective. *Clin Infect Dis* 2013;57 Suppl 2:S51-5. doi: 10.1093/cid/cit263
74. Treloar C, Rance J, Bryant J, et al. Harm reduction workers and the challenge of engaging couples who inject drugs in hepatitis C prevention. *Drug Alcohol Depend* 2016;168:170-75. doi: 10.1016/j.drugalcdep.2016.09.010
75. Treloar C, Rance J, Bryant J, et al. Understanding decisions made about hepatitis C treatment by couples who inject drugs. *J Viral Hepat* 2016;23(2):89-95. doi: 10.1111/jvh.12451
76. Jack K, Islip N, Linsley P, et al. Prison officers' views about hepatitis C testing and treatment: A qualitative enquiry. *J Clin Nurs* 2016 doi: 10.1111/jocn.13489
77. Rich ZC, Chu C, Mao J, et al. Facilitators of HCV treatment adherence among people who inject drugs: a systematic qualitative review and implications for scale up of direct acting antivirals. *BMC Public Health* 2016;16:994. doi: 10.1186/s12889-016-3671-z
78. Clark JA, Gifford AL. Resolute efforts to cure hepatitis C: Understanding patients' reasons for completing antiviral treatment. *Health (London)* 2015;19(5):473-89. doi: 10.1177/1363459314555237
79. Batchelder AW, Peyser D, Nahvi S, et al. "Hepatitis C treatment turned me around:" Psychological and behavioral transformation related to hepatitis C treatment. *Drug Alcohol Depend* 2015;153:66-71. doi: 10.1016/j.drugalcdep.2015.06.007
80. Harris M. Managing expense and expectation in a treatment revolution: Problematizing prioritisation through an exploration of hepatitis C treatment 'benefit'. *Int J Drug Policy* 2017;47:161-68. doi: 10.1016/j.drugpo.2017.03.015 [published Online First: 2017/04/30]
81. Aspinall EJ, Hutchinson SJ, Janjua NZ, et al. Trends in mortality after diagnosis of hepatitis C virus infection: an international comparison and implications for monitoring the population impact of treatment. *J Hepatol* 2015;62(2):269-77. doi: 10.1016/j.jhep.2014.09.001
82. Aspinall EJ, Corson S, Doyle JS, et al. Treatment of hepatitis C virus infection among people who are actively injecting drugs: a systematic review and meta-analysis. *Clin Infect Dis* 2013;57 Suppl 2:S80-9. doi: 10.1093/cid/cit306
83. Judd A, Parry J, Hickman M, et al. Evaluation of a modified commercial assay in detecting antibody to hepatitis C virus in oral fluids and dried blood spots. *J Med Virol* 2003;71(1):49-55.

For peer review only - http://bmjopen.bmj.com/site/about/guidelines.xhtml

ISRCTN72038467

84. Bennett S, Gunson RN, McAllister GE, et al. Detection of hepatitis C virus RNA in dried blood spots. *J Clin Virol* 2012;54(2):106-09. doi: S1386-6532(12)00053-4 [pii];10.1016/j.jcv.2012.02.004 [doi]
85. Martin NK, Vickerman P, Brew IF, et al. Is increased hepatitis C virus case-finding combined with current or 8-week to 12-week direct-acting antiviral therapy cost-effective in UK prisons? A prevention benefit analysis. *Hepatology* 2016;63(6):1796-808. doi: 10.1002/hep.28497
86. McDonald SA, Innes HA, Hayes PC, et al. What is the impact of a country-wide scale-up in antiviral therapy on the characteristics and sustained viral response rates of patients treated for hepatitis C? *J Hepatol* 2015;62(2):262-8. doi: 10.1016/j.jhep.2014.08.046 [published Online First: 2014/09/10]
87. Ward Z, Platt L, Sweeney S, et al. Impact of current and scaled-up levels of hepatitis C prevention and treatment interventions for people who inject drugs in three UK settings-what is required to achieve the WHO's HCV elimination targets? *Addiction* 2018 doi: 10.1111/add.14217 [published Online First: 2018/05/19]
88. Brodersen KH, Gallusser F, Koehler J, et al. Inferring causal impact using Bayesian structural time-series models. 2015:247-74. doi: 10.1214/14-AOAS788
89. Scott S VH. Predicting the present with bayesian structural time series. *International Journal of Mathematical Modelling and Numerical Optimisation* 2014;2014(5):23.
90. Rance J, Newland J, Hopwood M, et al. The politics of place(ment): problematising the provision of hepatitis C treatment within opiate substitution clinics. *Soc Sci Med* 2012;74(2):245-53. doi: S0277-9536(11)00644-7 [pii];10.1016/j.socscimed.2011.10.003 [doi]
91. Lankenau SE, Sanders B, Hathazi D, et al. Recruiting and retaining mobile young injection drug users in a longitudinal study. *Subst Use Misuse* 2010;45(5):684-99. doi: 10.3109/10826081003594914
92. Stewart KE, Wright PB, Sims D, et al. The "translators": engaging former drug users as key research staff to design and implement a risk reduction program for rural cocaine users. *Subst Use Misuse* 2012;47(5):547-54. doi: 10.3109/10826084.2011.644379
93. Harris M. "Three in the Room": Embodiment, Disclosure, and Vulnerability in Qualitative Research. *Qual Health Res* 2015;25(12):1689-99. doi: 10.1177/1049732314566324
94. Braun V, Clarke V. Using thematic analysis in psychology. *Qualitative Research in Psychology* 2006;3(2):77-101. doi: 10.1191/1478088706qp063oa
95. Rogers EM. Diffusion of preventive innovations. *Addict Behav* 2002;27(6):989-93.
96. Cane J, O'Connor D, Michie S. Validation of the theoretical domains framework for use in behaviour change and implementation research. *Implement Sci* 2012;7:37. doi: 10.1186/1748-5908-7-37
97. Innes HA, Hutchinson SJ, Allen S, et al. Ranking predictors of a sustained viral response for patients with chronic hepatitis C treated with pegylated interferon and ribavirin in Scotland. *Eur J Gastroenterol Hepatol* 2012;24(6):646-55. doi: 10.1097/MEG.0b013e32835201a4
98. McDonald SA, Hutchinson SJ, Bird SM, et al. Excess morbidity in the hepatitis C-diagnosed population in Scotland, 1991-2006. *Epidemiol Infect* 2011;139(3):344-53. doi: 10.1017/S0950268810001421
99. McDonald SA, Hutchinson SJ, Bird SM, et al. A record-linkage study of the development of hepatocellular carcinoma in persons with hepatitis C infection in Scotland. *Br J Cancer* 2008;99(5):805-10.
100. McDonald SA, Hutchinson SJ, Bird SM, et al. A population-based record linkage study of mortality in hepatitis C-diagnosed persons with or without HIV coinfection in Scotland. *Stat Methods Med Res* 2009;18(3):271-83. doi: 10.1177/0962280208094690
101. Gao L, Dimitropoulou P, Robertson JR, et al. Risk-factors for methadone-specific deaths in Scotland's methadone-prescription clients between 2009 and 2013. *Drug Alcohol Depend* 2016;167:214-23. doi: 10.1016/j.drugalcdep.2016.08.627

For peer review only - http://bmjopen.bmj.com/site/about/guidelines.xhtml

ISRCTN72038467

102. Bird SM, Fischbacher CM, Graham L, et al. Impact of opioid substitution therapy for Scotland's prisoners on drug-related deaths soon after prisoner release. *Addiction* 2015;110(10):1617-24. doi: 10.1111/add.12969
103. Bird SM, Hutchinson SJ. Male drugs-related deaths in the fortnight after release from prison: Scotland, 1996-99. *Addiction* 2003;98(2):185-90.
104. Alvarez-Madrazo S, McTaggart S, Nangle C, et al. Data Resource Profile: The Scottish National Prescribing Information System (PIS). *Int J Epidemiol* 2016;45(3):714-15f. doi: 10.1093/ije/dyw060
105. Graham L, Fischbacher CM, Stockton D, et al. Understanding extreme mortality among prisoners: a national cohort study in Scotland using data linkage. *Eur J Public Health* 2015;25(5):879-85. doi: 10.1093/eurpub/cku252
106. Merrall EL, Bird SM, Hutchinson SJ. Mortality of those who attended drug services in Scotland 1996-2006: record-linkage study. *Int J Drug Policy* 2012;23(1):24-32. doi: 10.1016/j.drugpo.2011.05.010
107. McDonald SA, Hutchinson SJ, Bird SM, et al. Hospitalisation for an alcohol-related cause among injecting drug users in Scotland: increased risk following diagnosis with hepatitis C infection. *Int J Drug Policy* 2011;22(1):63-9. doi: 10.1016/j.drugpo.2010.04.003
108. de Vos AS, Prins M, Kretzschmar ME. Hepatitis C virus treatment as prevention among injecting drug users: who should we cure first? *Addiction* 2015;110(6):975-83. doi: 10.1111/add.12842
109. Boily MC, Lowndes CM, Vickerman P, et al. Evaluating large-scale HIV prevention interventions: study design for an integrated mathematical modelling approach. *Sex TransmInfect* 2007;83(7):582-89. doi: sti.2007.027516 [pii];10.1136/sti.2007.027516 [doi]
110. Boily MC, Pickles M, Lowndes CM, et al. Positive impact of a large-scale HIV prevention program among female sex workers and clients in Karnataka state, India. *AIDS* 2013 doi: 10.1097/QAD.0b013e32835fba81 [doi]
111. Hallett TB, Gregson S, Mugurungi O, et al. Assessing evidence for behaviour change affecting the course of HIV epidemics: a new mathematical modelling approach and application to data from Zimbabwe. *Epidemics* 2009;1(2):108-17. doi: 10.1016/j.epidem.2009.03.001
112. Pickles M, Boily MC, Vickerman P, et al. Assessment of the population-level effectiveness of the Avahan HIV-prevention programme in South India: a preplanned, causal-pathway-based modelling analysis. *Lancet Glob Health* 2013;1(5):e289-99. doi: 10.1016/S2214-109X(13)70083-4
113. Martin NK, Hickman M, Miners A, et al. Cost-effectiveness of HCV case-finding for people who inject drugs via dried blood spot testing in specialist addiction services and prisons. *BMJ open* 2013;3(8) doi: 10.1136/bmjopen-2013-003153
114. Martin NK, Miners A, Vickerman P. Assessing the cost-effectiveness of interventions aimed at promoting and offering hepatitis C testing to injecting drug users: An economic modelling report. London, 2012.
115. Martin NK, Pitcher AB, Vickerman P, et al. Optimal control of hepatitis C antiviral treatment programme delivery for prevention amongst a population of injecting drug users. *PLoS One* 2011;6(8):e22309. doi: 10.1371/journal.pone.0022309 [doi];PONE-D-10-01568 [pii]
116. Martin NK, Vickerman P, Miners A, et al. The cost-effectiveness of HCV antiviral treatment for injecting drug user populations. *Hepatology* 2012;55(1):49-57. doi: 10.1002/hep.24656 [doi]
117. Martin NK, Vickerman P, Miners A, et al. How cost-effective is hepatitis C virus treatment for people who inject drugs? *Journal of gastroenterology and hepatology* 2013;28(4):590-2. doi: 10.1111/jgh.12113
118. Kimber J, Copeland L, Hickman M, et al. Survival and cessation in injecting drug users: prospective observational study of outcomes and effect of opiate substitution treatment. *BMJ* 2010;341:c3172.
119. McDonald SA, Hutchinson SJ, Palmateer NE, et al. Decrease in health-related quality of life associated with awareness of hepatitis C virus infection among people who inject drugs in

For peer review only - http://bmjopen.bmj.com/site/about/guidelines.xhtml

ISRCTN72038467

Scotland. *JHepatol* 2013;58(3):460-66. doi: S0168-8278(12)00836-7 [pii];10.1016/j.jhep.2012.11.004 [doi]

120. Wright M, Grieve R, Roberts J, et al. Health benefits of antiviral therapy for mild chronic hepatitis C: randomised controlled trial and economic evaluation. *Health Technol Assess* 2006;10(21):1-113, iii. doi: 95-24-03 [pii]
121. Harrison GI, Murray K, Gore R, et al. The Hepatitis C Awareness Through to Treatment (HepCATT) study: Improving the cascade of care for hepatitis C virus-infected people who inject drugs in England. *Addiction* 2019 doi: 10.1111/add.14569 [published Online First: 2019/01/30]

For peer review only

For peer review only - http://bmjopen.bmj.com/site/about/guidelines.xhtml

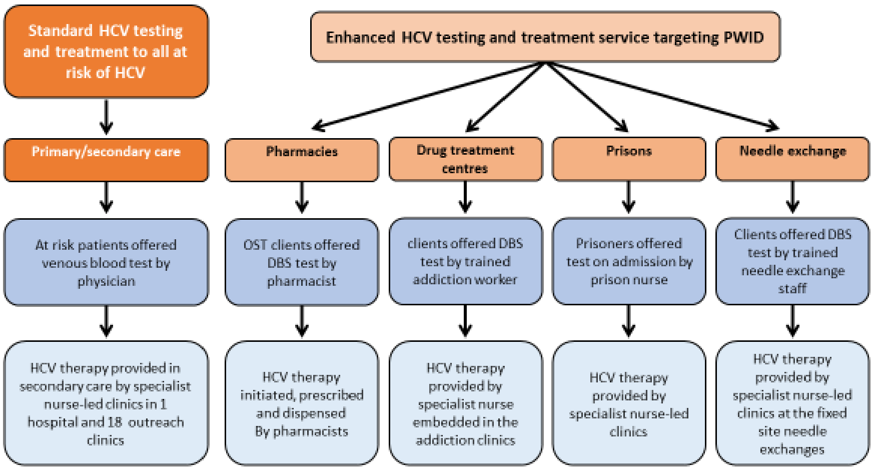

PWID defined as those who either (a) are currently injecting drugs, (b) have ever injected drugs and are currently on opioid substitute therapy, or (c) have ever injected drugs and are currently in prison

DBS: dried blood spot; OST: opioid substitution therapies; PWID: people who inject drugs

For peer review only

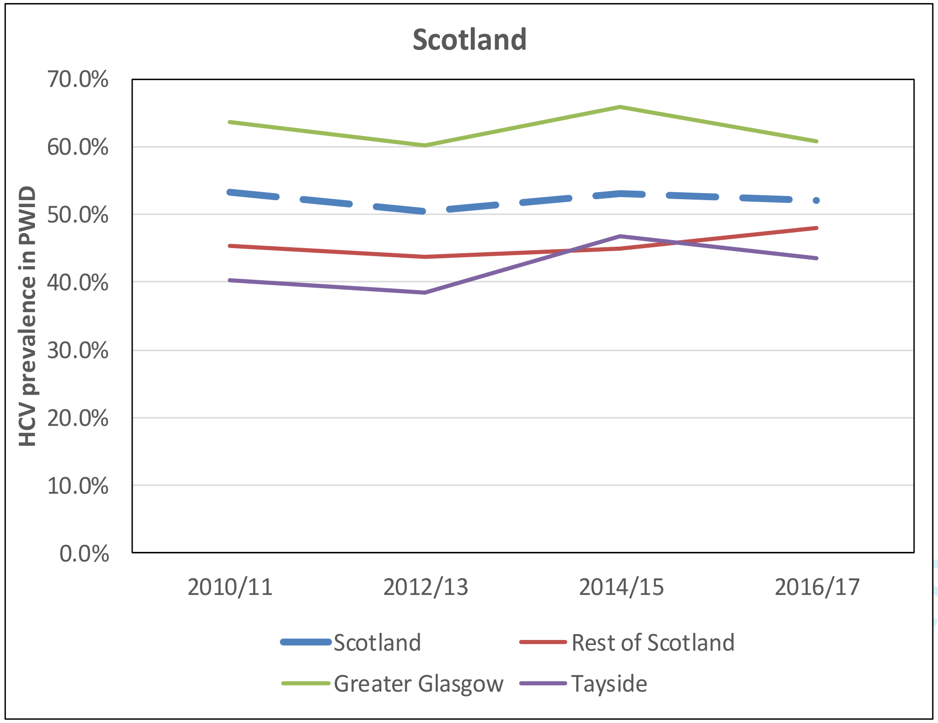
Scotland
70.0%
60.0%
50.0%
40.0%
30.0%
20.0%
10.0%
0.0%
HCV prevalence in PWID
2010/11
2012/13
2014/15
2016/17
Scotland
Rest of Scotland
Greater Glasgow
Tayside

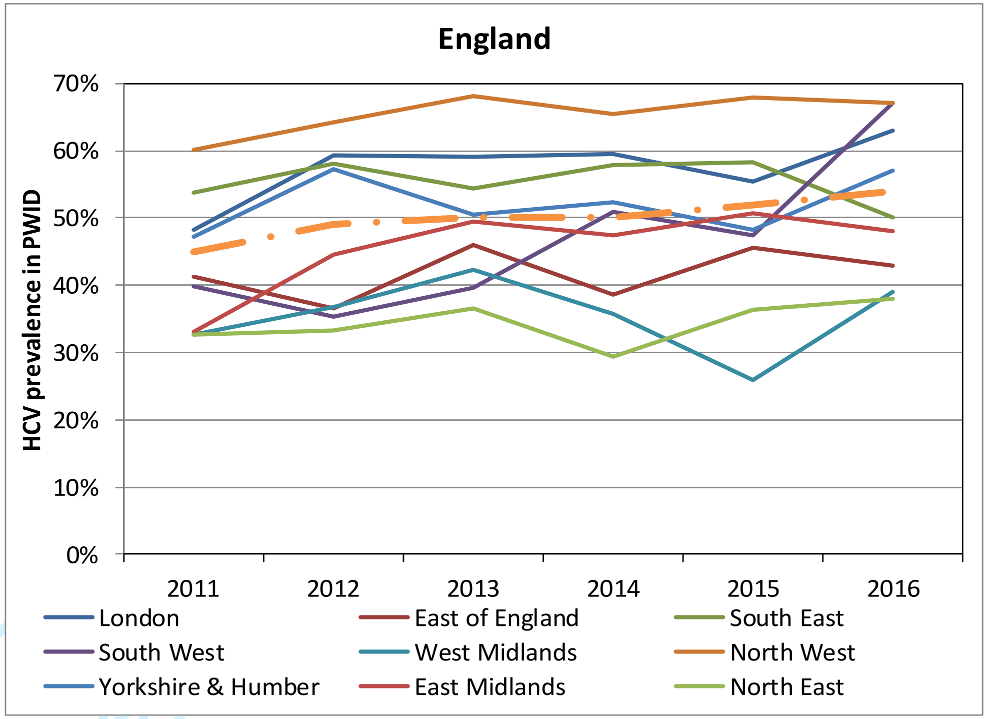
England
70%
60%
50%
40%
30%
20%
10%
0%
HCV prevalence in PWID
2011
2012
2013
2014
2015
2016
London
East of England
South East
South West
West Midlands
North West
Yorkshire & Humber
East Midlands
North East

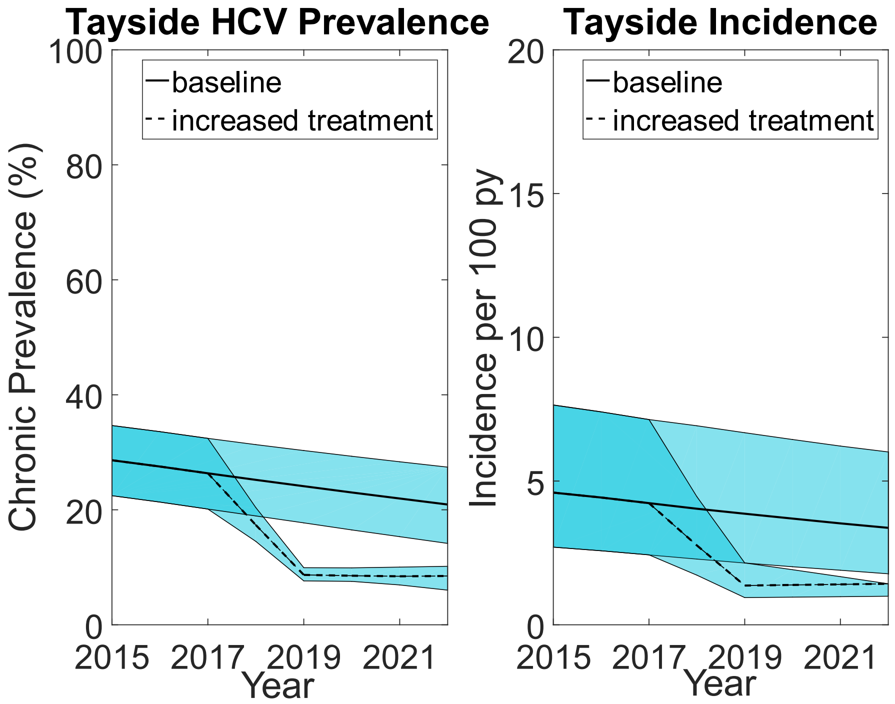

For peer review only - http://bmjopen.bmj.com/site/about/guidelines.xhtml

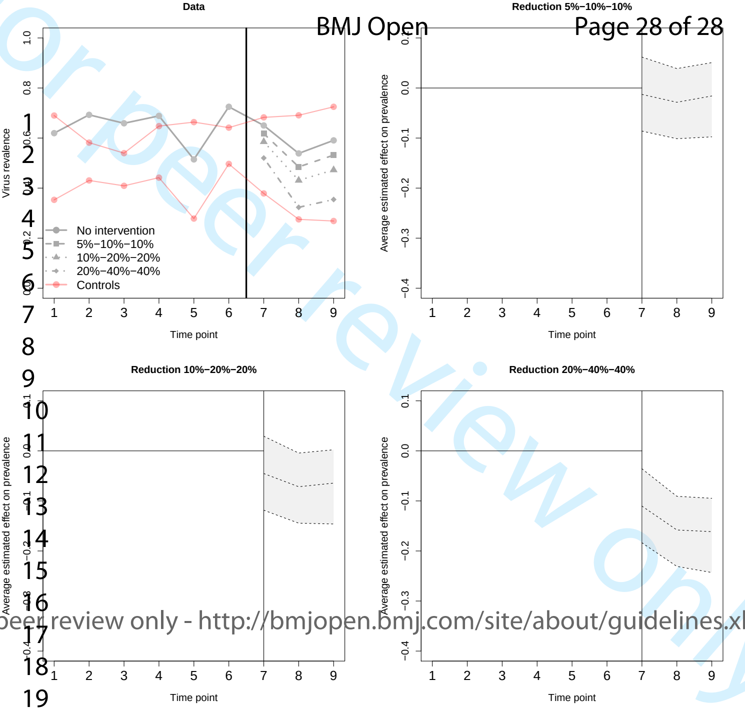

For peer review only

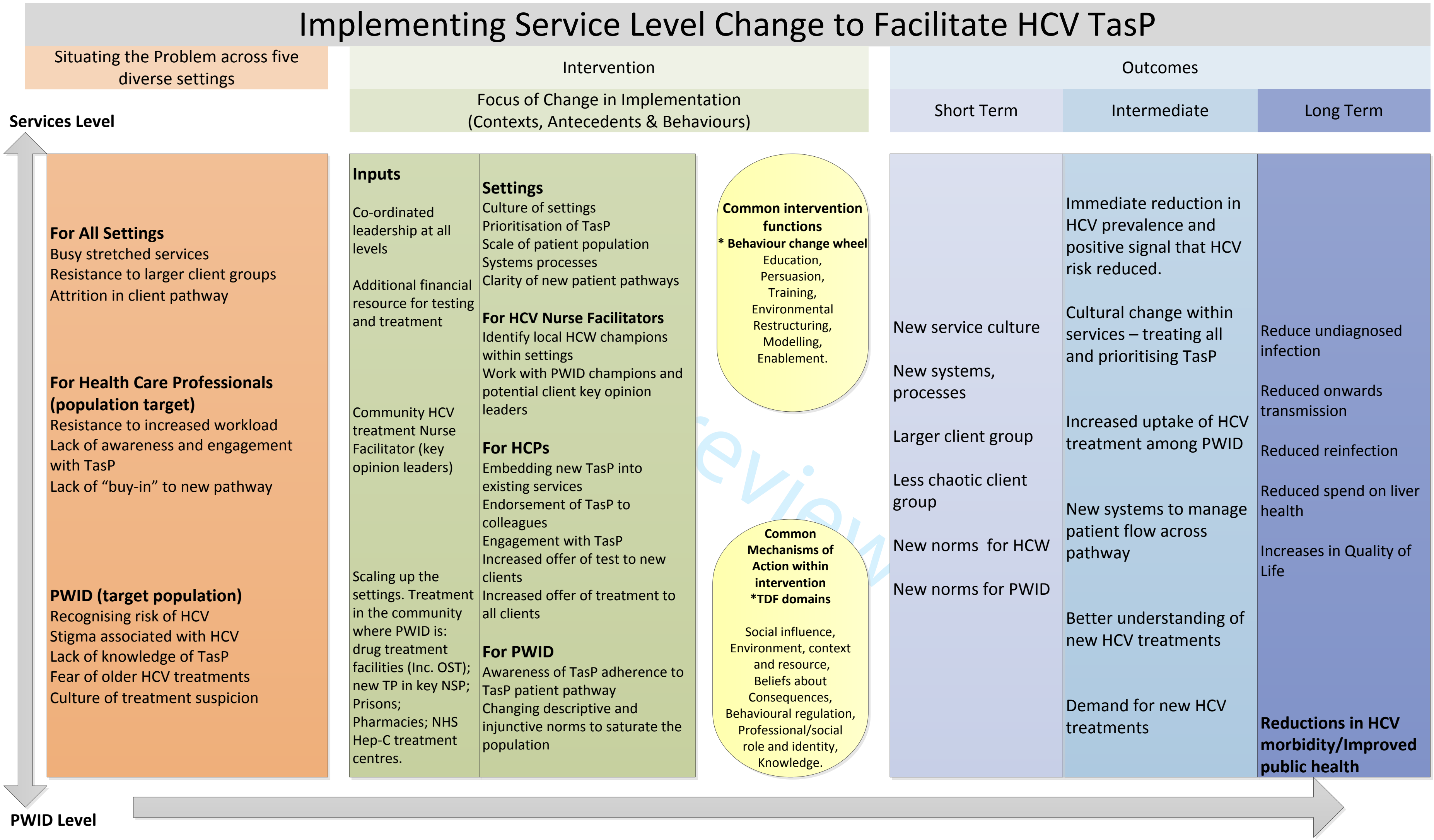
Implementing Service Level Change to Facilitate HCV TasP
Situating the Problem across five diverse settings
Intervention
Focus of Change in Implementation (Contexts, Antecedents & Behaviours)
Outcomes
Short Term
Intermediate
Long Term
Services Level
PWID Level
For All Settings
Busy stretched services
Resistance to larger client groups
Attrition in client pathway
For Health Care Professionals (population target)
Resistance to increased workload
Lack of awareness and engagement with TasP
Lack of "buy-in" to new pathway
PWID (target population)
Recognising risk of HCV
Stigma associated with HCV
Lack of knowledge of TasP
Fear of older HCV treatments
Culture of treatment suspicion
Inputs
Co-ordinated leadership at all levels
Additional financial resource for testing and treatment
Community HCV treatment Nurse Facilitator (key opinion leaders)
Scaling up the settings. Treatment in the community where PWID is: drug treatment facilities (Inc. OST); new TP in key NSP; Prisons; Pharmacies; NHS Hep-C treatment centres.
Settings
Culture of settings
Prioritisation of TasP
Scale of patient population
Systems processes
Clarity of new patient pathways
For HCV Nurse Facilitators
Identify local HCW champions within settings
Work with PWID champions and potential client key opinion leaders
For HCPs
Embedding new TasP into existing services
Endorsement of TasP to colleagues
Engagement with TasP
Increased offer of test to new clients
Increased offer of treatment to all clients
For PWID
Awareness of TasP adherence to TasP patient pathway
Changing descriptive and injunctive norms to saturate the population
Common intervention functions
* Behaviour change wheel
Education, Persuasion, Training, Environmental Restructuring, Modelling, Enablement.
Common Mechanisms of Action within intervention
*TDF domains
Social influence, Environment, context and resource, Beliefs about Consequences, Behavioural regulation, Professional/social role and identity, Knowledge.
New service culture
New systems, processes
Larger client group
Less chaotic client group
New norms for HCW
New norms for PWID
Immediate reduction in HCV prevalence and positive signal that HCV risk reduced.
Cultural change within services – treating all and prioritising TasP
Increased uptake of HCV treatment among PWID
New systems to manage patient flow across pathway
Better understanding of new HCV treatments
Demand for new HCV treatments
Reduce undiagnosed infection
Reduced onwards transmission
Reduced reinfection
Reduced spend on liver health
Increases in Quality of Life
Reductions in HCV morbidity/Improved public health

BMJ Open

Evaluating the Population Impact of Hepatitis C Direct Acting Antiviral Treatment as Prevention for People Who Inject Drugs (EPIToPe) – a natural experiment (Protocol)

Journal:	*BMJ Open*
Manuscript ID	bmjopen-2019-029538.R2
Article Type:	Protocol
Date Submitted by the Author:	25-Jul-2019

Complete List of Authors:	Hickman, M; Bristol<br>Dillon, John; University of Dundee Division of Cardiovascular and Diabetes Medicine, Liver Group<br>Elliott, Lawrie; Glasgow Caledonian University, Department of Nursing and Community Health<br>De Angelis, Daniela; MRC Biostatistics Unit<br>Vickerman, Peter; London School of Hygiene and Tropical Medicine; University of Bristol, School of Social and Community Medicine<br>Foster, Graham; Barts and The London School of Medicine<br>Donnan, Peter; University of Dundee, Dundee Epidemiology and Biostatistics Unit<br>Eriksen, Ann; Tayside Health Board<br>Flowers, Paul; Glasgow Caledonian University,<br>Goldberg, David; Health Protection Scotland<br>Hollingworth, William; University of Bristol, School of Social and Community Medicine<br>Ijaz, Samreen; Public Health England Centre for Infection<br>Liddell, David; Scottish Drugs Forum<br>Mandal, Sema; Public Health England, Immunisation, Hepatitis, Blood Safety and Countermeasures Response; National Institute for Health Research Health Protection Unit<br>Martin, Natasha; University of California, San Diego<br>Beer, Lewis; University of Dundee, Tayside Clinical Trials Unit<br>Drysdale, Kate; Barts Health NHS Trust<br>Fraser, Hannah; University of Bristol, School of Social and Community Medicine<br>Glass, Rachel; Public Health England, Immunisation, Hepatitis, Blood Safety and Countermeasures Response<br>Graham, Lesley; ISD Scotland<br>Gunson, Rory; Gartnavel General Hospital, West Of Scotland Specialist Virology Centre<br>Hamilton, Emma; Scottish Drug Forum<br>Harris, Helen; Public Health England, Immunisation, Hepatitis, Blood Safety and Countermeasures Response<br>Harris, Magdalena; London School of Hygiene and Tropical Medicine, Centre for Research on Drugs and Health Behaviours<br>Harris, Ross; Public Health England, Immunisation, Hepatitis, Blood Safety and Countermeasures Response<br>Heinsbroek, Ellen; Public Health England Colindale

	Hope, Vivian; Liverpool John Moores University<br>Horwood, Jeremy; University of Bristol, UK, School of Social and Community Medicine<br>Inglis, Sarah; University of Dundee, Division of Neuoscience; University of Dundee, Tayside Clinical Trials Unit<br>Innes, Hamish; Glasgow Caledonian University, School of health and life sciences<br>Lane, Athene; University of Bristol, School of Social and Community Medicine<br>Meadows, Jade; University of Bristol School of Social and Community Medicine<br>McAuley, Andrew; NHS Health Scotland<br>Metcalfe, Chris; University of Bristol, Social Medicine<br>Migchelsen, Stephanie; Public Health England, Immunisation, Hepatitis, Blood Safety and Countermeasures Response<br>Murray, Alex; Scottish Drug Forum<br>Myring, Gareth; University of Bristol School of Social and Community Medicine<br>Palmateer, Norah E.; Hlth Protect Scotland, Blood-borne viruses and sexually transmitted infections; Glasgow Caledonian University,<br>Presanis, Anne; Institute of Public Health, MRC Biostatistics Unit<br>Radley, Andrew; NHS Tayside, Directorate of Public Health; University of Dundee Division of Cardiovascular and Diabetes Medicine, Liver Group<br>Ramsay, Mary; Public Health England, immunisation; London School of Hygiene and Tropical Medicine, Epidemiology<br>Samartsidis, Pantelis; MRC Biostatistics Unit<br>Simmons, Ruth; Public Health England,<br>Sinka, Katy; Public Health England, Immunisation, Hepatitis, Blood Safety and Countermeasures Response<br>Vojt, Gabriele; Glasgow Caledonian University<br>Ward, Zoe; Bristol, Population Health Sciences, Bristol Medical School<br>Whiteley, David; Edinburgh Napier University<br>Yeung, Alan; Glasgow Caledonian University<br>Hutchinson, Sharon; Health Protection Scotland, ; Glasgow Caledonian University, School of Health and Life Sciences
**Primary Subject Heading**:	Public health
Secondary Subject Heading:	Addiction, Epidemiology, Gastroenterology and hepatology, Infectious diseases, Qualitative research
Keywords:	Hepatology < INTERNAL MEDICINE, INFECTIOUS DISEASES, Epidemiology < INFECTIOUS DISEASES, PUBLIC HEALTH, Public health < INFECTIOUS DISEASES, Infection control < INFECTIOUS DISEASES

SCHOLARONE™ Manuscripts

ISRCTN72038467

Evaluating the **P**opulation **I**mpact of Hepatitis C Direct Acting Antiviral **T**reatment as Prevention f**o**r **Pe**ople Who Inject Drugs (EPIToPe) – a natural experiment (Protocol)

Authors:

Hickman M[1], Dillon J[2], Elliott L[3], De Angelis D[4], Vickerman P[1], Foster G[5], Donnan PT[2], Eriksen A[6], Flowers P[3], Goldberg D[7], Hollingworth W[1], Ijaz S[8], Liddell D[9], Mandal S[8], Martin NK[10,1], Beer L[2] , Drysdale K[5], Fraser H[1], Glass R[8], Graham L[11], Gunson R[12], Hamilton E[9], Harris H[8], Harris M[13], Harris R[8], Heinsbroek E[8], Hope V[14], Horwood J[1], Inglis S K[2], Innes H[3], Lane A[1], Meadows, J[1], McAuley A[7], Metcalfe C[1], Migchelsen S[8], Murray, A[9], Myring G [1], Palmateer N[3], Presanis A[4], Radley A[2], Ramsay M[8], Samartsidis P[4], Simmons R[8], Sinka K[8], Vojt, G[3] , Ward Z[1], Whiteley D[15], Yeung, A[3] , Hutchinson S[3]

Correspondence to: Prof Matthew Hickman; Matthew.Hickman@bristol.ac.uk

Affiliations

1 Population Health Sciences, Bristol Medical School, University of Bristol

2 University of Dundee

3 Glasgow Caledonian University

4 MRC Biostatistics Unit, University of Cambridge

5 Barts Liver Centre, Queen Mary University of London

6 Tayside Health Board

7 Health Protection Scotland

8 Public Health England

9 Scottish Drug Forum

10 Division of Infectious Diseases and Global Public Health, University of California San Diego

11 Information Services Division, Scotland

12 West of Scotland Specialist Virology Centre, Glasgow Royal Infirmary

13 London School of Hygiene and Tropical Medicine

14 Liverpool John Moores University

15 Edinburgh Napier University

For peer review only

ISRCTN72038467

ABSTRACT

Introduction:

Hepatitis C Virus (HCV) is the second largest contributor to liver disease in the UK, with injecting drug use as the main risk factor among the estimated 200,000 people currently infected. Despite effective prevention interventions, chronic HCV prevalence remains at 40% among people who inject drugs (PWID). New Direct Acting Antiviral (DAA) HCV therapies combine high cure rates (>90%) and short treatment duration (8-12 weeks). Theoretical mathematical modelling evidence suggests HCV treatment scale-up can prevent transmission and substantially reduce HCV prevalence/incidence among PWID. Our primary aim is to generate empirical evidence on the effectiveness of HCV "Treatment as Prevention" (TasP) in PWID.

Methods and Analysis

We plan to establish a natural experiment with Tayside, Scotland, as a single intervention site where HCV care pathways are being expanded (including specialist drug treatment clinics, needle & syringe programmes (NSPs), pharmacies, and prison) and HCV treatment for PWID is being rapidly scaled-up. Other sites in Scotland and England will act as potential controls. Over two years from 2017/18, at least 500 PWID will be treated in Tayside, which simulation studies project will reduce chronic HCV prevalence among PWID by 62% (from 26% to 10%) and HCV incidence will fall by approximately 2/3 (from 4.2 per 100 person-years (p100py) to 1.4 p100py). Treatment response and re-infection rates will be monitored. We will conduct focus groups and interviews with service providers and patients that accept and decline treatment to identify barriers and facilitators in implementing TasP. We will conduct longitudinal interviews with up to 40 PWID to assess whether successful HCV treatment alters their perspectives on and engagement with drug treatment and recovery. Trained peer researchers will be involved in data collection and dissemination.

The primary outcome – chronic HCV prevalence in PWID – is measured using information from the Needle Exchange Surveillance Initiative (NESI) survey in Scotland and the Unlinked Anonymous Monitoring Programme (UAM) in England, conducted at least four times before and three times during and after the intervention. We will adapt Bayesian synthetic control methods (also called Causal Inference Models) to generate the cumulative impact of the intervention on chronic HCV prevalence and incidence. We will use a dynamic HCV transmission and economic model to evaluate the cost-effectiveness of the HCV TasP intervention, and to estimate the contribution of the scale up in HCV treatment to observed changes in HCV prevalence. Through the qualitative data we will systematically explore key mechanisms of TasP real world implementation from provider and patient perspectives to develop a manual for scaling up HCV treatment in other settings. We will compare qualitative accounts of drug treatment and recovery with a "virtual cohort" of PWID linking information on HCV treatment with Scottish Drug treatment databases to test whether DAA treatment improves drug treatment outcomes.

Ethics and Dissemination

Extending HCV community care pathways is covered by ethics (ERADICATE C, ISRCTN27564683, Super DOT C Trial clinicaltrials.gov:NCT02706223). Ethical approval for extra data collection from patients including health utilities and qualitative interviews has been granted (INSERT) and ISCRCTN registration has been completed (INSERT). Our findings will have direct NHS and patient relevance;

For peer review only - http://bmjopen.bmj.com/site/about/guidelines.xhtml

informing prioritization given to early HCV treatment for PWID. We will present findings to practitioners and policy makers, and support design of an evaluation of HCV TasP in England.

Strengths and limitations of this study

1. Our control sites in the rest of Scotland and England were not randomised so there will be confounding and uncertainty in the intervention effect estimates.
2. HCV treatment and prevention strategy in UK (and Europe) is evolving - motivated both by WHO "elimination targets" and falling drug prices – which may contaminate our controls.
3. However, our statistical models suggest that we should have sufficient power to detect an intervention effect and can model changes over time.
4. We will develop dynamic transmission and economic models that can estimate cost-effectiveness including the prevention benefit of this intervention.
5. We are conducting multiple nested qualitative studies and training and using peer researchers.

For peer review only

For peer review only - http://bmjopen.bmj.com/site/about/guidelines.xhtml

ISRCTN72038467

Introduction and Background:

Infection with Hepatitis C Virus (HCV) is a progressive disease that over 20-40 years can lead to liver cancer and premature death. HCV is the second largest contributor to liver disease in the UK and one of the few causes that is curable[1]. In the UK it is estimated that approximately 200,000 people are infected with HCV, over 85% of whom are people who inject or have injected drugs (PWID)[2-5]. Chronic HCV prevalence and incidence among PWID remains high in UK at 20-50% and 5 to 15 per 100 person- years respectively[4 6-18]. Prevention of HCV transmission among PWID is critical to long-term prevention of HCV related liver disease[19].

We have reviewed the effectiveness of traditional primary prevention against HCV –opioid substitution treatment (OST) and needle and syringe programmes (NSPs)[12 20-22]. Ongoing exposure to OST and high-coverage NSPs can reduce the risk of HCV transmission by 50-80%[12 22]. In Scotland HCV incidence among PWID decreased from approximately 14 to 6 per 100 person- years from 2008/09 to 2011/12 coinciding with the launch of the Scottish HCV strategy and action plan which incorporated scale-up of harm reduction interventions and HCV treatment[10 23]. We estimated that 60% of this decline could be attributed to the scale-up of OST and NSP during the action plan and that 1,400 HCV infections were averted by 2015[24]. However, there was no appreciable reduction in overall anti-HCV prevalence over this short period, and there is some suggestion that incidence has increased recently to ~10 per 100 person years (http://www.hps.scot.nhs.uk/resourcedocument.aspx?id=5863). HCV transmission models suggest that primary prevention through NSP and OST alone is insufficient to achieve substantial reductions (of the order of 40% or more within ten years) in HCV prevalence among PWID in the UK[25 26].

Prevention of hepatitis C disease and HCV transmission is now possible because highly effective, tolerable, short-course interferon-free direct acting antiviral therapies (DAAs) are available for all HCV genotypes with cure rates – defined as sustained virological response (SVR)- exceeding 90%[27-29]. We, and others, hypothesise that HCV Treatment scale-up for PWID, and resulting HCV Treatment as Prevention (TasP) could enhance other primary interventions and reduce HCV incidence and chronic prevalence to negligible levels (i.e. towards elimination as a major public health concern)[30-35]. TasP refers to the concept whereby future transmission is reduced by treating affected individuals[36 37]: in HIV TasP Antiretroviral Treatment reduces transmission because individuals have undetectable infection[38]; in HCV TasP people are cured so reducing opportunities for future transmission. World Health Organization (WHO) targets for HCV elimination, adopted by UK and other countries, aim to reduce HCV incidence by 80% and associated mortality by 65% by 2030[39 40 41 42 43 44].

Clinical guidelines in Europe and US changed from recommending prioritising HCV treatment to people with moderate to severe liver disease towards removing any restrictions and recommending that people at risk of transmission irrespective of fibrosis stage are offered treatment[45-49]. Cost-effectiveness models that incorporate the population prevention benefit suggest early treatment should be prioritised to PWID over other patient groups (unless chronic HCV prevalence and transmission is very high)[50]. There is direct evidence that SVR following HCV treatment reduces liver disease progression and mortality risk[51-53], but in two recent reviews we found no empirical evidence that HCV treatment scale-up has reduced chronic HCV prevalence and incidence in PWID populations[36 37]. In part this is because in most settings HCV treatment rates in PWID are too low and any changes generally too small to be detected, as we show in two studies of seven sites in UK[7]

For peer review only - http://bmjopen.bmj.com/site/about/guidelines.xhtml

ISRCTN72038467

and an extension to 11 sites in Europe[54]. Until very recently in the UK, the annual number of HCV DAA treatments was restricted -as drug costs could be expensive (>£10,000 per patient). There is the opportunity now to test whether scaling up HCV treatment will reduce chronic HCV prevalence and transmission among PWID[44].

In a pilot study ("Eradicate C") in Tayside we showed that we can increase HCV case-finding and engage and successfully treat PWID in the community[55]. Combining further studies on extending community HCV treatment pathways in Tayside and additional treatments provided by NHS Tayside and Scottish Government we can establish an immediate natural experiment (with Tayside as the intervention site and other sites in Scotland and England as controls) to test and generate UK empirical evidence on the and potential impact and cost-effectiveness of HCV "Treatment as Prevention" (TasP) in people who inject drugs (PWID). The UK is one of few countries worldwide to have an established nationwide surveillance system monitoring HCV infection among PWID [9 12 17 22 56-60]. This is undertaken through a series of cross-sectional voluntary anonymous surveys of PWID recruited at harm reduction services, referred to as the Unlinked Anonymous Monitoring Programme (UAM) in England and Wales and the Needle Exchange Surveillance Initiative (NESI) in Scotland[61 62]. In addition, the UK has established sentinel laboratory surveillance of HCV testing and national monitoring of HCV treatment [8 63-65]. The data collected in both UAM and NESI will be used to assess out outcome.

Alongside a natural experiment in Tayside, we will collect information to assess the treatment facilitators and barriers. Historically it has proven very hard to engage PWID in HCV treatment[66-69]. Some barriers to engagement, such as poor efficacy or fear of interferon treatment side-effects, may be ameliorated by DAA therapy. However, other barriers such as mistrust of health services, stigma, and competing priorities faced by PWID may persist. In addition, providers may be reticent to refer or provide HCV treatment to PWID due to concerns about adherence, reinfection and perceptions of treatment 'worth'[70 71]. It is expected that co-locating HCV treatment within existing services will reduce many system and provider level barriers to PWID accessing care[66-68 72-77]. However, this has not been tested in the context of community wide scale-up of interventions across multiple potential pathways. It is critical, therefore, that we understand how HCV TasP is embedded within the existing service landscape and incorporated into providers' professional roles.

Finally it has been hypothesised that successful HCV treatment in PWID may positively impact on understandings of self and identity and improve treatment of drug use disorders[71 72 78-80]. Accounts of 'transformative' outcomes extending beyond viral clearance alone include reference to reductions in drug and alcohol use, uptake of safer injecting practices, improved social relationships, enhanced sense of responsibility and self-worth. Hints of such collateral or indirect benefits are also found in quantitative studies reporting low re-infection rates and reductions in risky injecting behaviours among treated PWID[81 82]. We aim to test this hypothesis in our qualitative follow-up study and compare the findings to quantitative data generated from a virtual cohort.

For peer review only - http://bmjopen.bmj.com/site/about/guidelines.xhtml

ISRCTN72038467

Methods and analysis:

Study design

Our intention is to create and conduct a mixed methods study, including qualitative studies and economic evaluation, of a natural experiment of HCV Treatment as Prevention (TasP) among People who Inject Drugs (PWID). We also will develop methods for evaluating HCV TasP.

Methods

Scaling-up HCV treatment

The **intervention** comprises the scale-up of HCV treatment in People Who Inject Drugs (PWID) which has started early in Tayside. . By combining support from Scottish Government, National Health Board Tayside (NHS Tayside) and industry (MSD, Gilead, BMS) we can deliver rapid intensive scale-up of HCV treatments for PWID (comprising an extra 400 HCV treatments, a 3.5-fold increase from treatments for PWID prior to April 2017, see sample size below). We have developed multiple integrated community HCV care pathways, including novel care pathways in pharmacies, a low threshold NSP, drug treatment services and prisons (see Figure 1). Our diagnostic pathways make extensive use of dried blood spot (DBS) testing for diagnosis of HCV antibody and chronic HCV with subsequent conventional laboratory testing in preparation for treatment (viral load, liver function and Fib4 fibrosis score)[83-85].

Study population

Our intervention is delivered and measured at the population level – which we have created by combining several individual studies and treatment pathways as shown in Figure 1 (see ethics section below for the individual studies). We gained ethical approval East of Scotland Research Ethics Service REC 1 (ref: 18/ES/0128) to ask patients for permission to be recruited into the qualitative study (below) and extended clinical and behavioural drug history and data on health utilities (EQ5D-5L) at onset of treatment, during treatment and after the end of treatment.

Community HCV specialist nurses (3.5 FTE) coordinate and deliver case-finding and treatment across the pathways in Tayside (Figure 1).

Figure 1: Overview of HCV testing and treatment pathways for the PWID population in NHS Tayside.

The region of Tayside co-localises to NHS Tayside which is the provider of health care to a geographical area of 2,903 sq mi (7519 km2) including the cities of Dundee and Perth and the counties of Angus and Perth & Kinross, situated in the east of Scotland with a population of 416,000. It is a mixture of urban and rural environments with some of the most affluent and most deprived areas in Scotland. It is therefore a representative microcosm of many areas in the UK.

For peer review only - http://bmjopen.bmj.com/site/about/guidelines.xhtml

ISRCTN72038467

HCV treatment

Apart from expansion of community HCV care pathways, no new clinical procedures will be investigated and all PWID with chronic HCV will be offered oral DAA HCV treatment compliant with the Scottish clinical guidelines (https://www.hps.scot.nhs.uk/resourcedocument.aspx?id=6621).

As per local standard of care, participants will be offered appropriate harm reduction advice.

Standard care for patients is to test for SVR at 12 weeks after end of treatment with patients being recommended for annual follow-up if at risk of re-infection. Specialist nurses concentrate on building a good relationship with the participant to ensure that they do return for follow-up appointments. Health Protection Scotland collates national public health surveillance data on the number, characteristics and response of patients initiated onto HCV therapy, through Clinical Databases installed in 17 specialist HCV treatment centres, across Scotland[41 86]. A similar system also is available in England.

HCV surveillance and Intervention Outcome (Chronic HCV in PWID)

The **outcome** is chronic HCV prevalence (HCV viraemia as measured by HCV PCR) among PWID in the community (not just in the patients who undergo HCV treatment). Prevalence will be monitored using the NESI and UAM surveys, as detailed below. During 2017-22, three waves of data collection for NESI (n=7,500) and five to six for UAM (n=17,000 in England) will measure this outcome.

In our pre-intervention period from 2010/11 to 2016 there have been four NESI surveys in Scotland (n=10,000 participants in total) and six UAM surveys in England (n=16,000 in total), which have involved the collection of DBS linked to questionnaire data. Participants are recruited at sentinel sites by a team of trained interviewers in Scotland (at over 100 NSP sites) and by agency staff in over 60 low-threshold drug agencies across England[58 61]. Participants complete a short questionnaire, with common questions across UAM and NESI, on demographics, injecting behaviour and service utilisation, and importantly (in relation to quantifying the intervention effect) both survey approaches have remained consistent over time.

The DBS samples collected in NESI and UAM have all been tested for HCV antibody, using the same methods (where sensitivity and specificity of the assay on DBS are close to 100%)[83 84], and illustrate that antibody prevalence (ever infection) has remained relatively stable among PWID during this time (Figure 2). PCR positivity among antibody positive samples is used to determine chronic infection.

All NESI and UAM samples will be tested for HCV antibody and RNA PCR to assess the impact of HCV therapy scale-up – which is critical as trends in chronic infection and antibody status will diverge as more people are cured. In addition, we will undertake RNA PCR testing of all historical samples that were HCV antibody positive shown in Figure 2 so that we can measure chronic HCV prevalence among PWID pre-, as well as post-, intervention for analysis (below)

Figure 2: Trends in HCV antibody prevalence among PWID in Scotland and England 2010/11-16

Data on HCV PCR positivity among antibody negative samples identify recent infections and is used to estimate HCV incidence – which has fluctuated between 5-10 infections per 100 person years across the UK during the last five years[61]. We will also estimate HCV incidence from our transmission dynamic models[24 54].

For peer review only

For peer review only - http://bmjopen.bmj.com/site/about/guidelines.xhtml

ISRCTN72038467

Sample size, Power, and Estimating Intervention Effect

We updated estimates of the prevalence of PWID in Tayside[5] which suggest there are 2,760 (95% Credible Interval, CrI 2,360-3,170) PWID either currently injecting and/or in OST. NESI data suggest that approximately 30% have chronic HCV and over 75% of PWID with chronic HCV have been diagnosed. Prior to 2017 approximately 66 PWID were treated annually. From April 2017 we plan to treat at least 500 PWID in Tayside over 2 years (as a result of expanded community care pathways shown in Figure 1 and extra HCV treatments provided by NHS, Scottish Government and Industry funding). Adapting a transmission dynamic model that has been used in Tayside,[87] we hypothesize that within two years chronic HCV prevalence among PWID will reduce by approximately 62% from 26% (95% CrI 20-32) to at least 10% and chronic HCV incidence will fall by approximately 2/3s from 4.2 (95% CrI 2.4-7.1) per 100 person-years (p100py) to 1.4 (95%CrI 1.0 – 1.4) p100py (as shown in Figure 3). Modelling also suggests that maintaining these reductions after 2019 will require less than 40 treatments per year.

Figure 3: Projected chronic HCV prevalence and incidence among PWID in Tayside with and without the intervention. Blue shaded area denotes the 95% credibility intervals of the model projections with and without the intervention

We will adapt synthetic control methods or Causal Impact Model as proposed by Brodersen and colleagues[88 89].

We have performed simulation studies to test power and evaluate the utility of the CIM assuming information on chronic HCV prevalence among PWID (shown in Figure 4). Provided trends in the chronic HCV prevalence in the pre-intervention period are relatively stable (which is the case) there will be sufficient power to detect the projected reduction in chronic prevalence. For example, in Figure 4d we see that for a prevalence reduction of 40% by year 2-3 the credible intervals of the estimated cumulative effect (cumulative drop in prevalence) exclude zero, correctly identifying evidence of a successful intervention. Whereas a cumulative reduction of <20% is unlikely to be detected.

Figure 4: Causal Impact Synthetic Control Method (CIM) simulation and estimated intervention effects and 95% Credible Intervals for a range of assumed effects.

Footnote:- Illustration of CIM. First subplot shows a single dataset, where solid lines represent the simulated prevalence in the absence of the intervention, and the dashed lines represent the outcome of treated site in the post intervention period under different intervention magnitude scenarios. For each one of the three scenarios, we calculate the estimated average intervention effect along with credible intervals. These are shown in Subplots 2-4. We see that as the effect increases, the intervals tend to move away for zero. However, the intervention effect only becomes significant in scenario 3, where zero is not included in any of the post-intervention time points.

For peer review only - http://bmjopen.bmj.com/site/about/guidelines.xhtml

ISRCTN72038467

Qualitative Studies

Understanding the barriers and facilitators to scaling-up community-based HCV treatment

The qualitative study design has two distinct arms focusing on the intervention providers, and the intervention recipients.

Intervention providers

A purposive sample of 30 intervention providers, comprising nursing leads and key individuals from collaborating organisations will be approached directly by the lead hepatitis nurse. Seven focus groups will be convened according to professional role and locality:

- HCV healthcare specialists (nurses and physicians)
- Community pharmacists
- Prison staff (both healthcare and security)
- 'Drug workers' (from OST and NSP services)

Each focus group will consist of a maximum of six individuals and ideally comprise multi-agency mixed groups. Individual interviews by telephone will be offered for those hesitant to join a group (estimate 10 interviews). Topic guides informed by previous work in this area[66 68 76 90] will facilitate group discussion.

Intervention recipients – cross-sectional and longitudinal

The intervention recipient arm of the study will comprise both cross-sectional and longitudinal elements. A cross-sectional approach will be employed to recruit 6-10 participants who do not take up the offer of treatment. These individuals will be recruited through the treatment pathways or through our peer researcher networks. The longitudinal element will follow a cohort of up to 40 individuals recruited following their course of HCV treatment. These individuals will be purposively sampled from the existing services in which HCV TasP has been embedded (i.e. pharmacy, prison and drug service), and then followed-up at one year post-treatment (with 70% expected to be followed-up)[91]. We aim to recruit women as well as men, younger and older people; those treated previously and first time; those injecting and not injecting at treatment onset. Follow-up interviews will explore collateral effects of HCV TasP including outcomes pertaining to drug use and injecting practices (secondary outcome below).

Participants will be recruited by hepatitis nurses or other clinical staff in Tayside and the face-to-face semi-structured interview will be conducted by peer-researchers, trained and guided by experienced qualitative researchers. Dr Magdalena Harris explains the importance of the use of peer researchers within the context of EPIToPe: https://www.youtube.com/watch?v=9ZZo3fKOXlg

[92 93]. The Scottish Drugs Forum (SDF) works with a group of Tayside peer-researchers with lived experience of injecting. Peer-researchers will receive study-orientated training and be provided with ongoing support to co-produce data and contribute to study outputs. A £20 shopping voucher will be offered to all interviewees except those in prison (Scottish prison service ethics did not permit thank you vouchers to prison participants).

For peer review only - http://bmjopen.bmj.com/site/about/guidelines.xhtml

ISRCTN72038467

Qualitative Data Analysis

Interviews and focus-groups will be audio-recorded using encrypted digital voice recorders, transcribed verbatim and anonymised. *Nvivo* v.10 software will be used to code and manage qualitative data. First level analysis will be deductive, guided by the research questions, and peer researchers will be consulted for input and feedback during the analytical process[94]. A constant comparison method will be used to develop the thematic analysis and will reflect diverging and converging narratives, for example, across groups of intervention recipients at different time points in the treatment pathway, or between groups of intervention providers[94]. The findings will be contextualised in the relevant theoretical perspectives which may include the diffusion of preventive innovations (staff) or social norms and values that might underpin health behaviour (recipients)[95 96]. We will assess TasP both from the providers' perspective and from patients' perspective including those who refuse treatment.

We will use the findings iteratively to update the HCV TasP logic model shown in Figure 5. Our qualitative data will be used to generate a manual of an optimal intervention for other sites in UK. In previous examples, such as [https://www.youtube.com/channel/UCBV8smLmkOQVT9D0OR-md1g/videos] we have used the Behaviour Change Wheel [96] as the framework to retrospectively analyse the success and failure of implementation within Tayside and then prospectively to formulate the optimal implementation intervention.

Figure 5: Preliminary Logic Model HCV Treatment as Prevention (EPIToPe)

Mixed Method Study on drug use outcomes: OST retention, drug overdose, recovery, and social transformation

Health Protection Scotland (HPS) link data on diagnostic HCV tests in the four largest Scottish NHS boards (including Tayside)[8] and all persons undergoing HCV treatment in the Scottish HCV Clinical database[97] which are also linked with other databases (including deaths, hospitalisations and drug treatment)[8 42 98-100] and from 2018 Scotland's Prescribing Information System (PIS) which holds data on OST and NHS prison health database (Prison Vision)[101-105]. PWID attending drug services who were HCV diagnosed, compared to those who were not, are at increased risk of drug-related and other cause-specific morbidity/mortality[106 107]. Thus, we will create a virtual cohort of chronic HCV infected PWID (estimated to involve at least 600 individuals from Tayside and 3,000 from elsewhere) and through linkage identify those who have been treated and attained SVR with those who have not. We will assess and compare the following outcomes:- retention in drug treatment (determined through linkage to drug treatment and prescribing databases), drug- and alcohol- related morbidity/mortality (through linkage to all hospital admission and mortality databases), and other markers of relapse (through linkage to prisons database).

For peer review only - http://bmjopen.bmj.com/site/about/guidelines.xhtml

ISRCTN72038467

Economic and impact evaluation

Infectious disease models can test the extent to which observed changes in disease transmission can be attributed to specific interventions,[108-112] and assess cost-effectiveness of interventions that avert secondary infections, i.e. have a population prevention benefit [50 85 113-117]. We will update and adapt a transmission model of HCV among PWID in Scotland and Tayside to model the impact of the HCV treatment intervention based on historical trends and new observations collected as part of this programme[39 87]. We will stratify the PWID population into current (injected in the previous year) and temporarily ceased (in OST and not injected in the previous year); as well as by duration of injecting (< 3 years, 3 to 9 years, 10+ years since onset), prevention intervention exposure (OST and/or high coverage NSP), and intervention settings for testing and treatment. We will use Approximate Bayesian Computation to calibrate the model to pre-intervention trends in chronic HCV prevalence and incidence among PWID in Tayside. The model will simulate the impact of observed rates of HCV treatment and cure rates for the intervention period, also incorporating any changes in the coverage of OST and NSP and injecting risk behaviours.

We will test consistency between the model impact projections and observed changes in HCV chronic prevalence and incidence from Tayside to disentangle the impact of HCV TasP from other interventions (OST/NSP) or epidemiological changes, and predict the impact of the TasP on number of HCV infections averted. If they are not consistent then alternative evidence-based hypotheses will be tested for why the model projects a different impact and the best fitting models will then be used to project the impact of the intervention. This will be assessed compared to two alternative counterfactuals where treatment rates are either at pre-scale-up levels in Tayside or at the average level achieved in other UK sites over the scale-up period. The impact of any changes in OST and NSP coverage will also be assessed to determine the contribution of those changes on observed effects. Impact will be assessed in terms of the relative decrease in prevalence and incidence, as well as the number and percent of infections averted in the intervention model projections compared to each counterfactual over different time frames. These model projections can also be taken forward to evaluate the possible impact of the intervention over next 5 or 10 years.

We will evaluate the cost-effectiveness of the intervention (HCV treatment scale-up) compared to status quo (expected rate of HCV case-finding and treatment among PWID in the rest of the UK) from a health care provider (NHS) perspective, with the cost-effectiveness of the different settings where case-finding occurs also being assessed. The cost-effectiveness (CE) model will be based on the same dynamic impact model, adapted to include HCV disease progression stages and tracking of health outcomes among PWID after cessation of injecting[50]. The economic evaluation will incorporate both individual benefits of HCV treatment (on disease progression) as well as population benefits (on HCV transmission). We will calculate the total number of infections and deaths over a 50-year time horizon for the intervention and counterfactual scenario and estimate the costs and quality-adjusted life years (QALYs) based on the number of individuals in each disease stage per year in the model. We will discount all future costs and QALYs at 3.5% (NICE guidelines https://www.nice.org.uk/process/pmg9/resources/guide-to-the-methods-of-technology-appraisal-2013-pdf-2007975843781). Probabilistic sensitivity analyses will be used to estimate the parametric uncertainty in the impact and cost projections. Cost-effectiveness results will be expressed in terms of incremental cost-effectiveness ratios (ICERs) and net monetary benefits (NMB) estimated using

For peer review only - http://bmjopen.bmj.com/site/about/guidelines.xhtml

ISRCTN72038467

NICE thresholds (£20,000 and £30,000 per QALY). We will plot cost-effectiveness acceptability curves to determine the probability of the intervention being cost-effective compared to different willingness-to-pay thresholds. Analyses of covariance (ANCOVA) methods will be used to summarize the proportion of the variability in the incremental costs and QALYs explained by uncertainty in different input parameters. Univariate sensitivity analyses will consider the effect of changes in important parameters such as time horizon, treatment cost and discount rate.

We focus on the incremental or additional resource costs associated with the intervention in Tayside. These costs, in part based on our earlier work for other studies, will include such things as the nurse time spent on intervention related activities (training other staff to offer HCV testing and treatment referral) as well as additional HCV testing and treatment costs, any additional OST costs due to HCV testing or treatment, and other staff time at the NSP, drug treatment centres and prisons involved with the intervention. Most of the incremental costs can be defined as variable (driven by extra nurse time and HCV testing/treatment costs). NHS HCV care costs and health utilities will be attached to each disease stage, based primarily on previous syntheses and models, which assume that PWID have a lower QoL than non-PWID of a similar age, gender and liver disease stage[118-120]. Additional data using the EQ-5D-5L tool during this study will generate new health utility data on the QoL amongst PWID before and after DAA treatment.

Patient and Public Involvement

Patient and Public Involvement (PPI) was led by the Hepatitis C Trust and supported by qualitative research assessing barriers and facilitators to HCV treatment access (led by Magdalena Harris). The Scottish Drug Forum (SDF) were also actively involved in the development of EPIToPe. The input from PPI groups has influenced the design of care pathways and has ensured that peer research is an essential element of the qualitative strand of EPIToPe.

A pilot NIHR funded study in England (HEPCAT) responding to NICE Guidance on Hepatitis Case Finding was co-designed with Hepatitis C trust. It showed that Hepatitis C Facilitators and peer support networks can increase the uptake of HCV case-finding and HCV treatment readiness in addiction services. This pilot study and our studies in Dundee/Tayside will influence how HCV treatment can be scaled up in England and our proposed evaluation HCV treatment as prevention.

Peer researchers will be trained to conduct the longitudinal study with PWID treated for HCV and will be involved and contribute to the analysis of the findings. Peer researchers and SDF will be members of the project management group and steering committee.

Dissemination events will be held in Dundee to discuss and present the findings from the qualitative studies with patient groups and services. These will be facilitated by SDF to support active contribution from our peer researchers. The study findings will be summarised and promoted through SDF website, social media platforms and through their sector-wide conferences in Scotland. Hepatitis Scotland, who are hosted within SDF, together with patient and public groups in England

For peer review only - http://bmjopen.bmj.com/site/about/guidelines.xhtml

will take an active role in the wider national and international dissemination of the research, it's translation into patient meaningful materials and its integration into a national policy context. The research will also be promoted via Hepatitis C Trust and Public Health England.

Discussion

Strengths and limitations of this study

Several limitations arise from the "natural experiment" design as our intervention and controls were not randomised. In the UK and many other countries there is no longer sufficient equipoise in clinicians and policymakers – given WHO and national strategies on HCV "elimination" - to mount an RCT of HCV Treatment as Prevention. As a result, there will be confounding and additional uncertainty in the measurement of the intervention effect. However, we consider that a natural experiment and use of synthetic control methods to be a more robust design than simple before and after studies. Our preliminary simulation work also suggests that we should have sufficient power to detect the large intervention effect that is planned.

We know also that HCV treatment and prevention strategy in UK (and Europe) is evolving - motivated both by WHO "elimination targets" and falling drug prices – and our control sites in Scotland and England may increase HCV treatment rates earlier than expected. This will complicate the analyses a little and potentially dilute the intervention effect. We are confident that we can adapt the synthetic control methods to take account of changes over time – and that because Tayside has started so early in scaling up HCV treatment that we will have time to detect a difference in the outcome.

The lack of randomised controls means that we have to generate the counterfactual of "no HCV treatment scale-up" through our HCV transmission model so that we can subsequently estimate cost-effectiveness of the intervention in Tayside. This is not ideal but has become standard practice in economic models of novel HCV treatment interventions – and we are involved with the modelling of HCV treatment pathways through homeless centres, prison, A&E, pharmacies, specialist drug clinics, and NSPs (P Vickerman personal communication and e.g. [55 85 121]. We know also, however, that the benefit in terms of additional Quality of Life Years and averted HCV infections accrues and occurs over a prolonged period [50]. It is more critical for any economic evaluation of HCV interventions in PWID that a dynamic model is used so that the prevention benefit (in terms of HCV infections averted) is correctly accounted for.

We are using peer researchers in the qualitative arm of patients' perspectives on the intervention and on the impact of HCV treatment on addiction outcomes. This is novel but adds additional challenges to obtaining NHS passports and ensuring data quality across the interviews and interviewees. We are also intending to support peers in analysis and interpretation of the findings which we believe has not been done before. We have trained the interviewers and will be monitoring their performance of the interviewers to ensure consistent study quality – and will replace peers with our qualitative researcher if required.

Future Study: Natural experiment of TasP in England

For peer review only - http://bmjopen.bmj.com/site/about/guidelines.xhtml

ISRCTN72038467

In England HCV treatment is delivered through 22 operational delivery networks (ODNs). NHS England's HCV strategy (2016-2019) prioritised 10,000 patients per year in line with the declared priorities of the network which could (and in many cases did) include people who use drugs at risk of transmission [44]. In October 2018 it is anticipated that a new procurement deal will substantially increase the number of patients who can access DAAs and this will enable 'trace and treat' options to be introduced. We will use the first part of EPIToPe including the manual generated by the qualitative study, enhancements to historical and ongoing surveillance of chronic HCV in PWID, infectious disease models, and methodological developments of causal impact model, to co-design with ODN leads a natural experiment of HCV TasP in England.

Authors' contributions:

All authors contributed to editing of the manuscript.

M Hickman (MH) and S Hutchinson (SH) are co-PIs of EPIToPe and prepared first draft of the manuscript.

J Dillon (JD) leads intervention scale-up in Tayside in collaboration with Tayside CTU (L Beer (LB), PT Donnan (PTD), S Inglis (SI), Andrew Radley (AR) and A Eriksen (AE)).

L Elliot (LE) leads qualitative component of EPIToPe in collaboration with Scottish Drug Forum (D Liddell (DL), E Hamilton (EH) and A Murray (AM)) and support from M Harris (MHa), G Vojt (GV) and D Whitely (DW) on qualitative research and training of peer support workers, and P Flowers (PF) on behavioural science.

D De Angelis (DA) leads synthetic control estimation and multiple parameter evidence synthesis in collaboration with P Samartsidis (PS), R Harris (RA), A Presanis (AP), and N Martin (NM).

P Vickerman (PV) leads dynamic impact and economic modelling in collaboration with NM, Z Ward (ZW), H Fraser (HF) with health economics led by W Hollingworth (WH), with G Myring (GM), as part of Bristol Randomised Trial Collaboration (BRTC) with advice on trial design from J Horwood (JH), C Metcalfe (CM) and A Lane (AL).

G Foster (GF) is leading design of evaluation in England based on EPIToPe with support from BRTC and K Drysdale

SH is leading on outcome measurement in Scotland with Health Protection Scotland (D Goldberg (DG), A McAuley (AMc)), and in collaboration with L Graham (LG) from ISD, R Gunson (RG), H Innes (HI), N Palmateer (NP) and A Yeung (AY).

S Mandal (SM) and S Ijaz (SI) are leading on outcome measurement in England with R Glass (RG), H Harris (HH), E Heinsbroek (EH), V Hope (VH), S Migchelsen (SM), M Ramsay (MR), R Simmons (RS), K Sinka (SK).

J Meadows (JM) is the Programme Manager.

Funding statement: 'This paper presents independent research funded by the National Institute for Health Research (NIHR) under its Programme Grants for Applied Research Programme (Reference Number RP-PG-0616-20008). The views expressed are those of the author(s) and not necessarily those of the NHS, the NIHR or the Department of Health.'

For peer review only - http://bmjopen.bmj.com/site/about/guidelines.xhtml

ISRCTN72038467

In addition, we acknowledge support from NIHR Health Protection Research Unit in Evaluation, and the Bristol Randomised Trials Collaboration (BRTC), a UKCRC Registered Clinical Trials Unit in receipt of NIHR Clinical Trials Units (CTU) support funding. The views expressed are those of the author and not necessarily those of the NHS, the NIHR or the Department of Health and Social Care. NM is supported by the National Institute for Drug Abuse [grant number R01 DA037773] and the University of California San Diego Center for AIDS Research (CFAR), a National Institute of Health (NIH) funded program [grant number P30 AI036214].

We would like to thank all those involved in PPI from both SDF and the Hepatitis C Trust.

Competing interests statement.

MH has received unrestricted honoraria for presenting at meetings from Abbvie, Gilead, MSD. NM has received unrestricted research grants and honoraria from Gilead and Merck. PV has received unrestricted honoraria for presenting at meetings from Abbvie and Gilead. PV has received unrestricted research grants from Gilead.

PTD has received unrestricted grants from Shire pharmaceuticals, Novo Nordisk and Gilead and is a member of the Scottish Medicines Consortium. HF has received an honorarium from MSD.

For peer review only

ISRCTN72038467

References

1. Williams R, Aspinall R, Bellis M, et al. Addressing liver disease in the UK: a blueprint for attaining excellence in health care and reducing premature mortality from lifestyle issues of excess consumption of alcohol, obesity, and viral hepatitis. *Lancet* 2014;384(9958):1953-97. doi: 10.1016/S0140-6736(14)61838-9
2. De Angelis D, Sweeting M, Ades A, et al. An evidence synthesis approach to estimating Hepatitis C prevalence in England and Wales. *Stat Methods Med Res* 2009;18(4):361-79. doi: 0962280208094691 [pii];10.1177/0962280208094691 [doi]
3. Harris RJ, Ramsay M, Hope VD, et al. Hepatitis C prevalence in England remains low and varies by ethnicity: an updated evidence synthesis. *Eur J Public Health* 2012;22(2):187-92. doi: ckr083 [pii];10.1093/eurpub/ckr083 [doi]
4. Hutchinson SJ, Roy KM, Wadd S, et al. Hepatitis C virus infection in Scotland: epidemiological review and public health challenges. *Scott Med J* 2006;51(2):8-15.
5. Prevost TC, Presanis AM, Taylor A, et al. Estimating the number of people with hepatitis C virus who have ever injected drugs and have yet to be diagnosed: an evidence synthesis approach for Scotland. *Addiction* 2015;110(8):1287-300. doi: 10.1111/add.12948
6. Hutchinson SJ, McIntyre PG, Molyneaux P, et al. Prevalence of hepatitis C among injectors in Scotland 1989-2000: declining trends among young injectors halt in the late 1990s. *Epidemiol Infect* 2002;128(3):473-77.
7. Martin NK, Foster GR, Vilar J, et al. HCV treatment rates and sustained viral response among people who inject drugs in seven UK sites: real world results and modelling of treatment impact. *J Viral Hepat* 2015;22(4):399. doi: 10.1111/jvh.12338
8. McLeod A, Weir A, Aitken C, et al. Rise in testing and diagnosis associated with Scotland's Action Plan on Hepatitis C and introduction of dried blood spot testing. *J Epidemiol Community Health* 2014;68(12):1182-8. doi: 10.1136/jech-2014-204451
9. Palmateer NE, Hutchinson SJ, Innes H, et al. Review and meta-analysis of the association between self-reported sharing of needles/syringes and hepatitis C virus prevalence and incidence among people who inject drugs in Europe. *IntJDrug Policy* 2013;24(2):85-100. doi: S0955-3959(12)00123-5 [pii];10.1016/j.drugpo.2012.08.006 [doi]
10. Palmateer NE, Taylor A, Goldberg DJ, et al. Rapid decline in HCV incidence among people who inject drugs associated with national scale-up in coverage of a combination of harm reduction interventions. *PLoS One* 2014;9(8):e104515. doi: 10.1371/journal.pone.0104515
11. Roy KM, Hutchinson SJ, Wadd S, et al. Hepatitis C virus infection among injecting drug users in Scotland: a review of prevalence and incidence data and the methods used to generate them. *Epidemiol Infect* 2007;135(3):433-42.
12. Turner KM, Hutchinson S, Vickerman P, et al. The impact of needle and syringe provision and opiate substitution therapy on the incidence of hepatitis C virus in injecting drug users: pooling of UK evidence. *Addiction* 2011;106(11):1978-88. doi: 10.1111/j.1360-0443.2011.03515.x [doi]
13. Harris RJ, Hope VD, Morongiu A, et al. Spatial mapping of hepatitis C prevalence in recent injecting drug users in contact with services. *Epidemiol Infect* 2011:1-10. doi: S0950268811001634 [pii];10.1017/S0950268811001634 [doi]
14. Hickman M, Hope V, Brady T, et al. Hepatitis C virus (HCV) prevalence, and injecting risk behaviour in multiple sites in England in 2004. *J Viral Hepat* 2007;14(9):645-52.
15. Hope VD, Hickman M, Ngui SL, et al. Measuring the incidence, prevalence and genetic relatedness of hepatitis C infections among a community recruited sample of injecting drug users, using dried blood spots. *J Viral Hepat* 2011;18:262-70. doi: JVH1297 [pii];10.1111/j.1365-2893.2010.01297.x [doi]

For peer review only - http://bmjopen.bmj.com/site/about/guidelines.xhtml

16. Sutton AJ, Gay NJ, Edmunds WJ, et al. Modelling the force of infection for hepatitis B and hepatitis C in injecting drug users in England and Wales. *BMC InfectDis* 2006;6:93. doi: 1471-2334-6-93 [pii];10.1186/1471-2334-6-93 [doi]
17. Sweeting MJ, Hope VD, Hickman M, et al. Hepatitis C infection among injecting drug users in England and Wales (1992-2006): there and back again? *Am J Epidemiol* 2009;170(3):352-60. doi: kwp141 [pii];10.1093/aje/kwp141 [doi]
18. Wiessing L, Ferri M, Grady B, et al. Hepatitis C virus infection epidemiology among people who inject drugs in Europe: a systematic review of data for scaling up treatment and prevention. *PLoS One* 2014;9(7):e103345. doi: 10.1371/journal.pone.0103345
19. Hickman MM, N., editor. *Hepatitis C among drug users in Europe: epidemiology, treatment and prevention*. Lisbon: EMCDDA, 2016.
20. Van Den Berg C, Smit C, Van BG, et al. Full participation in harm reduction programmes is associated with decreased risk for human immunodeficiency virus and hepatitis C virus: evidence from the Amsterdam Cohort Studies among drug users. *Addiction* 2007;102(9):1454-62. doi: ADD1912 [pii];10.1111/j.1360-0443.2007.01912.x [doi]
21. Hagan H, Pouget ER, Des Jarlais DC. A systematic review and meta-analysis of interventions to prevent hepatitis C virus infection in people who inject drugs. *J InfectDis* 2011;204(1):74-83. doi: jir196 [pii];10.1093/infdis/jir196 [doi]
22. Platt L, Reed J, Minozzi S, et al. Effectiveness of needle/syringe programmes and opiate substitution therapy in preventing HCV transmission among people who inject drugs. *Cochrane Database Syst Rev* 2016;2016(1) doi: 10.1002/14651858.CD012021
23. Hutchinson SJ, Dillon JF, Fox R, et al. Expansion of HCV treatment access to people who have injected drugs through effective translation of research into public health policy: Scotland's experience. *Int J Drug Policy* 2015 doi: 10.1016/j.drugpo.2015.05.019
24. Fraser H, Mukandavire C, Martin NK, et al. Modelling the impact of a national scale-up of interventions on hepatitis C virus transmission among people who inject drugs in Scotland. *Addiction* 2018 doi: 10.1111/add.14267 [published Online First: 2018/05/22]
25. de Vos AS, van der Helm JJ, Matser A, et al. Decline in incidence of HIV and hepatitis C virus infection among injecting drug users in Amsterdam; evidence for harm reduction? *Addiction* 2013;108(6):1070-81. doi: 10.1111/add.12125
26. Vickerman P, Martin N, Turner K, et al. Can needle and syringe programmes and opiate substitution therapy achieve substantial reductions in hepatitis C virus prevalence? Model projections for different epidemic settings. *Addiction* 2012;107(11):1984-95. doi: 10.1111/j.1360-0443.2012.03932.x [doi]
27. Dore GJ, Feld JJ. Hepatitis C virus therapeutic development: in pursuit of "perfectovir". *Clin Infect Dis* 2015;60(12):1829-36. doi: 10.1093/cid/civ197
28. Gogela NA, Lin MV, Wisocky JL, et al. Enhancing our understanding of current therapies for hepatitis C virus (HCV). *Curr HIV/AIDS Rep* 2015;12(1):68-78. doi: 10.1007/s11904-014-0243-7
29. Walker DR, Pedrosa MC, Manthena SR, et al. Early View of the Effectiveness of New Direct-Acting Antiviral (DAA) Regimens in Patients with Hepatitis C Virus (HCV). *Adv Ther* 2015;32(11):1117-27. doi: 10.1007/s12325-015-0258-5
30. Hellard M, Doyle JS, Sacks-Davis R, et al. Eradication of hepatitis C infection: The importance of targeting people who inject drugs. *Hepatology* 2014;59(2):366-69. doi: 10.1002/hep.26623
31. Hagan LM, Wolpe PR, Schinazi RF. Treatment as prevention and cure towards global eradication of hepatitis C virus. *Trends in Microbiology* 2013;21(12):625-33. doi: http://dx.doi.org/10.1016/j.tim.2013.09.008
32. Grebely J, Matthews GV, Lloyd AR, et al. Elimination of hepatitis C virus infection among people who inject drugs through treatment as prevention: feasibility and future requirements. *Clin Infect Dis* 2013;57(7):1014-20. doi: 10.1093/cid/cit377

For peer review only - http://bmjopen.bmj.com/site/about/guidelines.xhtml

ISRCTN72038467

33. Bruggmann P. Treatment as prevention: The breaking of taboos is required in the fight against hepatitis C among people who inject drugs. *Hepatology* 2013;58(5):1523-25. doi: 10.1002/hep.26539
34. Martin NK, Vickerman P, Grebely J, et al. HCV treatment for prevention among people who inject drugs: Modeling treatment scale-up in the age of direct-acting antivirals. *Hepatology* 2013 doi: 10.1002/hep.26431 [doi]
35. Wedemeyer H, Duberg AS, Buti M, et al. Strategies to manage hepatitis C virus (HCV) disease burden. *J Viral Hepat* 2014;21 Suppl 1:60-89. doi: 10.1111/jvh.12249
36. Martin NK, Vickerman P, Dore GJ, et al. The hepatitis C virus epidemics in key populations (including people who inject drugs, prisoners and MSM): the use of direct-acting antivirals as treatment for prevention. *Current opinion in HIV and AIDS* 2015;10(5):374-80. doi: 10.1097/COH.0000000000000179
37. Hickman M, De Angelis D, Vickerman P, et al. HCV Treatment as Prevention in People Who Inject Drugs–testing the evidence. *Current opinion in infectious diseases* 2015;28(6):576.
38. Hayes R, Ayles H, Beyers N, et al. HPTN 071 (PopART): rationale and design of a cluster-randomised trial of the population impact of an HIV combination prevention intervention including universal testing and treatment - a study protocol for a cluster randomised trial. *Trials* 2014;15:57. doi: 10.1186/1745-6215-15-57
39. Fraser H, Zibbell J, Hoerger T, et al. Scaling-up HCV prevention and treatment interventions in rural United States-model projections for tackling an increasing epidemic. *Addiction* 2018;113(1):173-82. doi: 10.1111/add.13948 [published Online First: 2017/07/25]
40. Organization WH. Global Health Sector Strategy on Viral Hepatitis 2016-2021: Towards ending viral hepatitis. In: Organization WH, ed. Geneva: WHO, 2016.
41. Innes HA, McDonald SA, Dillon JF, et al. Toward a more complete understanding of the association between a hepatitis C sustained viral response and cause-specific outcomes. *Hepatology* 2015;62(2):355-64. doi: 10.1002/hep.27766
42. Innes HA, Hutchinson SJ, Allen S, et al. Excess liver-related morbidity of chronic hepatitis C patients, who achieve a sustained viral response, and are discharged from care. *Hepatology* 2011;54(5):1547-58. doi: 10.1002/hep.24561
43. Innes H, Goldberg D, Dillon J, et al. Strategies for the treatment of Hepatitis C in an era of interferon-free therapies: what public health outcomes do we value most? *Gut* 2014 doi: 10.1136/gutjnl-2014-308166
44. Harris RJ, Martin NK, Rand E, et al. New treatments for hepatitis C virus (HCV): scope for preventing liver disease and HCV transmission in England. *J Viral Hepat* 2016 doi: 10.1111/jvh.12529
45. European Association for the Study of the L. EASL recommendations on treatment of hepatitis C 2014. *J Hepatol* 2014;61(2):373-95. doi: 10.1016/j.jhep.2014.05.001
46. European Association for the Study of the Liver. Electronic address eee. EASL Recommendations on Treatment of Hepatitis C 2015. *J Hepatol* 2015;63(1):199-236. doi: 10.1016/j.jhep.2015.03.025
47. Panel AIHG. Hepatitis C guidance: AASLD-IDSA recommendations for testing, managing, and treating adults infected with hepatitis C virus. *Hepatology* 2015 doi: 10.1002/hep.27950
48. Grebely J, Robaeys G, Bruggmann P, et al. Recommendations for the management of hepatitis C virus infection among people who inject drugs. *Int J Drug Policy* 2015;26(10):1028-38. doi: 10.1016/j.drugpo.2015.07.005
49. European Association for the Study of the Liver. Electronic address eee. EASL Recommendations on Treatment of Hepatitis C 2016. *J Hepatol* 2016 doi: 10.1016/j.jhep.2016.09.001
50. Martin NK, Vickerman P, Dore GJ, et al. Prioritization of HCV treatment in the direct-acting antiviral era: An economic evaluation. *J Hepatol* 2016;65(1):17-25. doi: 10.1016/j.jhep.2016.02.007

For peer review only - http://bmjopen.bmj.com/site/about/guidelines.xhtml

For peer review only

ISRCTN72038467

51. Aleman S, Rahbin N, Weiland O, et al. A risk for hepatocellular carcinoma persists long-term after sustained virologic response in patients with hepatitis C-associated liver cirrhosis. *Clin Infect Dis* 2013;57(2):230-6. doi: 10.1093/cid/cit234
52. van der Meer AJ, Veldt BJ, Feld JJ, et al. Association between sustained virological response and all-cause mortality among patients with chronic hepatitis C and advanced hepatic fibrosis. *JAMA* 2012;308(24):2584-93. doi: 10.1001/jama.2012.144878
53. Innes H, Hutchinson SJ, Obel N, et al. Liver mortality attributable to chronic hepatitis C virus infection in Denmark and Scotland--using spontaneous resolvers as the benchmark comparator. *Hepatology* 2016;63(5):1506-16. doi: 10.1002/hep.28458
54. Fraser H, Martin NK, Brummer-Korvenkontio H, et al. Model projections on the impact of HCV treatment in the prevention of HCV transmission among people who inject drugs in Europe. *J Hepatol* 2018;68(3):402-11. doi: 10.1016/j.jhep.2017.10.010 [published Online First: 2017/10/31]
55. Schulkind J, Stephens B, Ahmad F, et al. High response and re-infection rates among people who inject drugs treated for hepatitis C in a community needle and syringe programme. *Journal of Viral Hepatitis* 2018 doi: 10.1111/jvh.13035
56. MacArthur GJ, Minozzi S, Martin N, et al. Opiate substitution treatment and HIV transmission in people who inject drugs: systematic review and meta-analysis. *BMJ* 2012;345:e5945.
57. MacArthur GJ, van VE, Palmateer N, et al. Interventions to prevent HIV and Hepatitis C in people who inject drugs: A review of reviews to assess evidence of effectiveness. *IntJ Drug Policy* 2013 doi: S0955-3959(13)00116-3 [pii];10.1016/j.drugpo.2013.07.001 [doi]
58. Palmateer N, Hutchinson S, McAllister G, et al. Risk of transmission associated with sharing drug injecting paraphernalia: analysis of recent hepatitis C virus (HCV) infection using cross-sectional survey data. *J Viral Hepat* 2014;21(1):25-32. doi: 10.1111/jvh.12117
59. Palmateer N, Kimber J, Hickman M, et al. Evidence for the effectiveness of sterile injecting equipment provision in preventing hepatitis C and human immunodeficiency virus transmission among injecting drug users: a review of reviews. *Addiction* 2010;105(5):844-59. doi: ADD2888 [pii];10.1111/j.1360-0443.2009.02888.x [doi]
60. Presanis AM, Gill ON, Chadborn TR, et al. Insights into the rise in HIV infections, 2001 to 2008: a Bayesian synthesis of prevalence evidence. *AIDS* 2010;24(18):2849-58. doi: 10.1097/QAD.0b013e32834021ed [doi]
61. England PH. Hepatitis C in the UK 2016 report: Working towards its elimination as a major public health threat. In: Harris HE, ed. London: Public Health England, 2016.
62. Health Protection Scotland UotWoS, Glasgow Caledonian University and the West of Scotland Specialist Virology Centre. The Needle Exchange Surveillance Initiative: Prevalence of blood-borne viruses and injecting risk behaviours among people who inject drugs attending injecting equipment provision services in Scotland, 2008-09 to 2015-16. In: Scotland HP, ed. Glasgow, 2017.
63. Brant LJ, Hurrelle M, Balogun MA, et al. Sentinel laboratory surveillance of hepatitis C antibody testing in England: understanding the epidemiology of HCV infection. *Epidemiol Infect* 2007;135(3):417-26. doi: 10.1017/S0950268806006832
64. Brant LJ, Hurrelle M, Balogun MA, et al. Where are people being tested for anti-HCV in England? Results from sentinel laboratory surveillance. *J Viral Hepat* 2008;15(10):729-39. doi: 10.1111/j.1365-2893.2008.01000.x
65. Lattimore S, Irving W, Collins S, et al. Using surveillance data to determine treatment rates and outcomes for patients with chronic hepatitis C virus infection. *Hepatology* 2014;59(4):1343-50. doi: 10.1002/hep.26926
66. Treloar C, Newland J, Rance J, et al. Uptake and delivery of hepatitis C treatment in opiate substitution treatment: perceptions of clients and health professionals. *J Viral Hepat* 2010;17(12):839-44. doi: JVH1250 [pii];10.1111/j.1365-2893.2009.01250.x [doi]

For peer review only - http://bmjopen.bmj.com/site/about/guidelines.xhtml

ISRCTN72038467

67. Treloar C, Rance J, Dore GJ, et al. Barriers and facilitators for assessment and treatment of hepatitis C virus infection in the opioid substitution treatment setting: insights from the ETHOS study. *J Viral Hepat* 2014;21(8):560-7. doi: 10.1111/jvh.12183
68. Harris M, Rhodes T. Hepatitis C treatment access and uptake for people who inject drugs: a review mapping the role of social factors. *Harm Reduct J* 2013;10:7. doi: 10.1186/1477-7517-10-7
69. Swan D, Long J, Carr O, et al. Barriers to and facilitators of hepatitis C testing, management, and treatment among current and former injecting drug users: a qualitative exploration. *AIDS Patient Care STDS* 2010;24(12):753-62. doi: 10.1089/apc.2010.0142
70. Rhodes T, Harris M, Martin A. Negotiating access to medical treatment and the making of patient citizenship: the case of hepatitis C treatment. *Sociol Health Illn* 2013;35(7):1023-44. doi: 10.1111/1467-9566.12018
71. Harris M, Albers E, Swan T. The promise of treatment as prevention for hepatitis C: Meeting the needs of people who inject drugs? *Int J Drug Policy* 2015;26(10):963-9. doi: 10.1016/j.drugpo.2015.05.005
72. Rance J, Treloar C, Group ES. 'Not just methadone Tracy': transformations in service-user identity following the introduction of hepatitis C treatment into Australian opiate substitution settings. *Addiction* 2014;109(3):452-9.
73. Treloar C, Rance J, Backmund M. Understanding barriers to hepatitis C virus care and stigmatization from a social perspective. *Clin Infect Dis* 2013;57 Suppl 2:S51-5. doi: 10.1093/cid/cit263
74. Treloar C, Rance J, Bryant J, et al. Harm reduction workers and the challenge of engaging couples who inject drugs in hepatitis C prevention. *Drug Alcohol Depend* 2016;168:170-75. doi: 10.1016/j.drugalcdep.2016.09.010
75. Treloar C, Rance J, Bryant J, et al. Understanding decisions made about hepatitis C treatment by couples who inject drugs. *J Viral Hepat* 2016;23(2):89-95. doi: 10.1111/jvh.12451
76. Jack K, Islip N, Linsley P, et al. Prison officers' views about hepatitis C testing and treatment: A qualitative enquiry. *J Clin Nurs* 2016 doi: 10.1111/jocn.13489
77. Rich ZC, Chu C, Mao J, et al. Facilitators of HCV treatment adherence among people who inject drugs: a systematic qualitative review and implications for scale up of direct acting antivirals. *BMC Public Health* 2016;16:994. doi: 10.1186/s12889-016-3671-z
78. Clark JA, Gifford AL. Resolute efforts to cure hepatitis C: Understanding patients' reasons for completing antiviral treatment. *Health (London)* 2015;19(5):473-89. doi: 10.1177/1363459314555237
79. Batchelder AW, Peyser D, Nahvi S, et al. "Hepatitis C treatment turned me around:" Psychological and behavioral transformation related to hepatitis C treatment. *Drug Alcohol Depend* 2015;153:66-71. doi: 10.1016/j.drugalcdep.2015.06.007
80. Harris M. Managing expense and expectation in a treatment revolution: Problematizing prioritisation through an exploration of hepatitis C treatment 'benefit'. *Int J Drug Policy* 2017;47:161-68. doi: 10.1016/j.drugpo.2017.03.015 [published Online First: 2017/04/30]
81. Aspinall EJ, Hutchinson SJ, Janjua NZ, et al. Trends in mortality after diagnosis of hepatitis C virus infection: an international comparison and implications for monitoring the population impact of treatment. *J Hepatol* 2015;62(2):269-77. doi: 10.1016/j.jhep.2014.09.001
82. Aspinall EJ, Corson S, Doyle JS, et al. Treatment of hepatitis C virus infection among people who are actively injecting drugs: a systematic review and meta-analysis. *Clin Infect Dis* 2013;57 Suppl 2:S80-9. doi: 10.1093/cid/cit306
83. Judd A, Parry J, Hickman M, et al. Evaluation of a modified commercial assay in detecting antibody to hepatitis C virus in oral fluids and dried blood spots. *J Med Virol* 2003;71(1):49-55.

For peer review only - http://bmjopen.bmj.com/site/about/guidelines.xhtml

ISRCTN72038467

84. Bennett S, Gunson RN, McAllister GE, et al. Detection of hepatitis C virus RNA in dried blood spots. *J Clin Virol* 2012;54(2):106-09. doi: S1386-6532(12)00053-4 [pii];10.1016/j.jcv.2012.02.004 [doi]
85. Martin NK, Vickerman P, Brew IF, et al. Is increased hepatitis C virus case-finding combined with current or 8-week to 12-week direct-acting antiviral therapy cost-effective in UK prisons? A prevention benefit analysis. *Hepatology* 2016;63(6):1796-808. doi: 10.1002/hep.28497
86. McDonald SA, Innes HA, Hayes PC, et al. What is the impact of a country-wide scale-up in antiviral therapy on the characteristics and sustained viral response rates of patients treated for hepatitis C? *J Hepatol* 2015;62(2):262-8. doi: 10.1016/j.jhep.2014.08.046 [published Online First: 2014/09/10]
87. Ward Z, Platt L, Sweeney S, et al. Impact of current and scaled-up levels of hepatitis C prevention and treatment interventions for people who inject drugs in three UK settings-what is required to achieve the WHO's HCV elimination targets? *Addiction* 2018 doi: 10.1111/add.14217 [published Online First: 2018/05/19]
88. Brodersen KH, Gallusser F, Koehler J, et al. Inferring causal impact using Bayesian structural time-series models. 2015:247-74. doi: 10.1214/14-AOAS788
89. Scott S VH. Predicting the present with bayesian structural time series. *International Journal of Mathematical Modelling and Numerical Optimisation* 2014;2014(5):23.
90. Rance J, Newland J, Hopwood M, et al. The politics of place(ment): problematising the provision of hepatitis C treatment within opiate substitution clinics. *Soc Sci Med* 2012;74(2):245-53. doi: S0277-9536(11)00644-7 [pii];10.1016/j.socscimed.2011.10.003 [doi]
91. Lankenau SE, Sanders B, Hathazi D, et al. Recruiting and retaining mobile young injection drug users in a longitudinal study. *Subst Use Misuse* 2010;45(5):684-99. doi: 10.3109/10826081003594914
92. Stewart KE, Wright PB, Sims D, et al. The "translators": engaging former drug users as key research staff to design and implement a risk reduction program for rural cocaine users. *Subst Use Misuse* 2012;47(5):547-54. doi: 10.3109/10826084.2011.644379
93. Harris M. "Three in the Room": Embodiment, Disclosure, and Vulnerability in Qualitative Research. *Qual Health Res* 2015;25(12):1689-99. doi: 10.1177/1049732314566324
94. Braun V, Clarke V. Using thematic analysis in psychology. *Qualitative Research in Psychology* 2006;3(2):77-101. doi: 10.1191/1478088706qp063oa
95. Rogers EM. Diffusion of preventive innovations. *Addict Behav* 2002;27(6):989-93.
96. Cane J, O'Connor D, Michie S. Validation of the theoretical domains framework for use in behaviour change and implementation research. *Implement Sci* 2012;7:37. doi: 10.1186/1748-5908-7-37
97. Innes HA, Hutchinson SJ, Allen S, et al. Ranking predictors of a sustained viral response for patients with chronic hepatitis C treated with pegylated interferon and ribavirin in Scotland. *Eur J Gastroenterol Hepatol* 2012;24(6):646-55. doi: 10.1097/MEG.0b013e32835201a4
98. McDonald SA, Hutchinson SJ, Bird SM, et al. Excess morbidity in the hepatitis C-diagnosed population in Scotland, 1991-2006. *Epidemiol Infect* 2011;139(3):344-53. doi: 10.1017/S0950268810001421
99. McDonald SA, Hutchinson SJ, Bird SM, et al. A record-linkage study of the development of hepatocellular carcinoma in persons with hepatitis C infection in Scotland. *Br J Cancer* 2008;99(5):805-10.
100. McDonald SA, Hutchinson SJ, Bird SM, et al. A population-based record linkage study of mortality in hepatitis C-diagnosed persons with or without HIV coinfection in Scotland. *Stat Methods Med Res* 2009;18(3):271-83. doi: 10.1177/0962280208094690
101. Gao L, Dimitropoulou P, Robertson JR, et al. Risk-factors for methadone-specific deaths in Scotland's methadone-prescription clients between 2009 and 2013. *Drug Alcohol Depend* 2016;167:214-23. doi: 10.1016/j.drugalcdep.2016.08.627

For peer review only - http://bmjopen.bmj.com/site/about/guidelines.xhtml

ISRCTN72038467

102. Bird SM, Fischbacher CM, Graham L, et al. Impact of opioid substitution therapy for Scotland's prisoners on drug-related deaths soon after prisoner release. *Addiction* 2015;110(10):1617-24. doi: 10.1111/add.12969
103. Bird SM, Hutchinson SJ. Male drugs-related deaths in the fortnight after release from prison: Scotland, 1996-99. *Addiction* 2003;98(2):185-90.
104. Alvarez-Madrazo S, McTaggart S, Nangle C, et al. Data Resource Profile: The Scottish National Prescribing Information System (PIS). *Int J Epidemiol* 2016;45(3):714-15f. doi: 10.1093/ije/dyw060
105. Graham L, Fischbacher CM, Stockton D, et al. Understanding extreme mortality among prisoners: a national cohort study in Scotland using data linkage. *Eur J Public Health* 2015;25(5):879-85. doi: 10.1093/eurpub/cku252
106. Merrall EL, Bird SM, Hutchinson SJ. Mortality of those who attended drug services in Scotland 1996-2006: record-linkage study. *Int J Drug Policy* 2012;23(1):24-32. doi: 10.1016/j.drugpo.2011.05.010
107. McDonald SA, Hutchinson SJ, Bird SM, et al. Hospitalisation for an alcohol-related cause among injecting drug users in Scotland: increased risk following diagnosis with hepatitis C infection. *Int J Drug Policy* 2011;22(1):63-9. doi: 10.1016/j.drugpo.2010.04.003
108. de Vos AS, Prins M, Kretzschmar ME. Hepatitis C virus treatment as prevention among injecting drug users: who should we cure first? *Addiction* 2015;110(6):975-83. doi: 10.1111/add.12842
109. Boily MC, Lowndes CM, Vickerman P, et al. Evaluating large-scale HIV prevention interventions: study design for an integrated mathematical modelling approach. *Sex TransmInfect* 2007;83(7):582-89. doi: sti.2007.027516 [pii];10.1136/sti.2007.027516 [doi]
110. Boily MC, Pickles M, Lowndes CM, et al. Positive impact of a large-scale HIV prevention program among female sex workers and clients in Karnataka state, India. *AIDS* 2013 doi: 10.1097/QAD.0b013e32835fba81 [doi]
111. Hallett TB, Gregson S, Mugurungi O, et al. Assessing evidence for behaviour change affecting the course of HIV epidemics: a new mathematical modelling approach and application to data from Zimbabwe. *Epidemics* 2009;1(2):108-17. doi: 10.1016/j.epidem.2009.03.001
112. Pickles M, Boily MC, Vickerman P, et al. Assessment of the population-level effectiveness of the Avahan HIV-prevention programme in South India: a preplanned, causal-pathway-based modelling analysis. *Lancet Glob Health* 2013;1(5):e289-99. doi: 10.1016/S2214-109X(13)70083-4
113. Martin NK, Hickman M, Miners A, et al. Cost-effectiveness of HCV case-finding for people who inject drugs via dried blood spot testing in specialist addiction services and prisons. *BMJ open* 2013;3(8) doi: 10.1136/bmjopen-2013-003153
114. Martin NK, Miners A, Vickerman P. Assessing the cost-effectiveness of interventions aimed at promoting and offering hepatitis C testing to injecting drug users: An economic modelling report. London, 2012.
115. Martin NK, Pitcher AB, Vickerman P, et al. Optimal control of hepatitis C antiviral treatment programme delivery for prevention amongst a population of injecting drug users. *PLoS One* 2011;6(8):e22309. doi: 10.1371/journal.pone.0022309 [doi];PONE-D-10-01568 [pii]
116. Martin NK, Vickerman P, Miners A, et al. The cost-effectiveness of HCV antiviral treatment for injecting drug user populations. *Hepatology* 2012;55(1):49-57. doi: 10.1002/hep.24656 [doi]
117. Martin NK, Vickerman P, Miners A, et al. How cost-effective is hepatitis C virus treatment for people who inject drugs? *Journal of gastroenterology and hepatology* 2013;28(4):590-2. doi: 10.1111/jgh.12113
118. Kimber J, Copeland L, Hickman M, et al. Survival and cessation in injecting drug users: prospective observational study of outcomes and effect of opiate substitution treatment. *BMJ* 2010;341:c3172.
119. McDonald SA, Hutchinson SJ, Palmateer NE, et al. Decrease in health-related quality of life associated with awareness of hepatitis C virus infection among people who inject drugs in

For peer review only - http://bmjopen.bmj.com/site/about/guidelines.xhtml

ISRCTN72038467

Scotland. *JHepatol* 2013;58(3):460-66. doi: S0168-8278(12)00836-7 [pii];10.1016/j.jhep.2012.11.004 [doi]

120. Wright M, Grieve R, Roberts J, et al. Health benefits of antiviral therapy for mild chronic hepatitis C: randomised controlled trial and economic evaluation. *Health Technol Assess* 2006;10(21):1-113, iii. doi: 95-24-03 [pii]

121. Harrison GI, Murray K, Gore R, et al. The Hepatitis C Awareness Through to Treatment (HepCATT) study: Improving the cascade of care for hepatitis C virus-infected people who inject drugs in England. *Addiction* 2019 doi: 10.1111/add.14569 [published Online First: 2019/01/30]

For peer review only

For peer review only - http://bmjopen.bmj.com/site/about/guidelines.xhtml

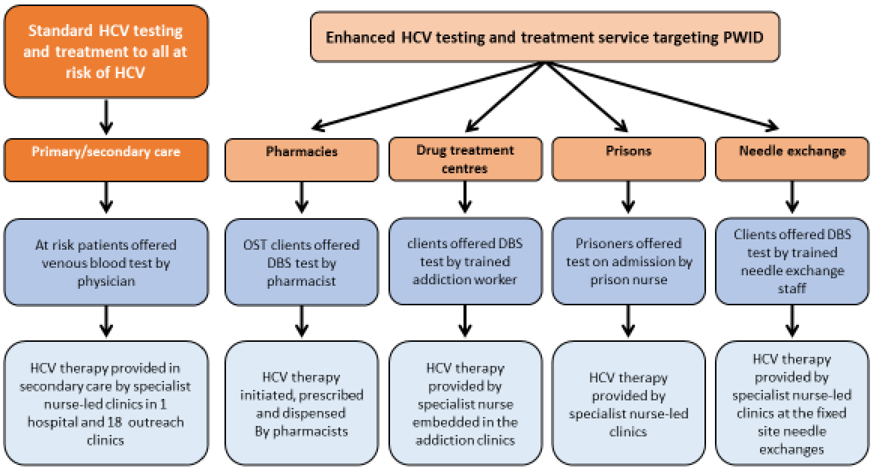

PWID defined as those who either (a) are currently injecting drugs, (b) have ever injected drugs and are currently on opioid substitute therapy, or (c) have ever injected drugs and are currently in prison

DBS: dried blood spot; OST: opioid substitution therapies; PWID: people who inject drugs

For peer review only

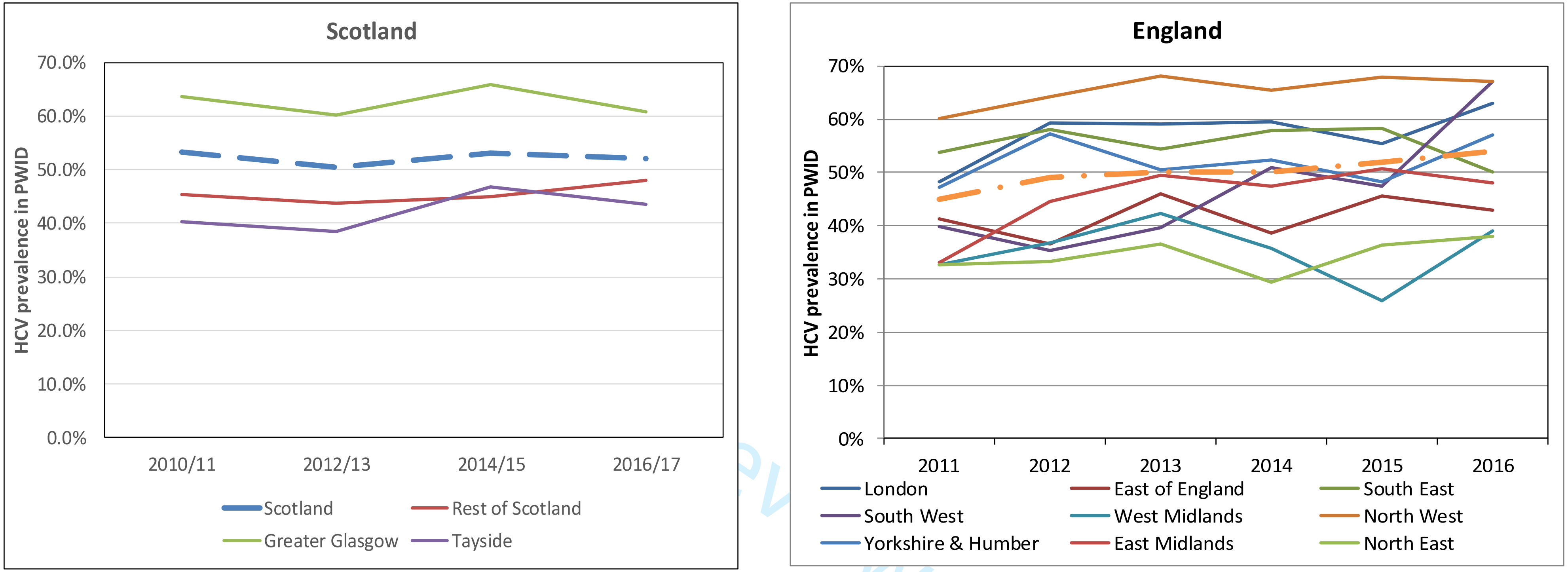
Scotland
70.0%
60.0%
50.0%
40.0%
30.0%
20.0%
10.0%
0.0%
HCV prevalence in PWID
2010/11
2012/13
2014/15
2016/17
Scotland
Rest of Scotland
Greater Glasgow
Tayside
England
70%
60%
50%
40%
30%
20%
10%
0%
HCV prevalence in PWID
2011
2012
2013
2014
2015
2016
London
East of England
South East
South West
West Midlands
North West
Yorkshire & Humber
East Midlands
North East

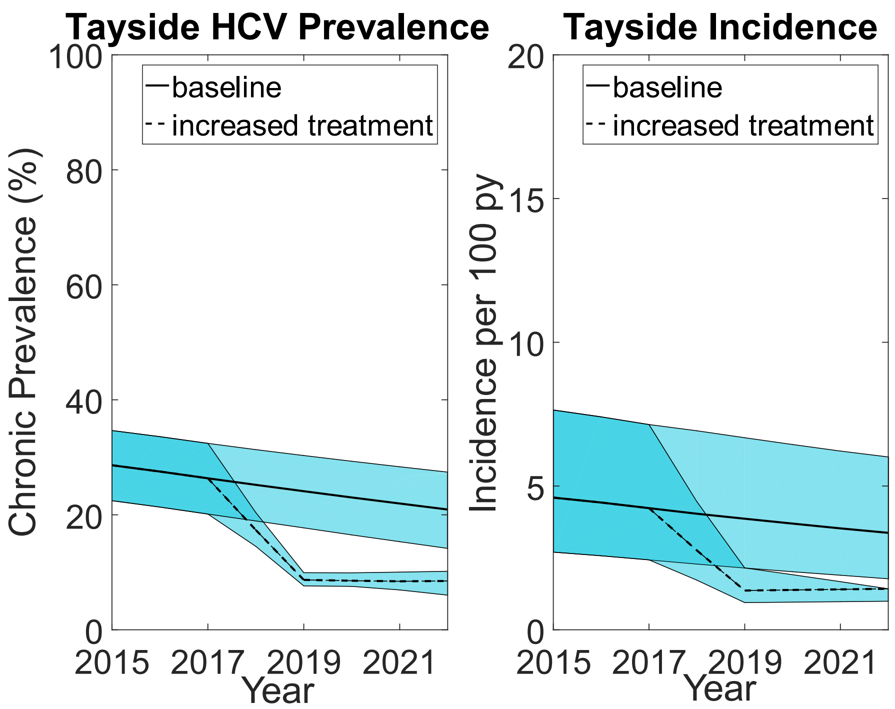

Tayside HCV Prevalence
Chronic Prevalence (%)
100
80
60
40
20
0
2015
2017
2019
2021
Year
baseline
increased treatment
Tayside Incidence
Incidence per 100 py
20
15
10
5
0
2015
2017
2019
2021
Year
baseline
increased treatment

iew only

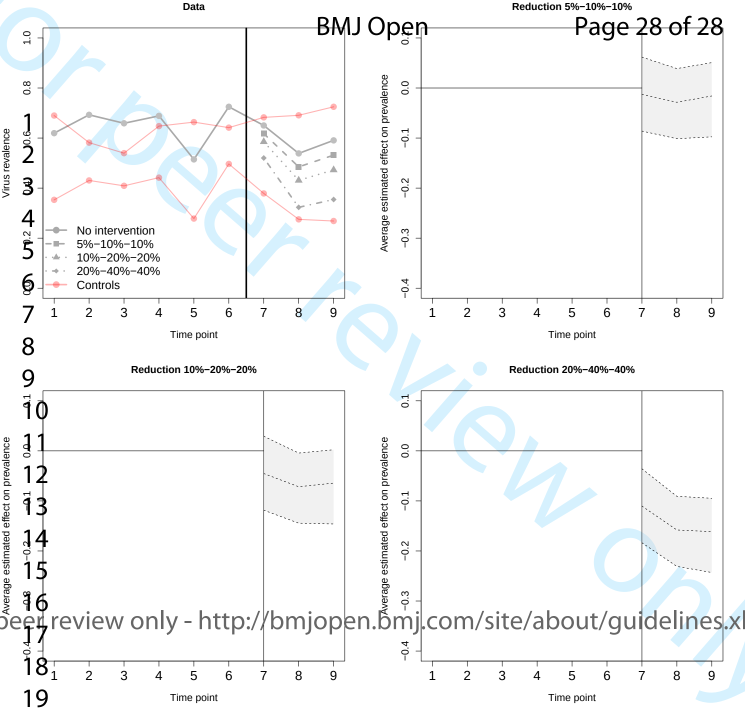

For peer review only - http://bmjopen.bmj.com/site/about/guidelines.xhtml

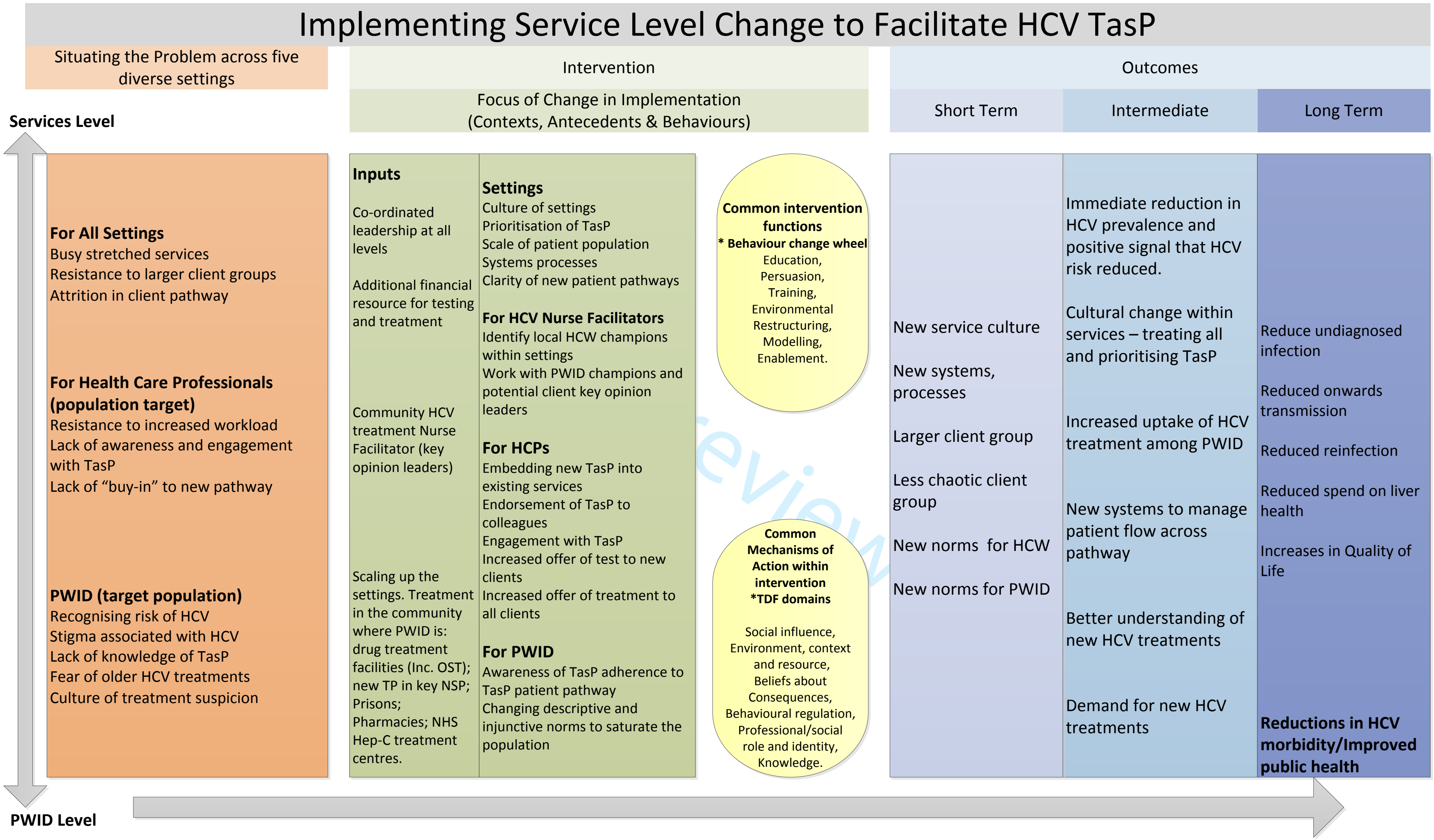
Implementing Service Level Change to Facilitate HCV TasP
Situating the Problem across five diverse settings
Intervention
Focus of Change in Implementation (Contexts, Antecedents & Behaviours)
Outcomes
Short Term
Intermediate
Long Term
Services Level
PWID Level
For All Settings
Busy stretched services
Resistance to larger client groups
Attrition in client pathway
For Health Care Professionals (population target)
Resistance to increased workload
Lack of awareness and engagement with TasP
Lack of "buy-in" to new pathway
PWID (target population)
Recognising risk of HCV
Stigma associated with HCV
Lack of knowledge of TasP
Fear of older HCV treatments
Culture of treatment suspicion
Inputs
Co-ordinated leadership at all levels
Additional financial resource for testing and treatment
Community HCV treatment Nurse Facilitator (key opinion leaders)
Scaling up the settings. Treatment in the community where PWID is: drug treatment facilities (Inc. OST); new TP in key NSP; Prisons; Pharmacies; NHS Hep-C treatment centres.
Settings
Culture of settings
Prioritisation of TasP
Scale of patient population
Systems processes
Clarity of new patient pathways
For HCV Nurse Facilitators
Identify local HCW champions within settings
Work with PWID champions and potential client key opinion leaders
For HCPs
Embedding new TasP into existing services
Endorsement of TasP to colleagues
Engagement with TasP
Increased offer of test to new clients
Increased offer of treatment to all clients
For PWID
Awareness of TasP adherence to TasP patient pathway
Changing descriptive and injunctive norms to saturate the population
Common intervention functions
* Behaviour change wheel
Education, Persuasion, Training, Environmental Restructuring, Modelling, Enablement.
Common Mechanisms of Action within intervention
*TDF domains
Social influence, Environment, context and resource, Beliefs about Consequences, Behavioural regulation, Professional/social role and identity, Knowledge.
New service culture
New systems, processes
Larger client group
Less chaotic client group
New norms for HCW
New norms for PWID
Immediate reduction in HCV prevalence and positive signal that HCV risk reduced.
Cultural change within services – treating all and prioritising TasP
Increased uptake of HCV treatment among PWID
New systems to manage patient flow across pathway
Better understanding of new HCV treatments
Demand for new HCV treatments
Reduce undiagnosed infection
Reduced onwards transmission
Reduced reinfection
Reduced spend on liver health
Increases in Quality of Life
Reductions in HCV morbidity/Improved public health
